# A genome-wide Asian genetic map and ethnic comparison: The GENDISCAN study

**DOI:** 10.1186/1471-2164-9-554

**Published:** 2008-11-25

**Authors:** Young Seok Ju, Hansoo Park, Mi Kyeong Lee, Jong-Il Kim, Joohon Sung, Sung-Il Cho, Jeong-Sun Seo

**Affiliations:** 1 Department of Biochemistry and Molecular Biology, College of Medicine, Seoul National University, Seoul, Korea; 2 Psoma Therapeutics, Seoul, Korea; 3 Department of Epidemiology, Graduate School of Public Health and Institute of Health and Environment, Seoul National University, Seoul, Korea; 4 ILCHUN Genomic Medicine Institute, Medical Research Center, Seoul National University, Seoul, Korea

## Abstract

**Background:**

Genetic maps provide specific positions of genetic markers, which are required for performing genetic studies. Linkage analyses of Asian families have been performed with Caucasian genetic maps, since appropriate genetic maps of Asians were not available. Different ethnic groups may have different recombination rates as a result of genomic variations, which would generate misspecification of the genetic map and reduce the power of linkage analyses.

**Results:**

We constructed the genetic map of a Mongolian population in Asia with CRIMAP software. This new map, called the GENDISCAN map, is based on genotype data collected from 1026 individuals of 73 large Mongolian families, and includes 1790 total and 1500 observable meioses. The GENDISCAN map provides sex-averaged and sex-specific genetic positions of 1039 microsatellite markers in Kosambi centimorgans (cM) with physical positions. We also determined 95% confidence intervals of genetic distances of the adjacent marker intervals.

Genetic lengths of the whole genome, chromosomes and adjacent marker intervals are compared with those of Rutgers Map v.2, which was constructed based on Caucasian populations (Centre d'Etudes du Polymorphisme Humain (CEPH) and Icelandic families) by mapping methods identical to those of the GENDISCAN map, CRIMAP software and the Kosambi map function. Mongolians showed approximately 1.9 fewer recombinations per meiosis than Caucasians. As a result, genetic lengths of the whole genome and chromosomes of the GENDISCAN map are shorter than those of Rutgers Map v.2. Thirty-eight marker intervals differed significantly between the Mongolian and Caucasian genetic maps.

**Conclusion:**

The new GENDISCAN map is applicable to the genetic study of Asian populations. Differences in the genetic distances between the GENDISCAN and Caucasian maps could facilitate elucidation of genomic variations between different ethnic groups.

## Background

Genetic maps provide specific positions of genetic markers, which are required for performing genetic studies. Linkage analyses, which aim to identify genetic loci related to human phenotypes and complex diseases, have been performed with Caucasian genetic maps even in Asian populations, because no comprehensive Asian genetic maps with dense markers have yet been introduced. Since multipoint methods are frequently used in linkage analyses, it is important to use correct maps for the population being studied [1].

Distance between adjacent genetic markers in genetic maps is calculated from average recombination rates between markers during meiosis with map functions. The Kosambi map function is widely used nowadays.

Genetic mapping was first introduced using restriction fragment length polymorphism (RFLP) markers [2], followed by genome-wide human genetic maps with more informative microsatellite markers. The Genethon [3] and Marshfield [4] maps were created from eight CEPH families [5], using 5264 and 8325 genetic markers, respectively, but few (< 190) meioses. The deCODE map utilized 1257 meioses in 146 Icelandic families with 5136 markers [6]. In addition, several combined genetic maps were generated based on the CEPH and Icelandic populations [7-10].

Other than for Caucasians, however, there are few human genetic maps for different ethnic groups. Although genetic maps of four different ethnic groups (African Americans, Mexican Americans, East Asians, and Whites) were recently constructed, the number of markers was quite small (n = 353) and the maps were constructed based on nuclear families [11]. Misspecification of genetic maps may reduce the power of linkage analyses [1,12], and different ethnic groups may have different recombination rates [13]. Therefore, separate genetic maps for Asian populations are needed to investigate the Asian genome more precisely.

We constructed an Asian genetic map with 1039 microsatellite markers using 1026 genotyped individuals in 73 large Mongolian families. This study was undertaken as a part of GENDISCAN (GENe DIScovery for Complex traits in isolated large families of Asians of Northeast) project. The construction of an Asian genetic map may be applicable to further linkage studies of Asian ethnic groups as well as to understanding the genomic variations between Asians and Caucasians with megabase resolution.

## Results

Files providing details of the GENDISCAN map (*e.g*., the genetic/physical positions, the genetic distance of intervals, the 95% confidence intervals of the genetic distance of intervals, the genetic distance of Rutgers Map v.2 intervals, the p-values denoting the significance levels for differences in the genetic distance between the GENDISCAN map and Rutgers Map v.2 intervals, the heterozygosity of markers, the number of informative meioses of markers and the number of informative meioses between markers) are available. See additional file 1: Details of GENDISCAN map.

We genotyped 73 families, consisting of 1446 family members and a total of 1790 meioses. Among the 1446 family members, 1026 were genotyped and 1500 meioses became available for investigation. Considering the heterozygosity of the 1039 microsatellite markers genotyped in this study, 47 to 1098 informative meioses of each marker (average, 711.5) were obtained using CRIMAP software [14]. Only 18 markers (1.7%) showed fewer than 400 informative meioses.

The GENDISCAN map shows the genetic positions of the markers, both sex-averaged and sex-specific, along with the physical positions. A summary of the map is presented in Table 1. The physical lengths of the chromosomes are also included. The GENDISCAN map covers 2703.1 Mb, which is 94.3% of the human genome assembly Build 36.2. When we excluded the telomeric heterochromatic regions, which have a wide range of sequencing gaps in especially acrocentric chromosomes (13, 14, 15, 21, 22), the coverage increases to 96.9%.

**Table 1:** Summary of the GENDISCAN genetic map

		Genetic length (cM)			
			
Chromosome	Physical length (Mb)^a^	Sex-averaged	Female	Male	Number of markers
1	237.49	250.06	308.57	174.69	78
2	241.18	244.91	302.42	169.86	77
3	198.27	222.46	265.95	168.87	63
4	189.65	202.05	252.60	138.39	59
5	178.71	195.36	236.40	144.54	63
6	168.57	184.24	226.30	131.55	54
7	156.34	168.64	206.52	118.44	62
8	138.60	149.42	192.26	97.04	53
9	139.63	157.76	183.52	127.62	51
10	134.03	171.84	207.56	128.48	49
11	131.40	145.60	178.38	105.82	46
12	131.30	165.69	196.21	127.66	51
13	93.58	126.13	152.58	94.45	33
14	83.70	109.29	121.54	94.15	44
15	74.73	103.49	124.86	77.28	40
16	86.50	117.80	137.95	94.00	36
17	75.04	123.30	148.84	93.59	43
18	71.35	108.47	130.88	80.07	33
19	61.41	99.17	111.57	85.52	32
20	56.64	88.53	104.83	70.55	31
21	26.18	44.30	55.99	29.47	20
22	28.81	52.08	60.25	41.96	21
Total	2703.11	3230.59	3905.98	2394.00	1039

^a ^Physical length from the first to the last marker of each chromosome of the GENDISCAN map; marker positions are from human genome assembly Build 36.2

We compared our GENDISCAN map with the Rutgers Map v.2, one of the most accurate genetic maps of Caucasians (generated from CEPH and Icelandic families) and including 28121 polymorphic markers, with an average of 301 informative meioses [8]. Among the 1039 markers shown in the GENDISCAN map, we were able to determine the genetic positions of 1006 microsatellite markers common to both the GENDISCAN and Rutgers Map v.2, from Rutgers Map v.2. The genetic positions of the remaining 33 markers, which are not present in Rutgers Map v.2, were estimated by an interpolation considering the physical positions of the markers from human genome assembly Build 36.2.

**Figure 1:**
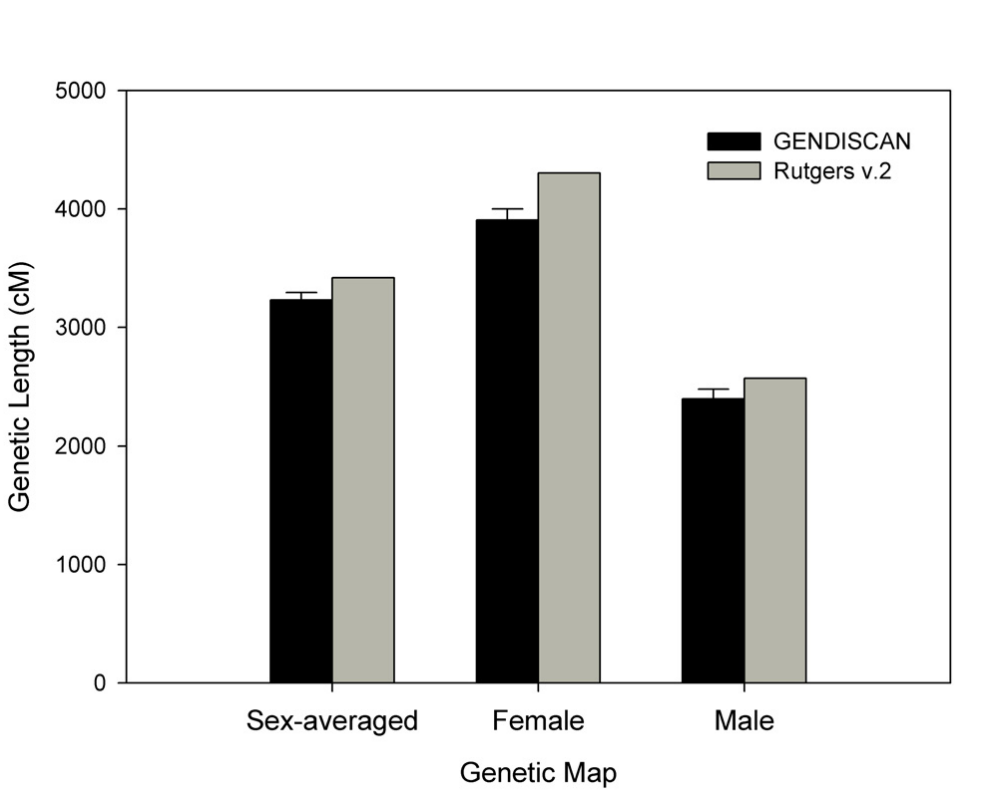
**Comparison of whole genome lengths between the GENDISCAN map and Rutgers Map v.2**. Error bars represents the 95% confidence intervals from the paired t-tests.

**Figure 2:**
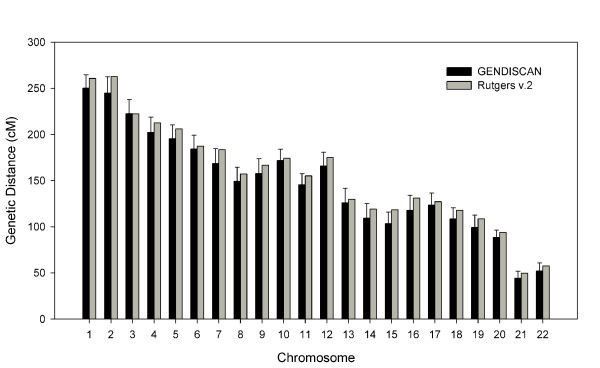
**Comparison of sex-averaged genetic lengths of the chromosomes between the GENDISCAN map and Rutgers Map v.2**. Error bars represents the 95% confidence intervals from the paired t-tests.

**Figure 3:**
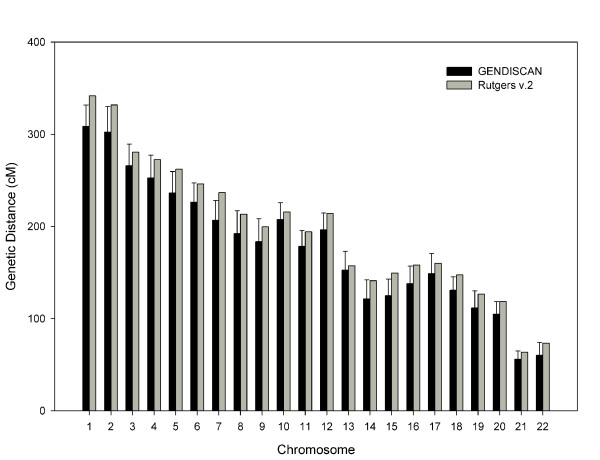
**Comparison of female genetic lengths of the chromosomes between the GENDISCAN map and Rutgers Map v.2**. Error bars represents the 95% confidence intervals from the paired t-tests.

**Figure 4:**
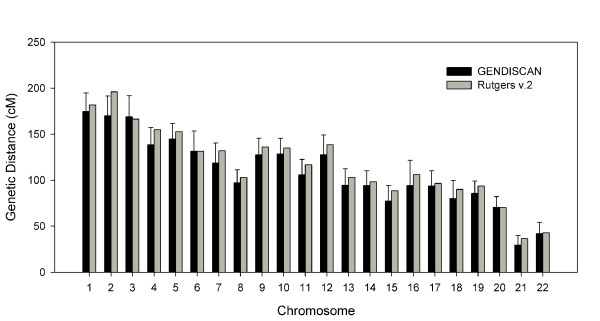
**Comparison of male genetic lengths of the chromosomes between the GENDISCAN map and Rutgers Map v.2**. Error bars represents the 95% confidence intervals from the paired t-tests.

The sex-averaged, female and male whole genome lengths were 3230.6 cM, 3906.0 cM and 2394.0 cM, respectively, which are 5 – 9% shorter than the respective genome lengths of Rutgers Map v.2 (Figure 1). The genetic lengths of chromosomes of the sex-averaged, female and male maps are illustrated in Figures 2, 3 and 4, respectively. All chromosomal lengths of the GENDISCAN map were shorter than those of the Rutgers Map v.2 except for male chromosomes 3 and 6, and sex-averaged chromosome 3. Paired t-tests demonstrated significant differences between the whole genome and chromosome genetic lengths (Table 2). The whole genome and chromosome 2 lengths of all three types of GENDISCAN map were significantly shorter than those of the Rutgers MAP v.2.

**Table 2:** Comparison of genetic map distances of the GENDISCAN map and Rutgers Map v.2

		Sex-averaged map		Female map		Male map	
		
Chr	Number of intervals	Length Difference (cM) ^a^	p-value ^b^	Length Difference (cM) ^a^	p-value ^b^	Length Difference (cM)^a^	p-value ^b^
1	77	-10.64	0.150	-33.12	0.005 **	-7.00	0.491
2	76	-17.80	0.048 *	-29.36	0.037 *	-26.12	0.019 *
3	62	0.14	0.985	-14.51	0.219	2.43	0.832
4	58	-10.56	0.214	-19.84	0.114	-16.48	0.085
5	62	-10.55	0.166	-25.66	0.030 *	-8.16	0.342
6	53	-3.06	0.684	-19.67	0.062	0.16	0.988
7	61	-14.80	0.067	-30.30	0.007 **	-13.42	0.227
8	52	-7.76	0.311	-20.92	0.097	-5.97	0.405
9	50	-9.02	0.259	-16.01	0.200	-8.50	0.341
10	48	-2.35	0.700	-8.24	0.367	-6.35	0.459
11	45	-9.54	0.113	-15.86	0.069	-10.99	0.192
12	50	-9.53	0.209	-17.87	0.056	-10.82	0.316
13	32	-3.73	0.624	-4.73	0.641	-8.46	0.342
14	43	-9.92	0.211	-19.53	0.060	-4.05	0.613
15	39	-14.99	0.019 *	-24.53	0.009 **	-11.23	0.183
16	35	-13.21	0.110	-20.16	0.038 *	-12.14	0.378
17	42	-3.90	0.554	-11.11	0.311	-2.88	0.728
18	32	-9.44	0.120	-16.62	0.024 *	-9.89	0.308
19	31	-9.60	0.157	-15.06	0.107	-8.06	0.233
20	30	-5.52	0.160	-14.03	0.043 *	0.26	0.963
21	19	-5.34	0.158	-7.62	0.089	-6.97	0.168
22	20	-5.57	0.191	-13.09	0.062	-0.89	0.880
Total	1017	-186.69	1 × 10^-8 ^***	-397.84	8 × 10^-17 ^***	-175.53	4 × 10^-5 ^***

^a^Rutgers Map v.2 genetic length subtracted from that of the GENDISCAN map.

^b^p-values estimated from paired t-tests (two-sided).

* p < 0.05,** p < 0.01,***p < 0.001.

Chr; Chromosome

**Figure 5:**
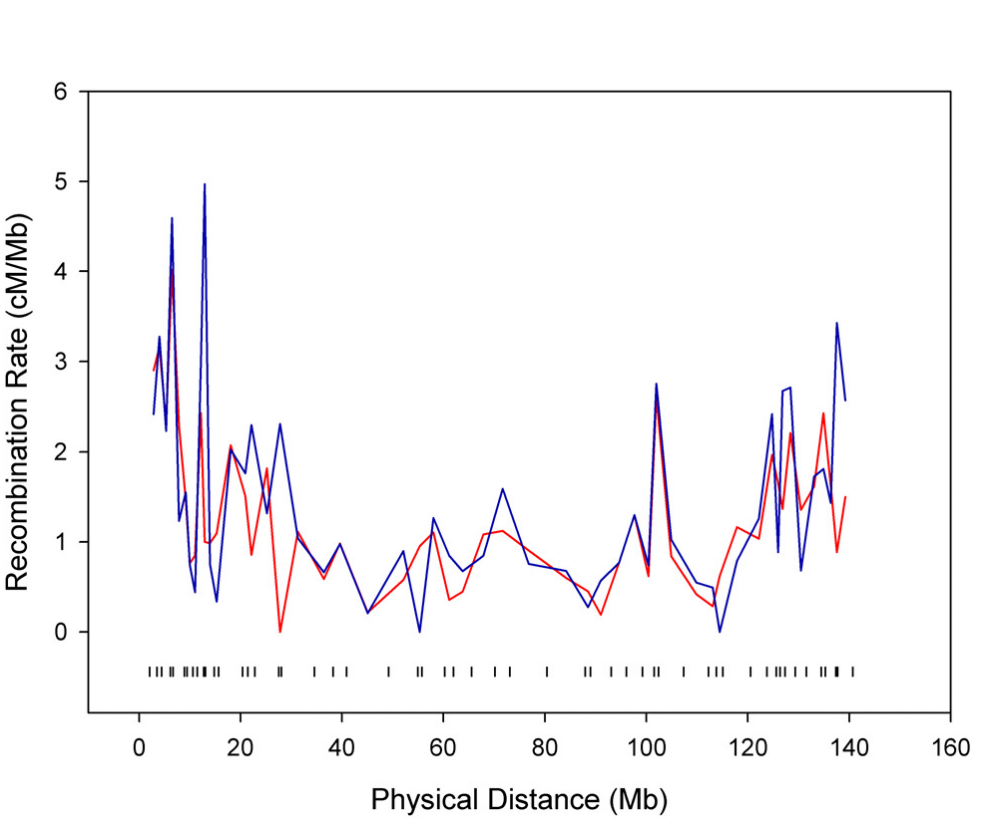
**Comparison of sex-averaged recombination rates of chromosome 8 between the GENDISCAN map (red line) and Rutgers Map v.2 (blue line)**. The physical positions of genetic markers are denoted by black vertical marks.

**Figure 6:**
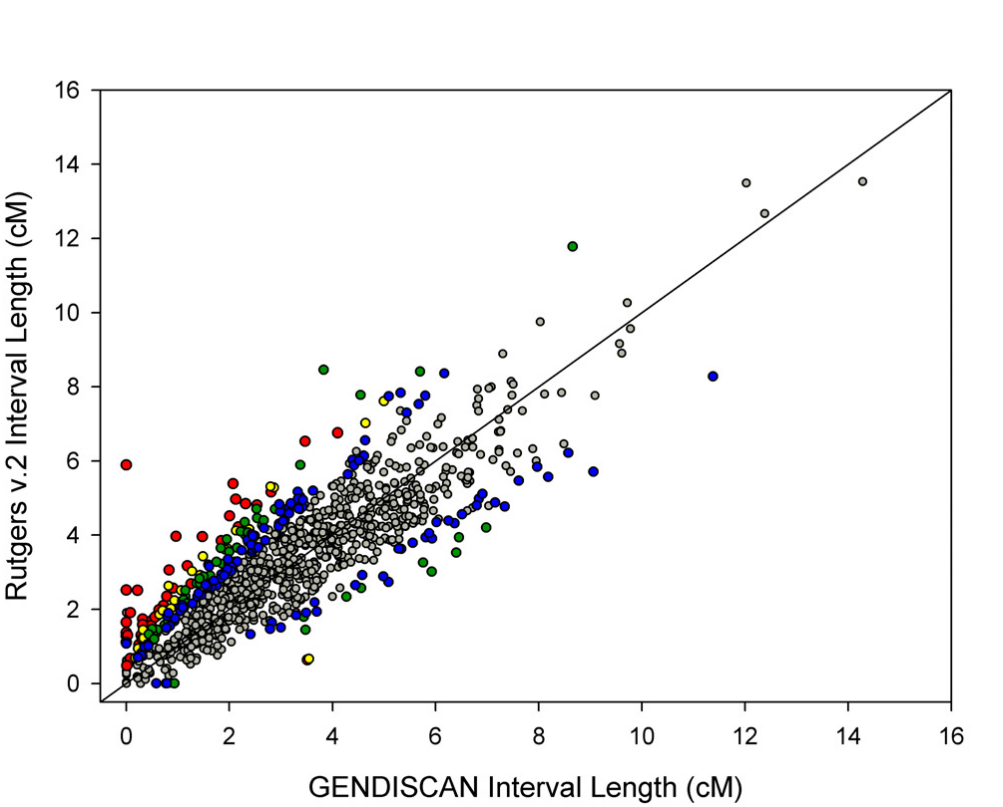
**Comparison of genetic distances of 1017 marker intervals between the GENDISCAN map and Rutgers Map v.2**. Color of dots represents the p-values for significances of genetic length differences between the GENDISCAN map and Rutgers Map v.2. Red, p < 0.0001; yellow, p < 0.001; green, p < 0.01; blue, p < 0.05 and gray, p > 0.05.

**Figure 7:**
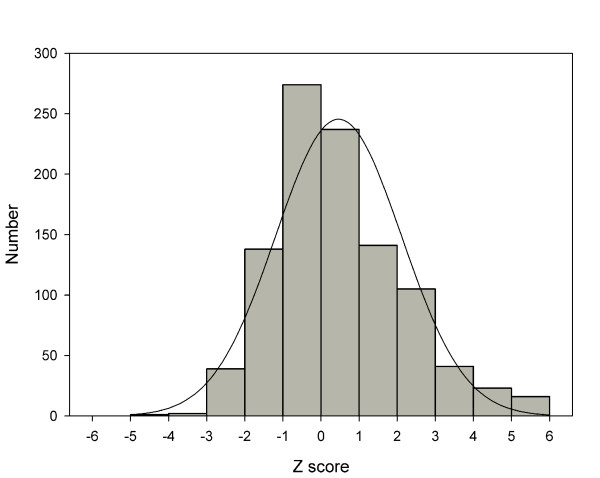
**Distribution of intermarker interval differences between GENDISCAN map and Rutgers Map v.2**. A histogram of 1017 normalized intermarker-interval-differences. The z scores are transformed from the p-values representing the significance of intermarker-interval-differences between the GENDISCAN map and Rutgers Map v.2. A curve representing normal distribution is included for comparison.

The genetic distances between adjacent markers and the 95% confidence intervals were estimated. The average intermarker spacing was 2.66 Mb and 3.17 cM. The intermarker recombination rate was derived by dividing the intermarker genetic distance by the physical distance. The recombination rate patterns for chromosomes are illustrated in additional files (see additional files 2, 3, and 4: recombination rates of the sex-averaged, female and male maps, respectively). These figures are helpful in comparing the genome-wide recombination patterns of GENDISCAN and Rutgers Map v.2. The sex-averaged recombination rate patterns of chromosome 8p were quite different (Figure 5). We compared 1017 intermarker genetic distances between GENDISCAN and Rutgers Map v.2, and calculated p-values for the significance of these differences (Figure 6, see Methods). A histogram of these 1017 normalized intermarker-interval-differences, or z scores transformed from the corresponding p-values, showed that the distribution of intermarker-interval-differences between the GENDISCAN map and Rutgers Map v.2 was close to normal (Figure 7).

Although most of the intervals of the GENDISCAN map and Rutgers Map v.2 were in good agreement, 40 intervals (3.9%) differed significantly after Bonferroni's multiple comparison correction (p-value < 4.9 × 10^-5^). Two of these 40 intervals were excluded, since their intermarker genetic distances on Rutgers Map v.2 were derived by interpolation. Thus, we identified 38 ethnically different marker intervals (Table 3). The differences in local genomic structure difference in these intervals may cause local recombination rate differences among ethnic groups.

**Table 3:** List of marker intervals that differed significantly (two-sided p < 4.9 × 10^-5^) between the GENDISCAN and Rutgers v.2 sex averaged maps

Cytogenetic Position	Marker Interval	GENDISCAN Interval Length	Rutgers v.2 Interval Length	P-value
1p34.3	D1S255-D1S186	0.47	1.4	6 × 10^-6^
2p25.3	D2S323-D2S319	0.83	3.06	9 × 10^-8^
2p16.1	D2S1364-D2S370	0.59	1.82	2 × 10^-6^
2p14-p13.2	D2S2152-D2S2110	2.12	4.97	7 × 10^-8^
2q33.3	D2S2358-D2S2321	0	1.65	8 × 10^-10^
2q37.2	D2S2973-D2S2202	0.63	1.99	3 × 10^-5^
3q29	D3S240-D3S1265	0	2.52	0
4q33-q34.1	D4S2910-D4S1539	0.97	3.97	2 × 10^-14^
5q14.3-q15	D5S1725-D5S2498	1.25	2.69	2 × 10^-5^
5q21.1	D5S1503-D5S409	0.22	2.51	0
5q22.1	D5S2501-D5S2027	0.56	1.79	6 × 10^-6^
6p21.31-p21.1	D6S1576-D6S1575	4.10	6.76	2 × 10^-5^
6q21	D6S416-D6S1603	0.03	1.30	0
8p22	D8S552-D8S1790	0.32	1.59	2 × 10^-8^
8p21.3	D8S1116-D8S1734	1.19	3.17	6 × 10^-6^
8q21.3	D8S273-D8S270	0.79	2.35	2 × 10^-6^
8q24.23	D8S1783-D8S274	0.31	1.20	2 × 10^-5^
9q22.2-q22.31	D9S283-D9S1781	2.17	4.22	9 × 10^-6^
9q31.2	D9S1162-D9S261	0.08	1.91	0
10p15.3	D10S249-D10S602	2.07	5.39	1 × 10^-6^
11q14.2	D11S1887-D11S1780	0.50	1.71	2 × 10^-6^
12q23.3	D12S1636-D12S1683	0.07	0.67	4 × 10^-7^
13q33.3-q34	D13S778-D13S1315	2.80	5.16	2 × 10^-5^
13q34	D13S261-D13S285	0.91	2.57	9 × 10^-8^
14q11.2	D14S283-D14S990	3.51	0.64	1 × 10^-5^
14q12-q13.1	D14S1071-D14S741	3.47	6.53	1 × 10^-6^
15q12-q13.1	D15S156-D15S1019	2.00	4.52	1 × 10^-6^
15q15.1	D15S146-D15S214	0.01	0.48	0
15q26.2	D15S1014-D15S212	0.48	1.56	5 × 10^-6^
16p13.3	D16S521-D16S3395	0	5.89	0
16p13.2	D16S3087-D16S404	0.25	1.05	2 × 10^-6^
17p13.2	D17S1854-D17S1832	0.72	2.10	1 × 10^-5^
17q25.1-q25.3	D17S2192-D17S802	2.54	4.81	8 × 10^-6^
18p11.31	D18S1132-D18S452	1.48	3.96	1 × 10^-6^
18q21.33	D18S1147-D18S68	1.84	3.85	7 × 10^-6^
19q13.3	D19S591-D19S424	0.29	1.30	3 × 10^-8^
21q21.1	D21S1902-D21S1884	0.32	1.74	3 × 10^-13^
21q21.2	D21S272-D21S1914	0	1.37	3 × 10^-14^

## Discussion

The human genome varies among ethnic groups as a result of their diverse history. Complex phenotypes result from the interaction of different genes with the unique environments to which humans are exposed. Finding specific disease loci within families helps identify the genetic causes of complex diseases effectively.

The GENDISCAN study, which began in 2003, was designed to identify specific genetic loci and genes that influence complex traits and diseases in Northeast Asian populations. As the lifestyle of Northeast Asians has become more westernized, the prevalence of complex diseases, such as diabetes, obesity, cardiovascular diseases and cancers, has increased. Linkage analyses require appropriate genetic maps for identifying correct loci. Hence we constructed a genetic map of an Asian population as an initial step in our GENDISCAN study.

World populations can be grouped into nine clusters based on genetic distances: African; New Guinean and Australian; Pacific Islander; Southeast Asian; Northeast Asian; Arctic Northeast Asian; Amerind; North African and West Asian; and European [15,16]. The Northeast Asian cluster includes Japanese, North Chinese, Koreans and Mongolians. Since genetic distance within clusters is closer than between clusters, our genetic map of Mongolians may be more applicable to Japanese, North Chinese and Koreans than genetic maps of Caucasians.

When we compared the GENDISCAN map with the Caucasian Rutgers Map v.2, we found that the genetic length of the GENDISCAN map was much shorter. Genome-wide, Mongolians show about 1.9 fewer recombinations per meiosis compared with Caucasians. This is due to a general trend of 1017 marker intervals overall rather than several specific genomic regions. Although the genome-wide recombination rate patterns showed good agreement between the two ethnic groups, those of Asians were generally smaller. However, we also identified several regions in which the patterns did not correlate; that is some recombination jungles in the GENDISCAN map appear as recombination deserts in Rutgers Map v.2, and *vice versa*.

A previous examination of ethnic differences in genetic maps identified no significant differences for genome-wide genetic length between Caucasians and Asians, but found a significant local difference on 8p, a finding identical to ours [11]. The ethnic difference on 8p is likely due to a frequent local polymorphic inversion [11]. Interestingly, we found a suggestive inversion of marker orders against the physical map on 8p22 (D8S520 and D8S1759 with likelihood of [inversion]/[original] = 17.8) by *FLIPS *option of CRIMAP. The sex averaged map of chromosome 8, reflecting the inversion, is available on additional file 5. Since previous ethnic-specific maps were constructed using a small number of genetic markers (n = 353) and nuclear families, with most families made up of no grandparents and several children, these findings may be less robust than ours [11]. Generally, determining the phase of genotypes to find recombinations requires genetic information of three generations, or that of two generations with many children.

Recombination is related to diversity of DNA sequences, linkage disequilibrium (LD) and copy number changes [17]. Asian-Americans have a smaller number of single nucleotide polymorphisms (SNPs) than European-Americans (5050 versus 6736) and lower minor allele frequencies (MAFs) of the SNPs (820 (16%) versus 1579 (23%) whose MAFs > 5%) [18]. Moreover, Asians have a smaller number of haplotypes per block than Caucasians (3.5 versus 4.2) [19] and Asian Americans have fewer copy number variations (CNVs) than European Americans (14.8 versus 16.3) [20]. These findings indicate that Asians have a more homogeneous genome than Caucasians, probably as a result of their low recombination rate.

Although genetic studies using SNP markers or resequencing on random populations rather than families give fine-scale recombination pattern data, these data are indirect and may be biased by mutation, selection, drift and demography [21]. Most recombinations occur in short kilobase scale regions, known as recombination hotspots [22]. Fine-scale data have suggested that these hotspots and intensities may differ among different ethnic groups [23]. Moreover, population-specific hotspots have also been identified, and populations of close geographic regions tend to show similar hotspot intensities [24]. The properties of recombination hotspots are not well known, but some characteristics have been described [22]. The local DNA sequences of recombination hotspots present more long terminal repeats of retrotransposons, THE1A and THE1B, as well as CT-rich and GA-rich repeats [22]. Some DNA motifs, such as the CCTCCCT oligomer of THE1A and THE1B, and the CCCCACCCC oligomer within recombination hotspots, may be local DNA signals of recombination hotspots [22]. Additional studies of recombination patterns, with comparisons among different ethnic groups, are necessary to understand the nature of recombination hotspots. The present study, which found significantly different marker intervals between two ethnic groups, can facilitate further comparisons of genomic variations among ethnic groups.

Some comparative characteristics of the GENDISCAN map and Rutgers Map v.2 deserve attention. The number of markers used for genetic mapping was quite different (1039 for GENDISCAN map, 23389 for autosomes of Rutgers Map v.2). This may have caused the difference in genetic distances, since high, but not low, density markers can identify two very close individual recombination events. These double recombination events, however, were eliminated when constructing both maps, since they were thought likely to be non-Mendelian genotyping errors, not two individual recombinations. Compared with Rutgers Map v.1, although the number of markers used in Rutgers Map v.2 was increased nearly twofold [7,8], the marker-matched genome-wide genetic length of Rutgers Map v.2 is 2 cM shorter than that of Rutgers Map v.1 (data not shown). Moreover, construction of the GENDISCAN genetic map of chromosome 1 using fewer and fewer markers increases, rather than decreases, the genetic length (data not shown). Biologically, the double recombinations are considered very rare in human meiosis, since not only is recombination uncommon (about 32 per genome per meiosis), but also one chiasma inhibits formation of another chiasma nearby (positive interference). The Kosambi map function, which is widely used and thought to reflect adequate levels of double recombination in humans, has been applied for calibrating the slight possibility of double recombination when constructing genetic maps [25]. Therefore, if non-Mendelian genotyping errors, which appear as double recombinations, were properly eliminated during the cleaning process, there is no reason to expect that small numbers of markers reduce genetic length in genetic maps.

The statistical methods used in the comparisons are also worthy of note. The paired t-test, which is used to compare of genetic lengths of the whole genome and chromosomes, is not the method of choice for testing the difference between sums of intervals (genetic lengths of the whole genome and chromosomes), but is the method of choice for testing the difference between the average of intervals. However, since each interval distance of the GENDISCAN map is significantly shorter than that of Rutgers Map v.2, the sum of interval distances of GENDISCAN is likely shorter. We therefore used paired t-tests to compare of whole genome genetic lengths and to estimate the significance levels of their differences.

We assumed that the number of recombination events between markers would follow a binomial distribution, then a normal distribution for estimating the 95% confidence interval of each intermarker genetic distance (see Methods, Statistical Methods for details). Adjusted Wald methods were used for marker intervals, whose θ equals zero. The 95% confidence intervals of the GENDISCAN interval distances and p-values for the significance of differences between GENDISCAN and Rutgers Map v.2 intervals must be interpreted with caution, since they were calculated under those assumptions. However, we believe that those values are helpful parameters for assessing the certainty and finding the significant differences between genetic maps.

## Conclusion

In summary, we constructed a genetic map with large Asian families. The GENDISCAN map may provide better results than Caucasian genetic maps in linkage analysis of Asians. We also found that the GENDISCAN map shows shorter genetic distances than a Caucasian genetic map, with Asians having 1.9 fewer recombination events per meiosis than Caucasians. The recombination rates of some marker intervals differed significantly between populations. Our results illustrate the differences in recombination patterns between ethnic groups and provide clues to their underlying genomic variations.

## Methods

### Subjects

Genetic mapping was performed as part of the GENDISCAN study, designed for linkage analysis of a number of complex traits of the Asian population. In 2006, we collected and genotyped 978 individuals in Dashbalbar, Dornod Province, Mongolia. Their relationships were determined from interviews and confirmed by genotype data (see Methods, Genotyping). Informed consent was obtained from all enrolled subjects, and the study protocol was approved by the institutional review board (IRB) of Seoul National University (approval number, H-0307-105-002).

The pedigree size was exceedingly large for an effective analysis; it included seven families, with the largest including 949 genotyped subjects. Hence, we separated the seven families into 73 families, causing 44 genotyped subjects to be included in more than one family. However, no meioses overlapped in this procedure. The final pedigree included 1446 individuals, with 1026 subjects genotyped, and a total of 1790 meioses, with 895 for each gender, and 1500 meioses available for investigation.

### Genotyping

Venous blood was collected and DNA was extracted from leukocytes using standard protocols. Genotyping was completed with 1039 microsatellite markers throughout the autosomes by deCODE genetics.

Genotyping errors were detected and removed using three software packages: nonpaternity was checked with PREST [26]; individual relationships other than paternity were identified and corrected with PEDCHECK [27]; and non-Mendelian errors were investigated with SimWalk [28]. Mendelian and non-Mendelian errors constituted 0.13% and 0.26%, respectively, of all genotype data.

### Genetic Mapping

After correcting genotype errors, the GENDISCAN map was generated with CRIMAP software [14]. The orders of markers were determined by comparison with the physical map of the human genome assembly Build 36.2. We confirmed the order of markers with the *FLIPS *option. The *FIXED *option of CRIMAP was used to calculate recombination fractions between two successive markers. The Kosambi map function was used for the estimation of genetic distances. Genetic maps for both the sex specific and sex averaged genetic maps were generated.

### Statistical Methods

The genetic lengths of the whole genome and each chromosome were compared between the GENDISCAN and Rutgers Map v.2 using paired t-tests. For these comparisons, all 1017 intermarker intervals contributed to genome-wide genetic distance estimation and the marker intervals on the particular chromosome to the genetic distance of each chromosome.

To estimate the 95% confidence interval of each intermarker genetic distance of the GENDISCAN map, we assumed that the number of recombination events between adjacent markers would follow a binomial distribution (*Nr *~ B(*Nm*, *θ*): *Nr*, the number of recombination; *Nm*, the number of informative meioses; *θ*, recombination fraction). Moreover, such a distribution was transformed into a normal distribution, since the number of meioses between markers was not small (n > 30). We obtained *θ *and *Nm *of each intermarker interval from the CRIMAP software [14], which calculates recombination fractions through both two-point and multipoint maximum likelihood estimation. For example, if M1, M2, M3 and M4 are genetic markers with the correct order in a pedigree, the CRIMAP software estimates *θ *between M2 and M3, even if the interval (M2, M3) is not informative, using the flanking informative markers (M1 and M4). Therefore, the *Nm *obtained above is not the direct value for calculating the final recombination fraction, but was used to understand approximately how large the *Nm *was that was used for estimating *θ*.

The final estimates of *θ *and *Nm *for each interval were used to calculate the 95% confidence intervals of recombination fractions using the following equations:

*θ *± 1.959·S_*θ*_/√*Nm*, where S_*θ*_^2 ^= *θ*·(1 - *θ*),

which are from the normal distribution and binomial distribution, respectively. In the case where the estimated *θ *equals 0, we applied the adjusted Wald method, in which *θ *is replaced by *θ*' = 2/(*Nm *+ 4) [8]. In this study, 25 of the 1017 intermarker recombination fractions were calculated by adjusted Wald methods. Finally, the 1017 recombination fractions and the confidence intervals were transformed into genetic distances (cM) by the Kosambi map function. In addition, the statistical significance levels of differences in *θ*s between GENDISCAN and Rutgers Map v.2 were tested based on the confidence intervals. Bonferroni's multiple comparison correction method was applied to determine significant differences (p-value < 4.9 × 10^-5^).

## Authors' contributions

YSJ designed the study, carried out the genetic mapping, performed statistical analyses, and drafted the manuscript. HP participated in the survey and genotyping and helped to draft the manuscript. MKL managed the pedigree and genotype data of subjects. JIK participated in the design and coordination of the study. JS and SIC helped to perform the statistical analyses and to draft the manuscript. JSS participated in the study design and coordination and helped to draft the manuscript. JIK, JS, SIC, and JSS conceived of the whole GENDISCAN study. All authors read and approved the final manuscript.

## Supplementary Material

Additional file 1 **Details of the GENDISCAN map.** A file providing details of the GENDISCAN map (*e.g*., the genetic/physical positions, the genetic distance of intervals, the 95% confidence intervals of the genetic distance of intervals, the genetic distances of Rutgers Map v.2 intervals, the p-values denoting the significance levels for differences in the genetic distance between the GENDISCAN map and Rutgers Map v.2 intervals, the heterozygosity of markers, the number of informative meioses of markers and the number of informative meioses of between markers). Click here for file

Additional file 2 **Recombination rates of sex-averaged map.** Figures of recombination rate patterns of all autosomes of the GENDISCAN and Rutgers v.2 sex-averaged maps. Click here for file

Additional file 3 **Recombination rates of female map.** Figures of recombination rate patterns of all autosomes of the GENDISCAN and Rutgers v.2 female maps. Click here for file

Additional file 4 **Recombination rates of male map.** Figures of recombination rate patterns of all autosomes of the GENDISCAN and Rutgers v.2 male maps. Click here for file

Additional file 5 **The sex averaged map of chromosome 8 with the inversion (D8S520 and D8S1759).** We found a suggestive inversion on 8p22. This additional file shows the genetic map of chromosome 8, which reflects the inversion of genetic marker order on 8p22 (D8S520 and D8S1759). The genetic length of chromosome 8 decreased more than the original map. Click here for file
