# Supplementary material for: A genome-wide Asian genetic map and ethnic comparison: The GENDISCAN study
Source: BMC Genomics. 2008 Nov 25;9:554. doi: 10.1186/1471-2164-9-554 (PMC2612022; doi:10.1186/1471-2164-9-554)
Supplement: Additional file 2 — Recombination rates of sex-averaged map. Figures of recombination rate patterns of all autosomes of the GENDISCAN and Rutgers v.2 sex-averaged maps. [file 1471-2164-9-554-S2.pdf]

Chromosome 1

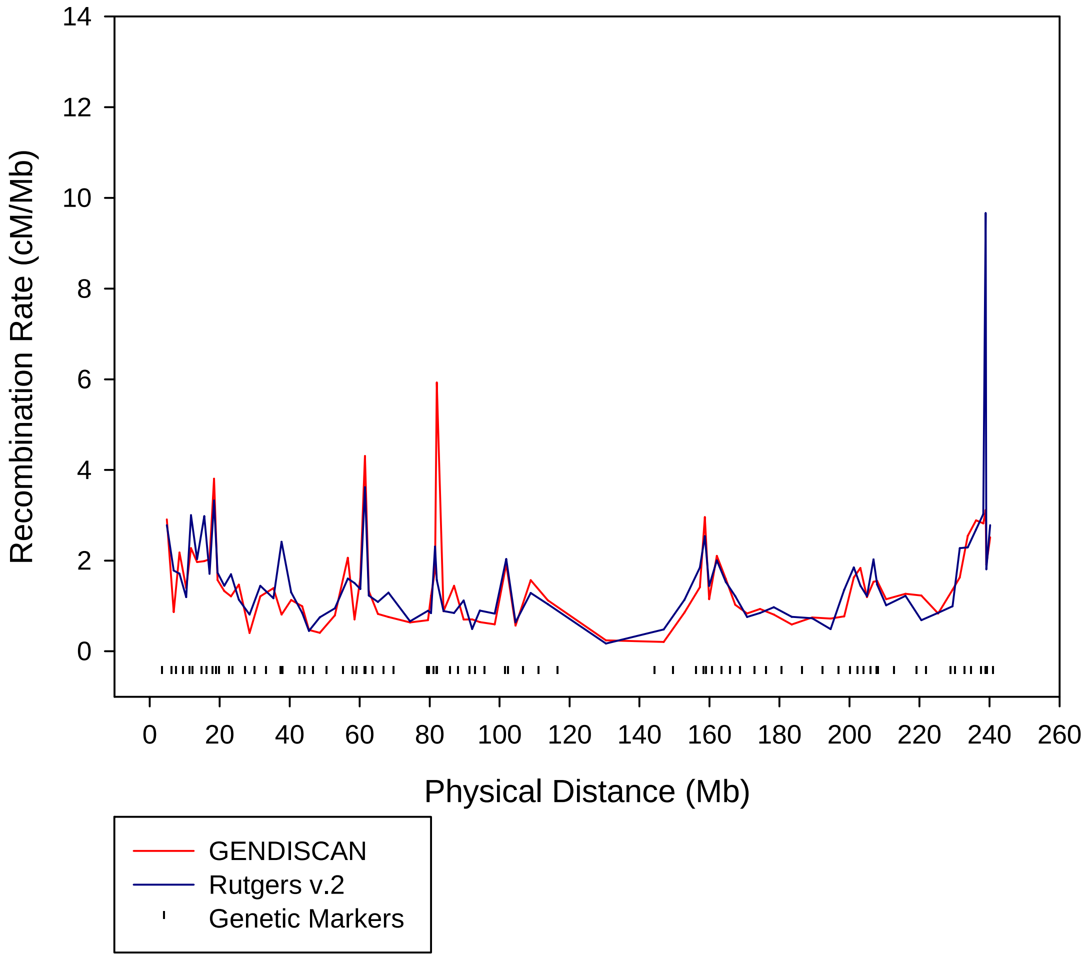

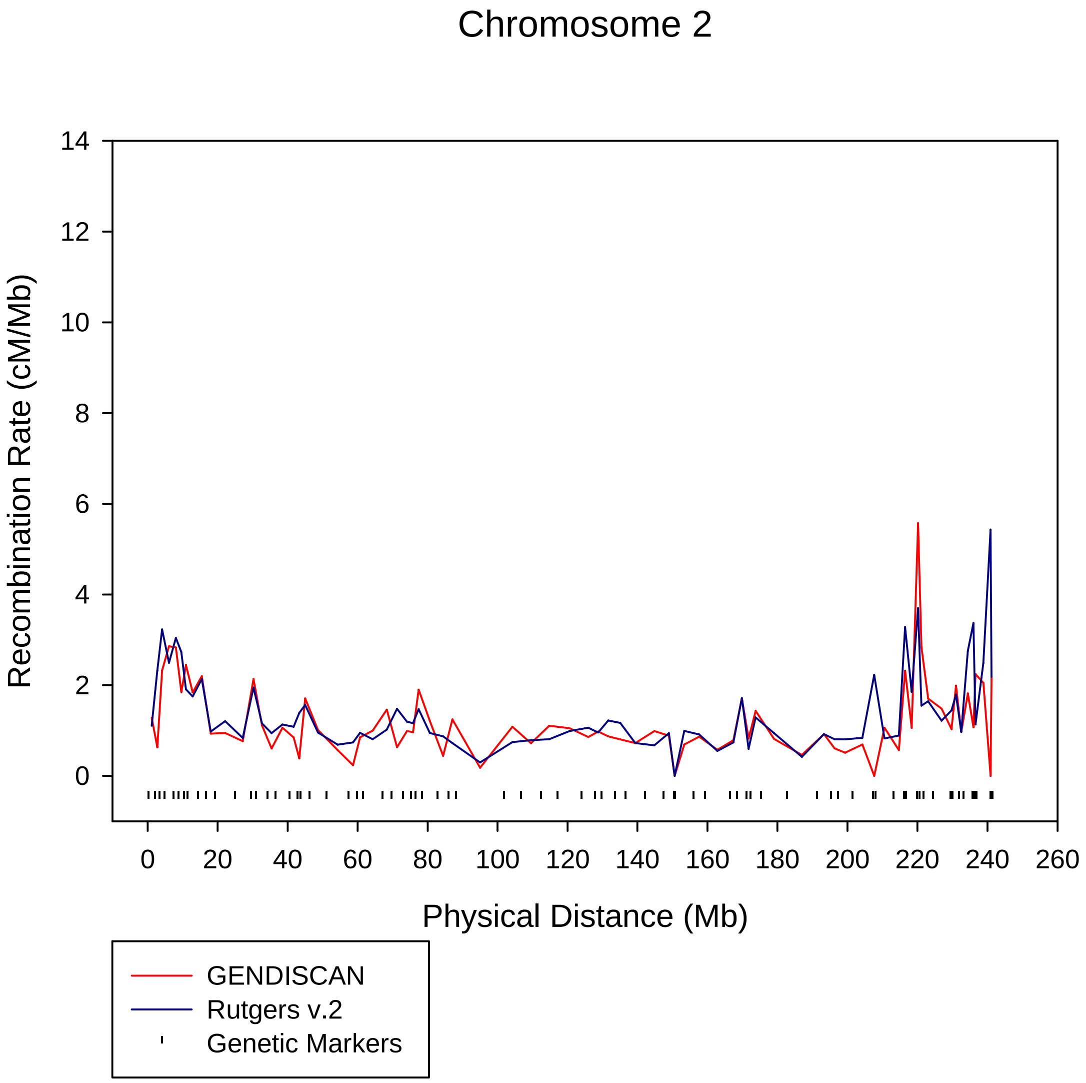
Chromosome 2
Recombination Rate (cM/Mb)
0
2
4
6
8
10
12
14
0
20
40
60
80
100
120
140
160
180
200
220
240
260
Physical Distance (Mb)
GENDISCAN
Rutgers v.2
Genetic Markers

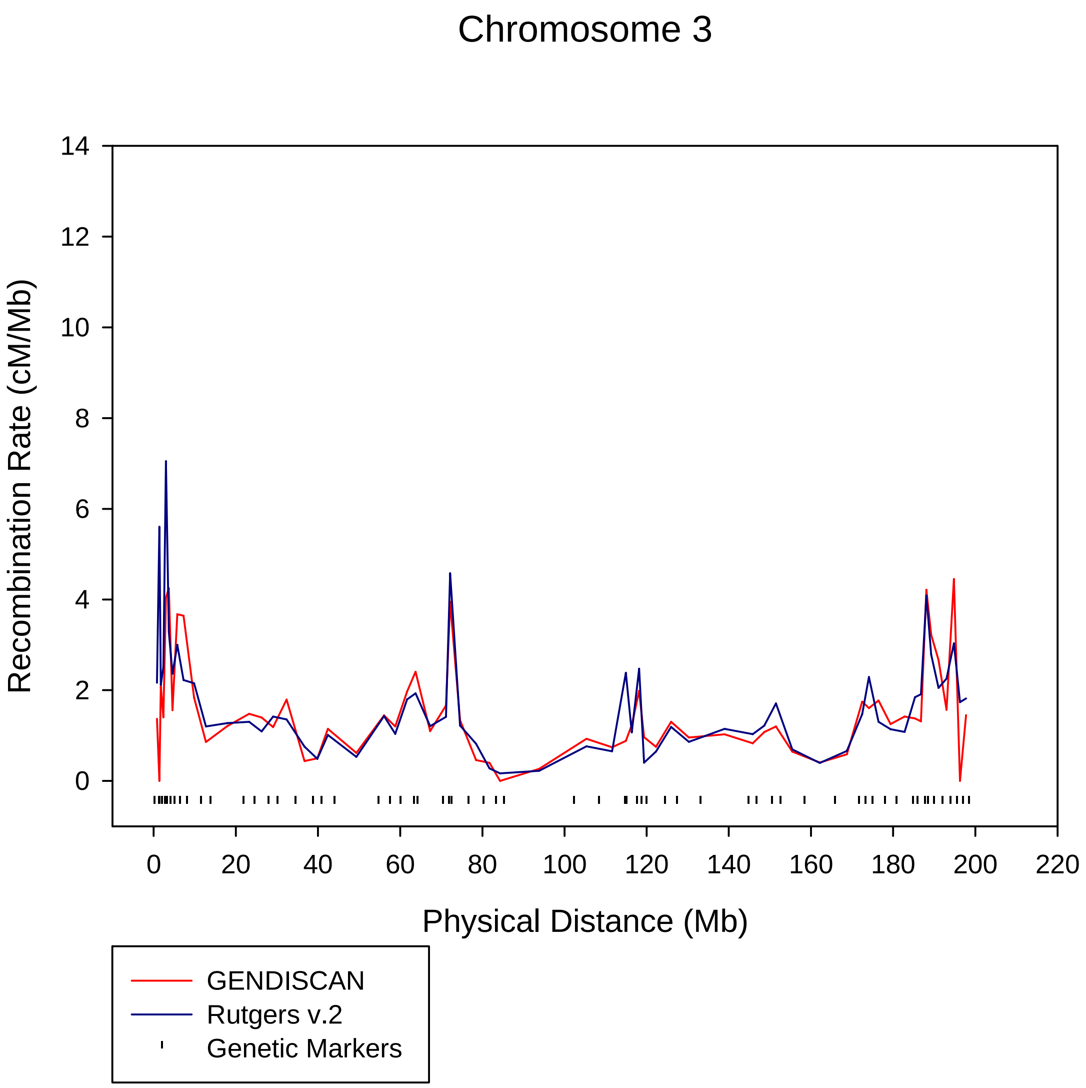

Chromosome 3
Recombination Rate (cM/Mb)
0
2
4
6
8
10
12
14
0
20
40
60
80
100
120
140
160
180
200
220
Physical Distance (Mb)
GENDISCAN
Rutgers v.2
Genetic Markers

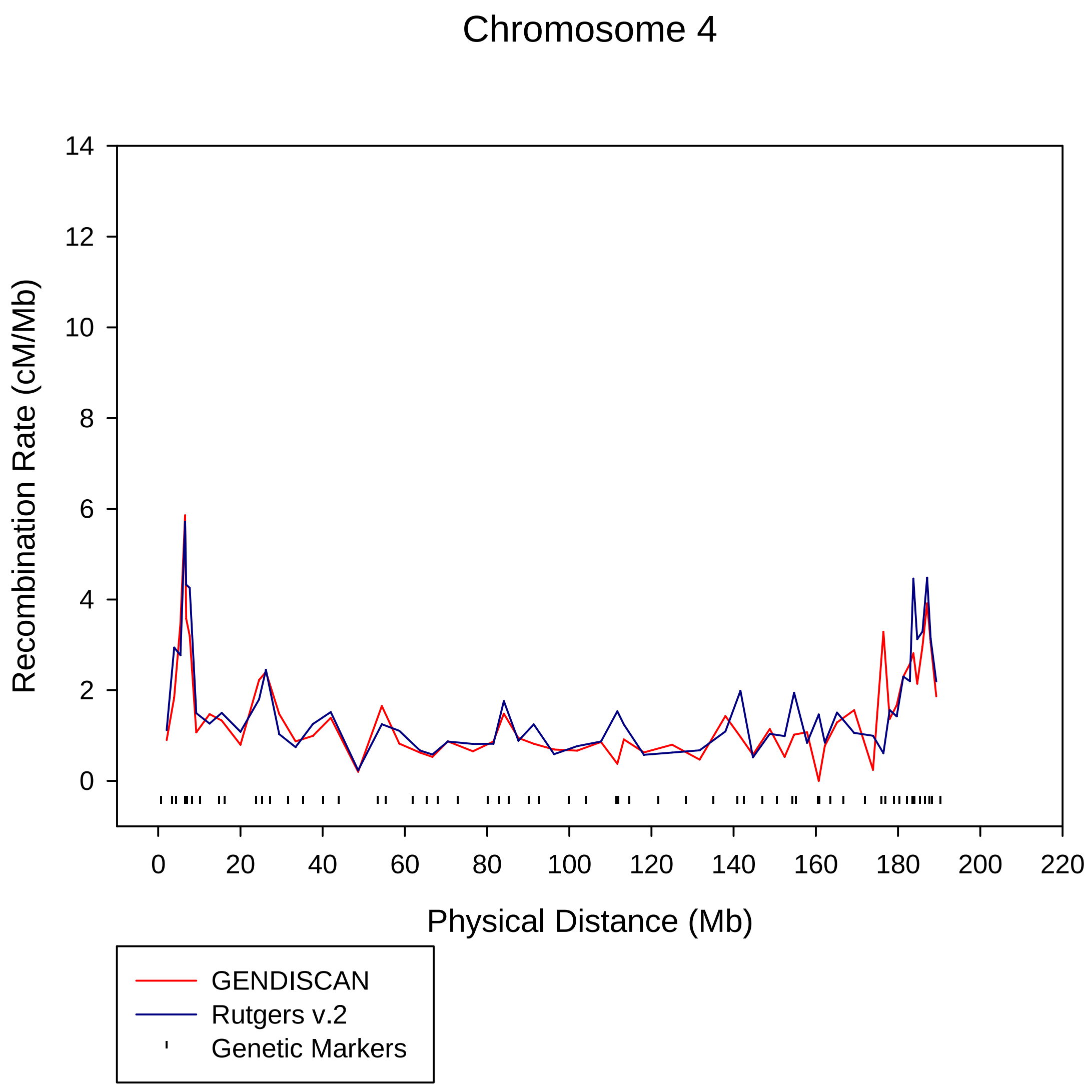
Chromosome 4
Recombination Rate (cM/Mb)
0
2
4
6
8
10
12
14
Physical Distance (Mb)
0
20
40
60
80
100
120
140
160
180
200
220
GENDISCAN
Rutgers v.2
Genetic Markers

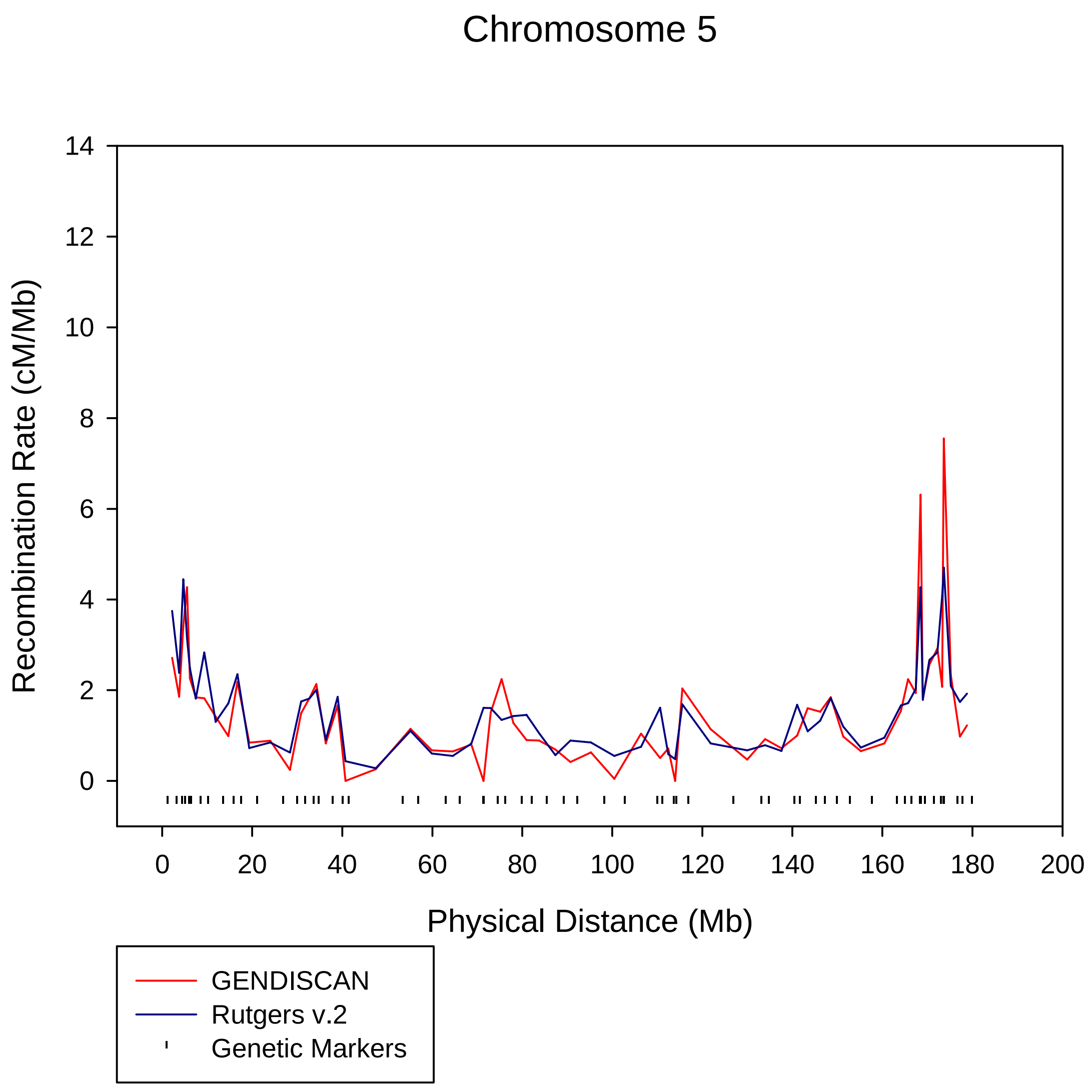

Chromosome 5
Recombination Rate (cM/Mb)
0
2
4
6
8
10
12
14
0
20
40
60
80
100
120
140
160
180
200
Physical Distance (Mb)
GENDISCAN
Rutgers v.2
Genetic Markers

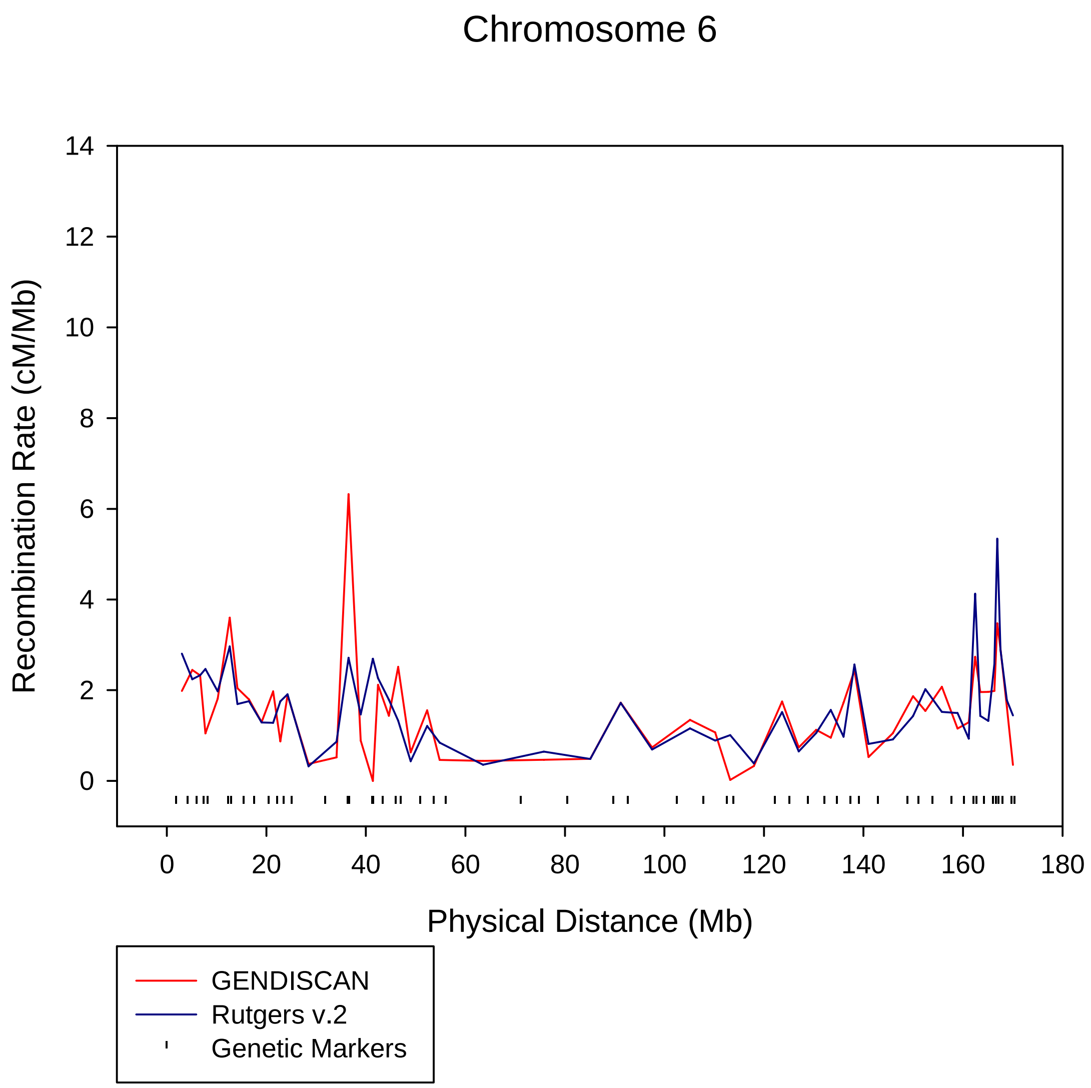

Chromosome 6
Recombination Rate (cM/Mb)
14
12
10
8
6
4
2
0
0
20
40
60
80
100
120
140
160
180
Physical Distance (Mb)
GENDISCAN
Rutgers v.2
Genetic Markers

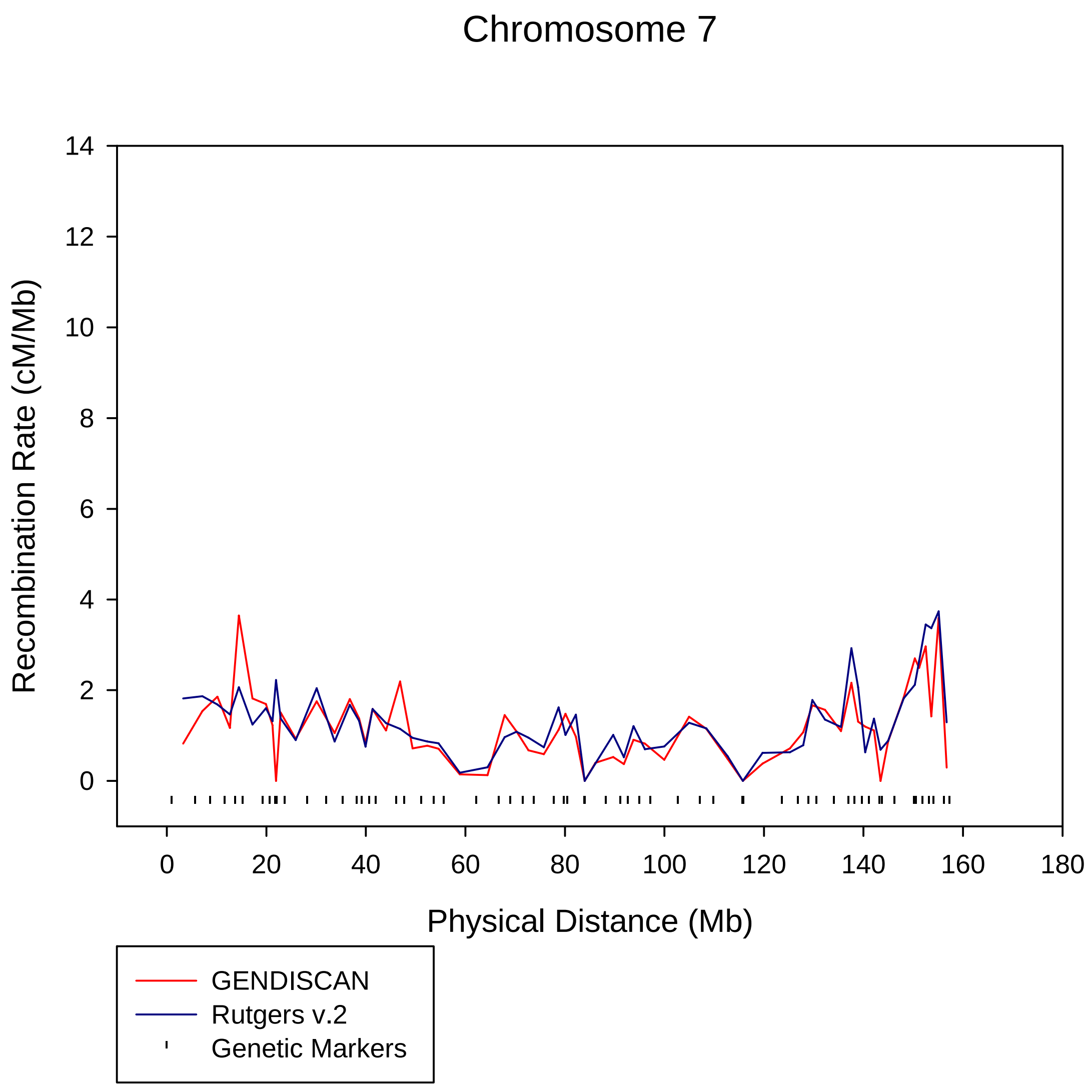

Chromosome 7
Recombination Rate (cM/Mb)
0
2
4
6
8
10
12
14
0
20
40
60
80
100
120
140
160
180
Physical Distance (Mb)
GENDISCAN
Rutgers v.2
Genetic Markers

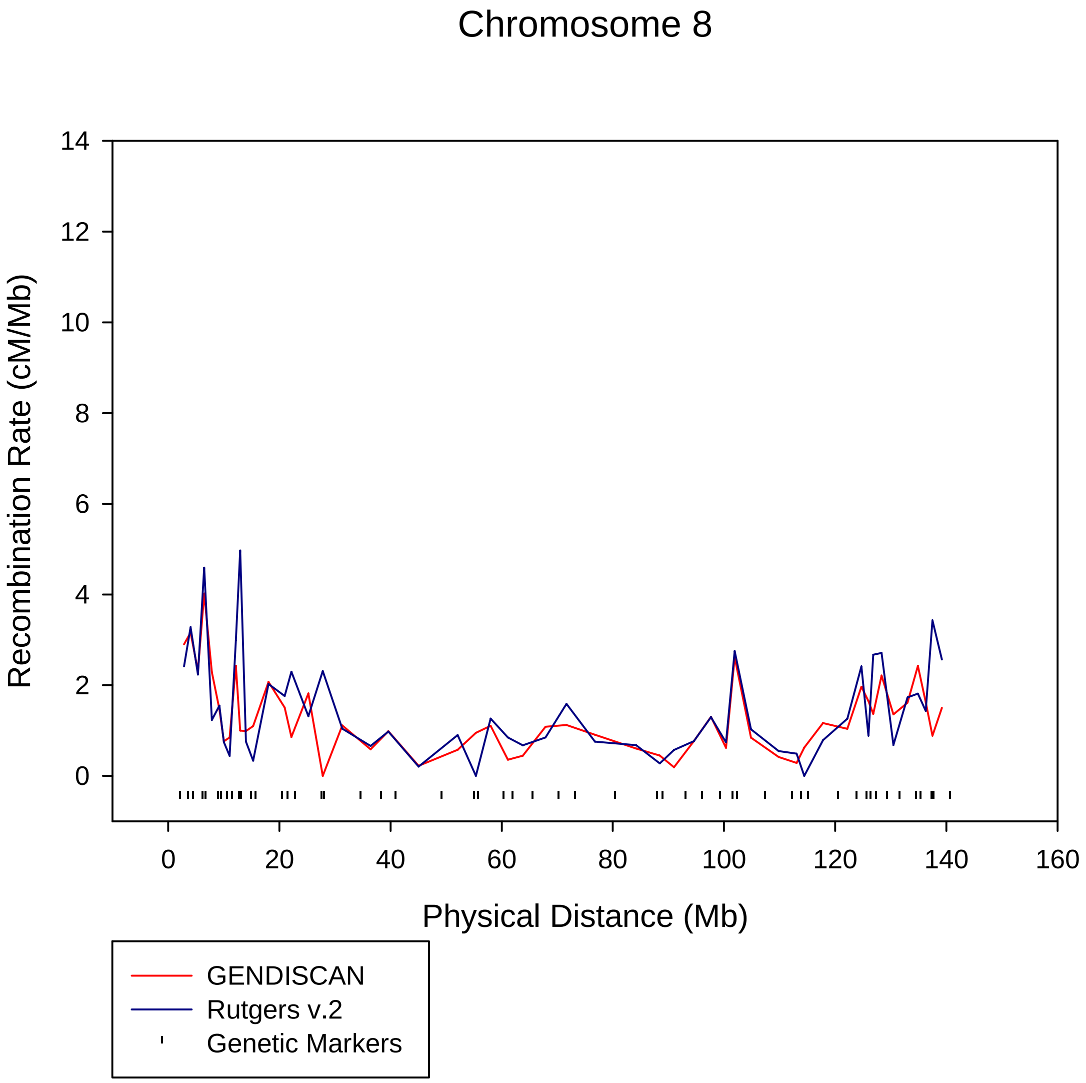

Chromosome 8
Recombination Rate (cM/Mb)
14
12
10
8
6
4
2
0
0
20
40
60
80
100
120
140
160
Physical Distance (Mb)
GENDISCAN
Rutgers v.2
Genetic Markers

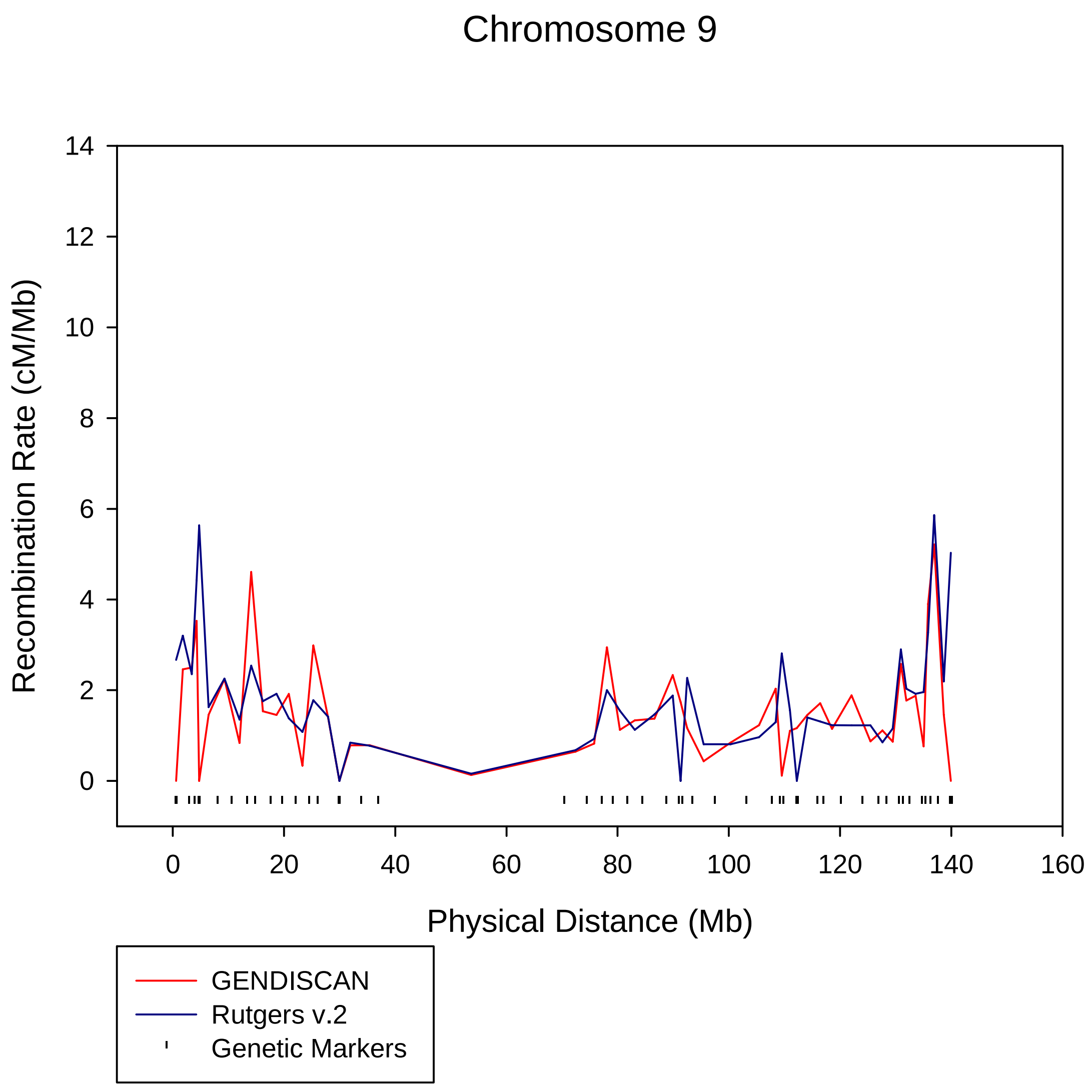

Chromosome 9
Recombination Rate (cM/Mb)
14
12
10
8
6
4
2
0
0
20
40
60
80
100
120
140
160
Physical Distance (Mb)
GENDISCAN
Rutgers v.2
Genetic Markers

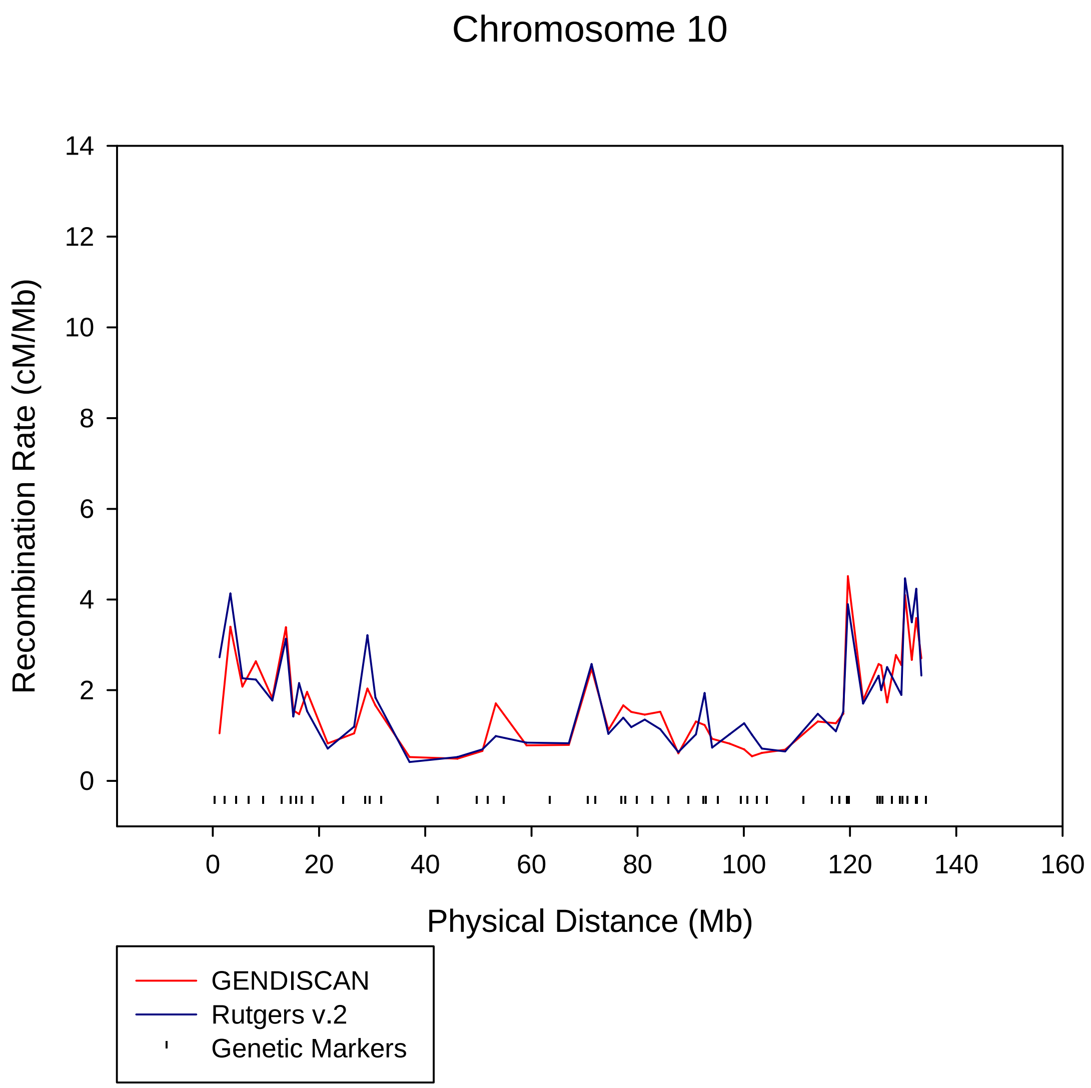
Chromosome 10
Recombination Rate (cM/Mb)
0
2
4
6
8
10
12
14
0
20
40
60
80
100
120
140
160
Physical Distance (Mb)
GENDISCAN
Rutgers v.2
Genetic Markers

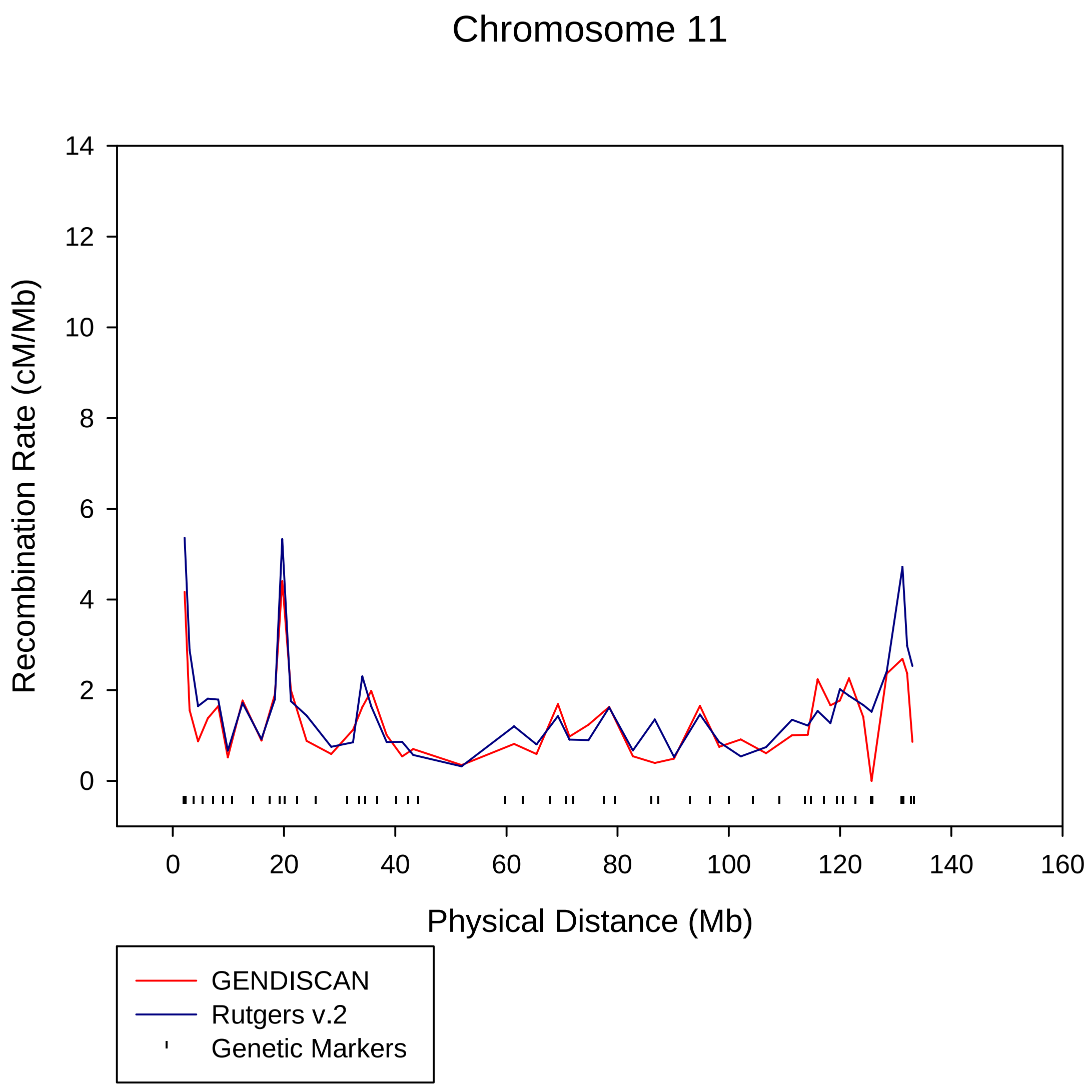
Chromosome 11
Recombination Rate (cM/Mb)
14
12
10
8
6
4
2
0
0
20
40
60
80
100
120
140
160
Physical Distance (Mb)
GENDISCAN
Rutgers v.2
Genetic Markers

Chromosome 12

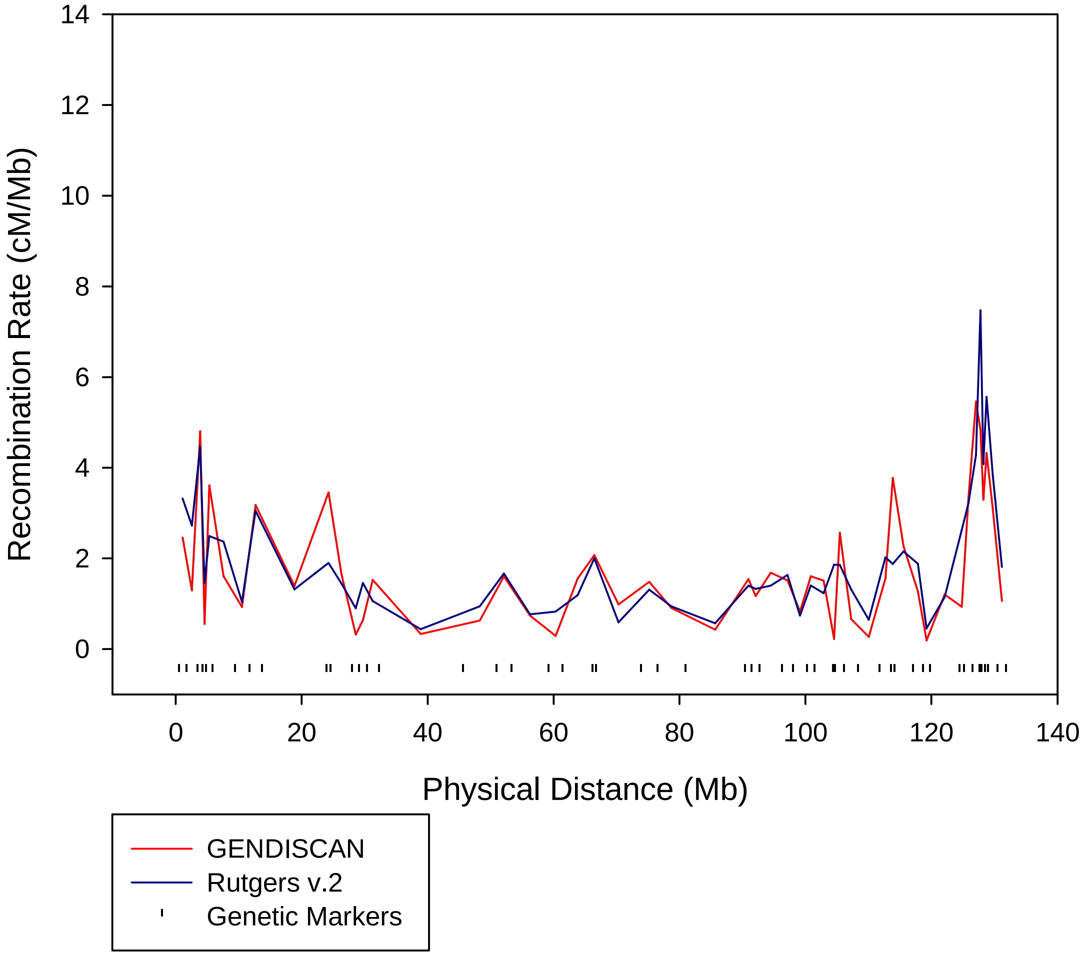

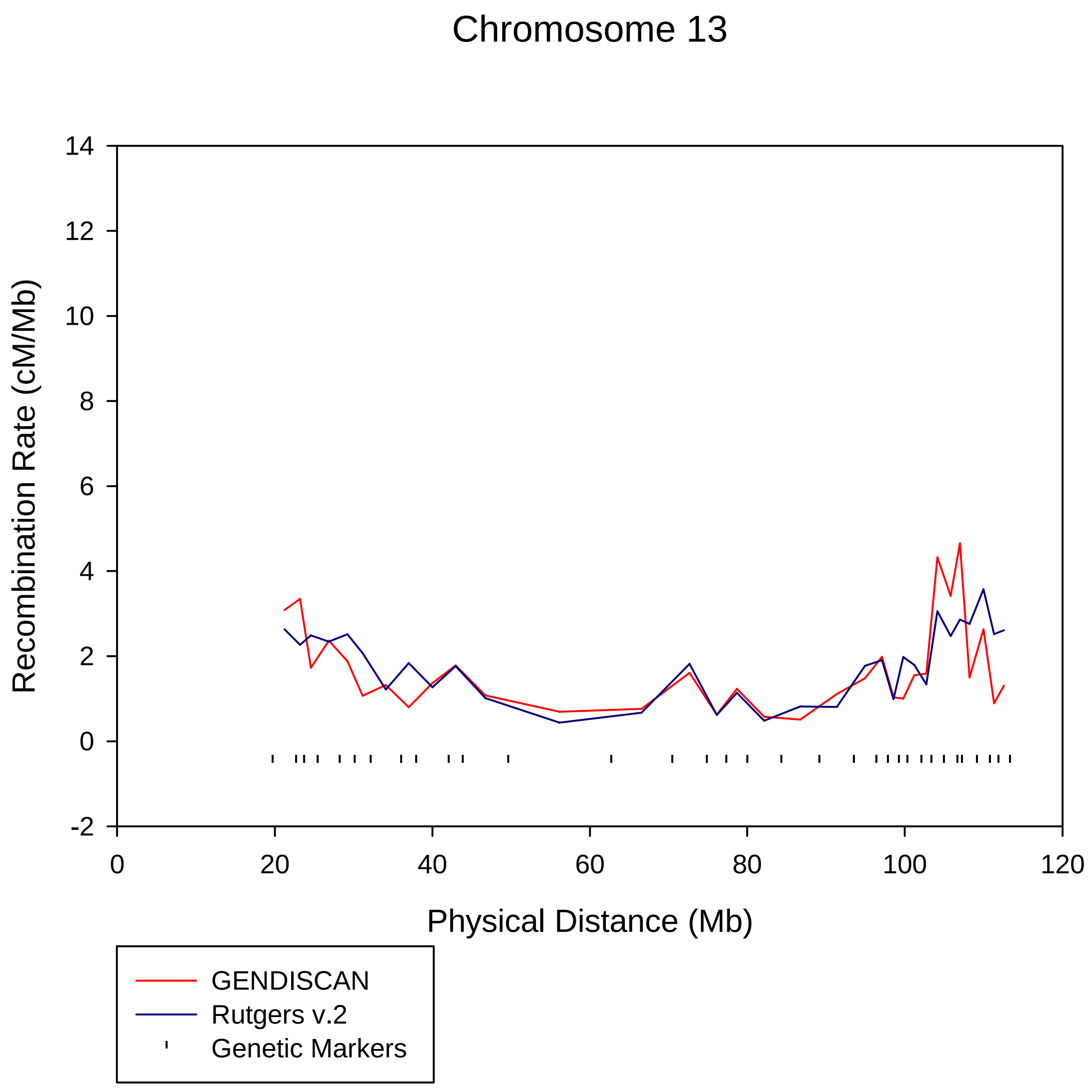

Chromosome 13
Recombination Rate (cM/Mb)
14
12
10
8
6
4
2
0
-2
0
20
40
60
80
100
120
Physical Distance (Mb)
GENDISCAN
Rutgers v.2
Genetic Markers

Chromosome 14

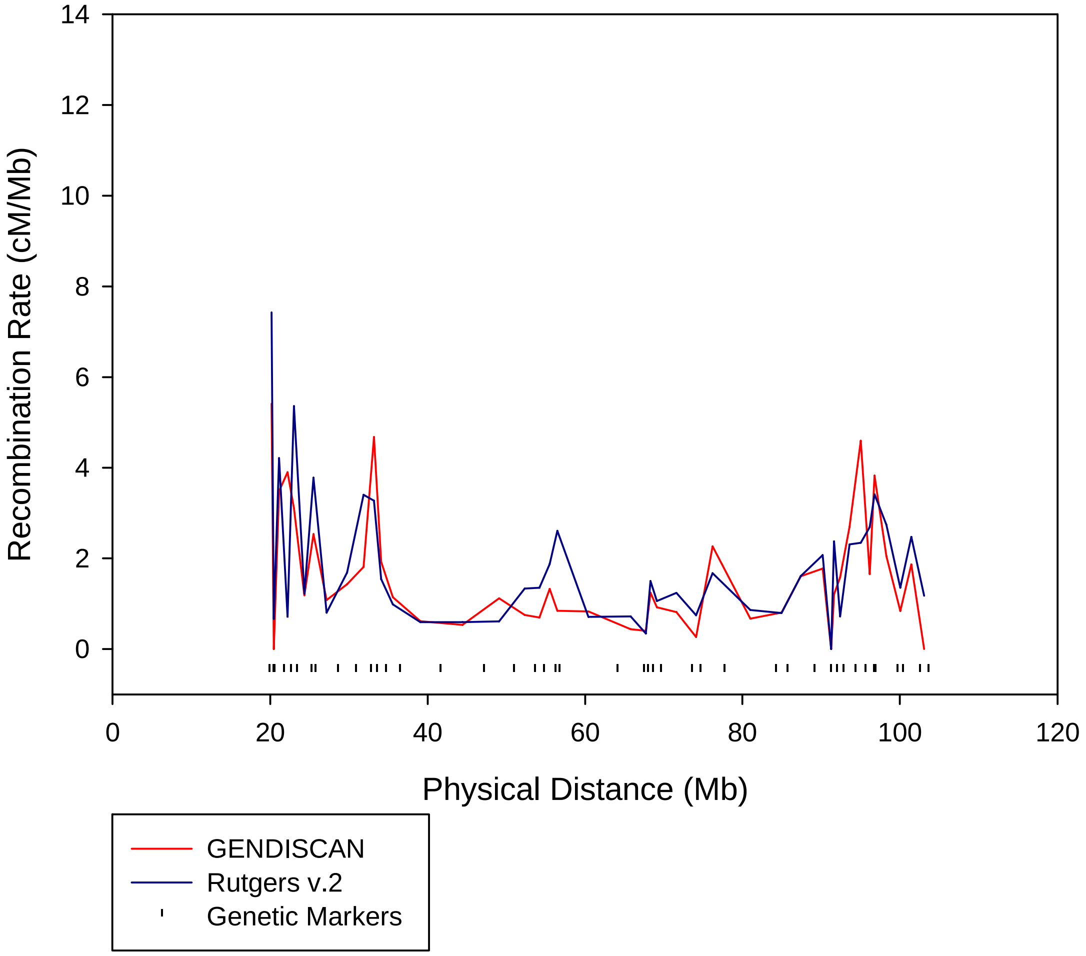

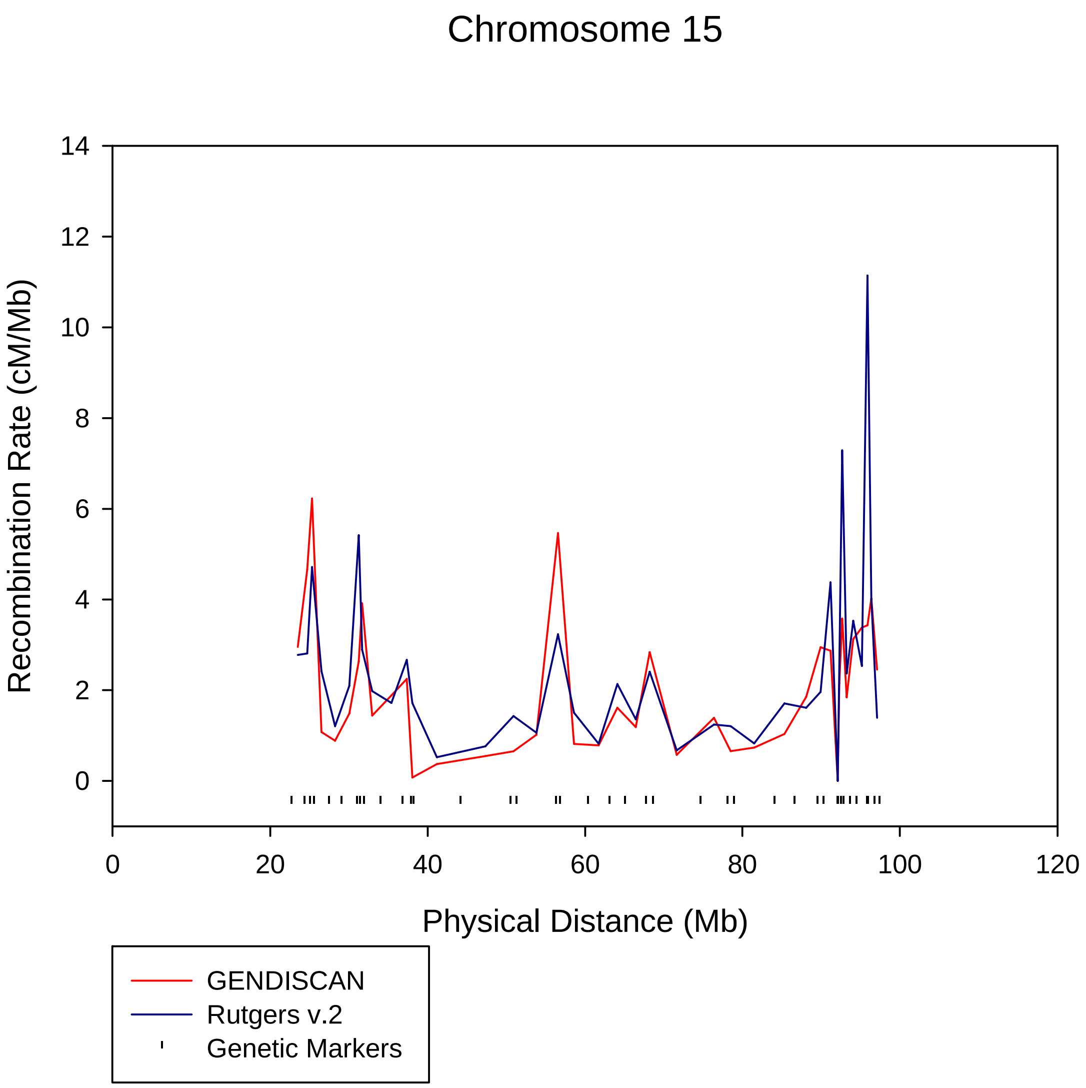

Chromosome 15
Recombination Rate (cM/Mb)
0
2
4
6
8
10
12
14
0
20
40
60
80
100
120
Physical Distance (Mb)
GENDISCAN
Rutgers v.2
Genetic Markers

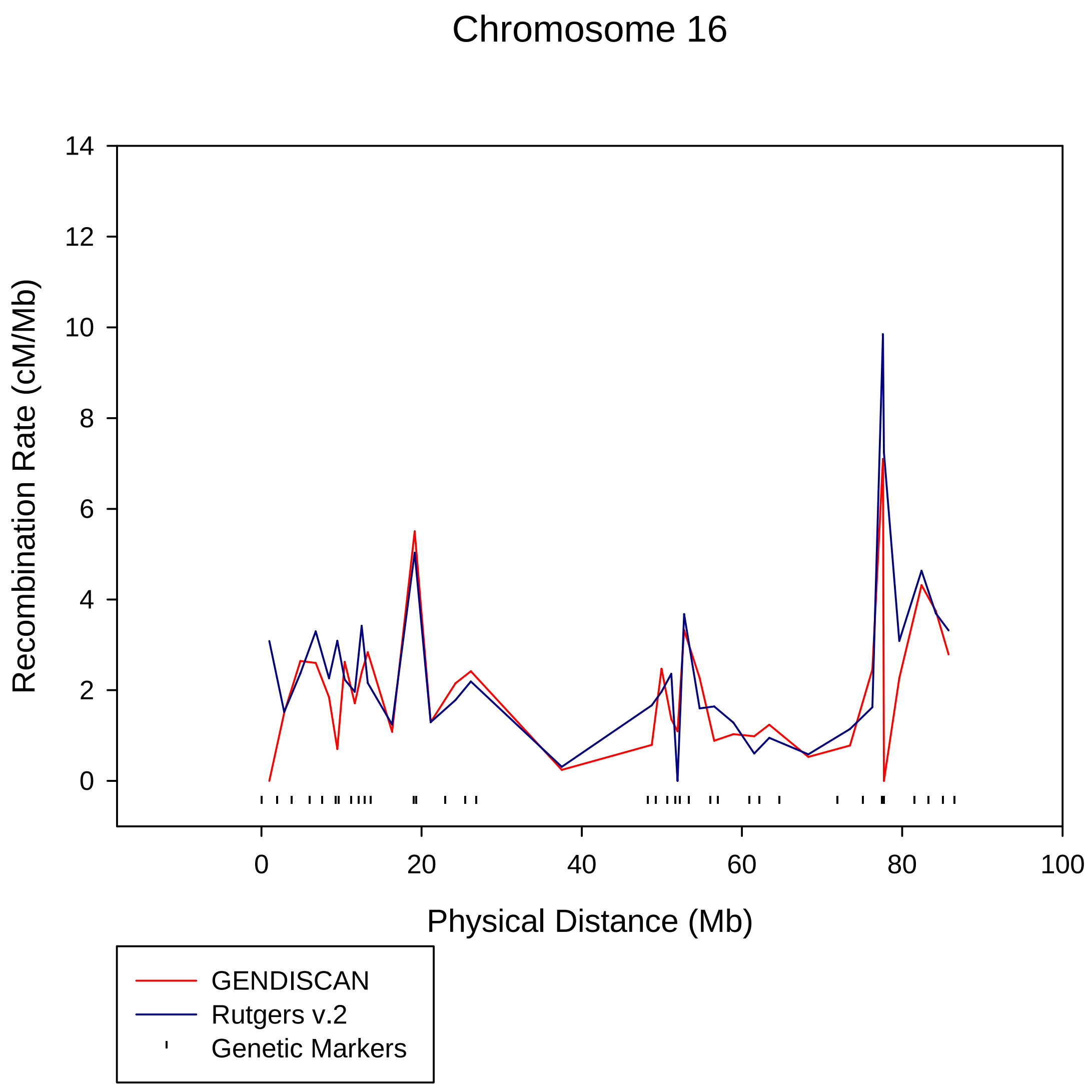
Chromosome 16
Recombination Rate (cM/Mb)
0
2
4
6
8
10
12
14
0
20
40
60
80
100
Physical Distance (Mb)
GENDISCAN
Rutgers v.2
Genetic Markers

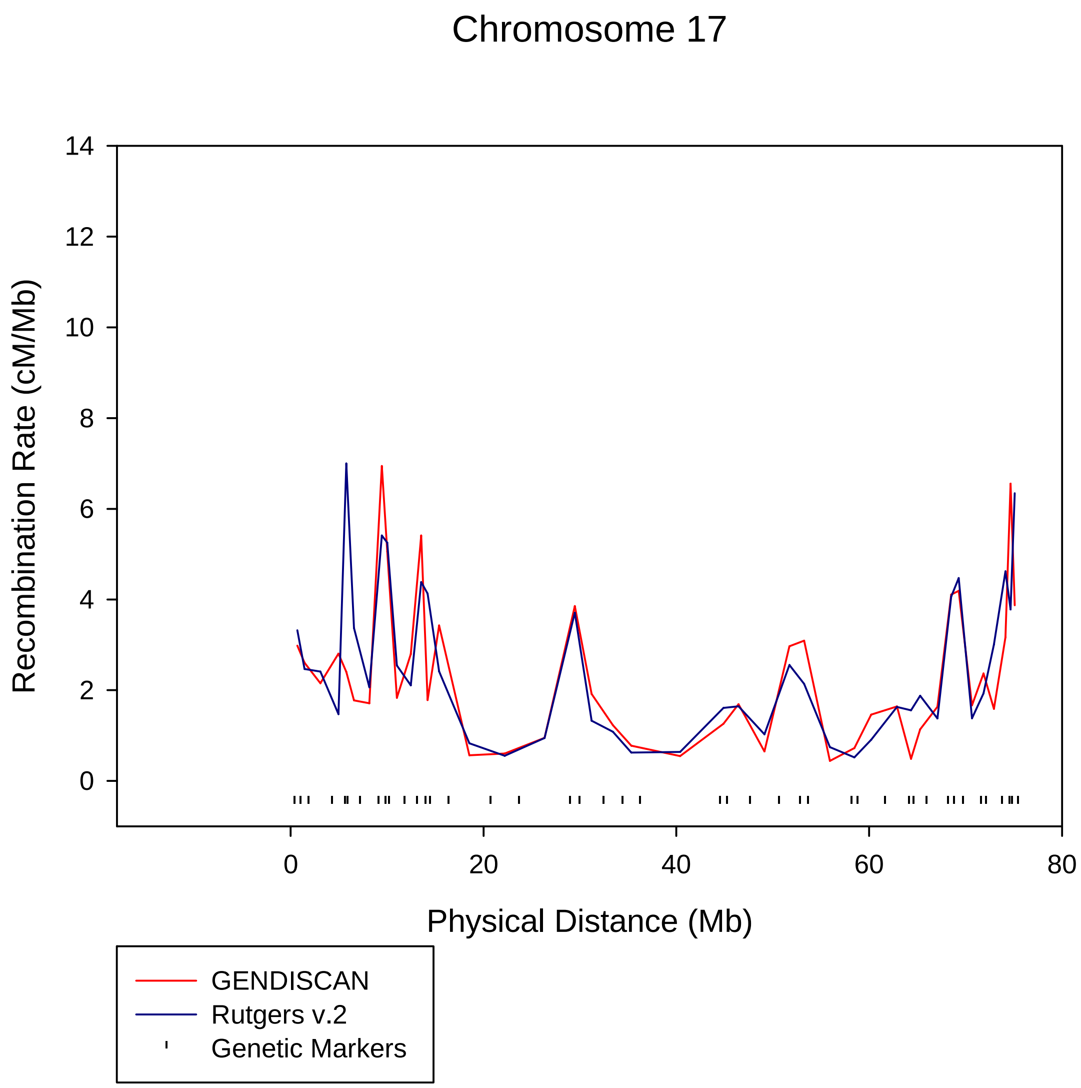
Chromosome 17
Recombination Rate (cM/Mb)
0
2
4
6
8
10
12
14
0
20
40
60
80
Physical Distance (Mb)
GENDISCAN
Rutgers v.2
Genetic Markers

Chromosome 18

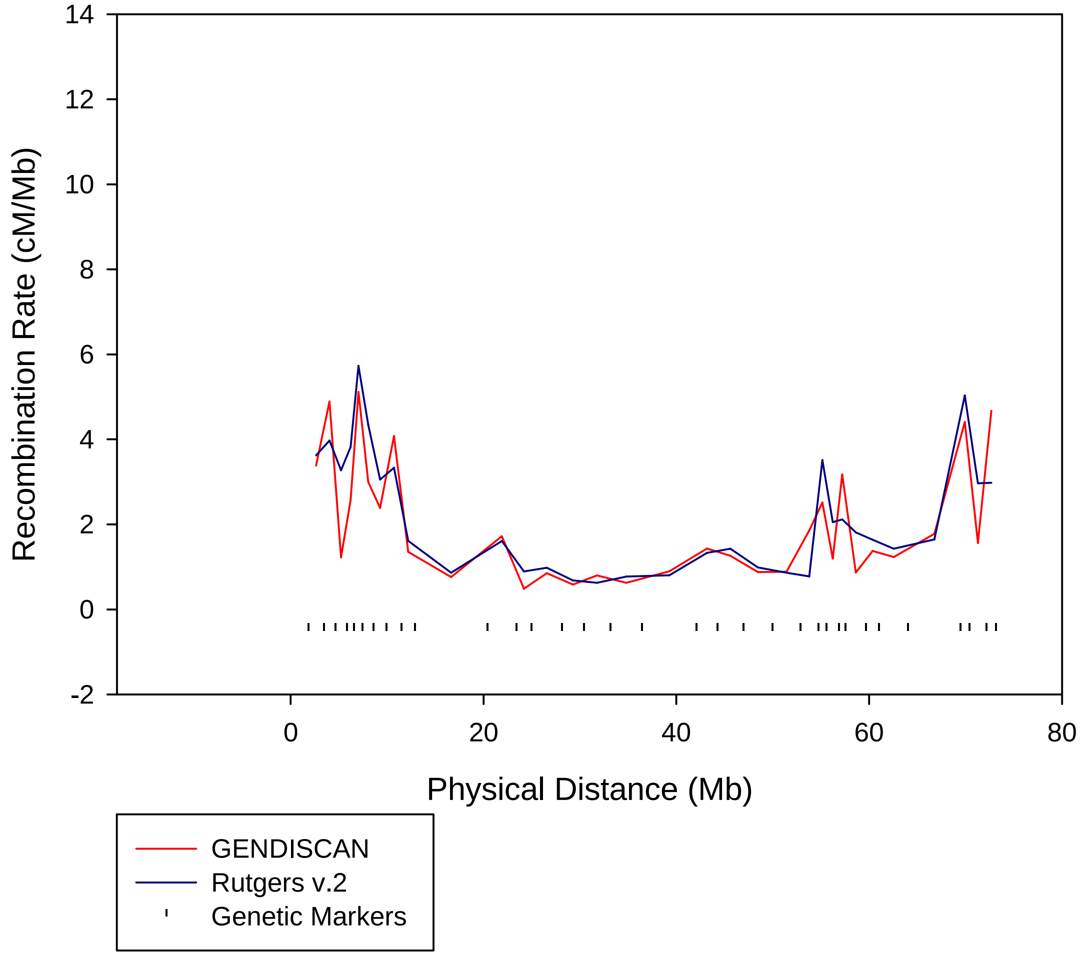

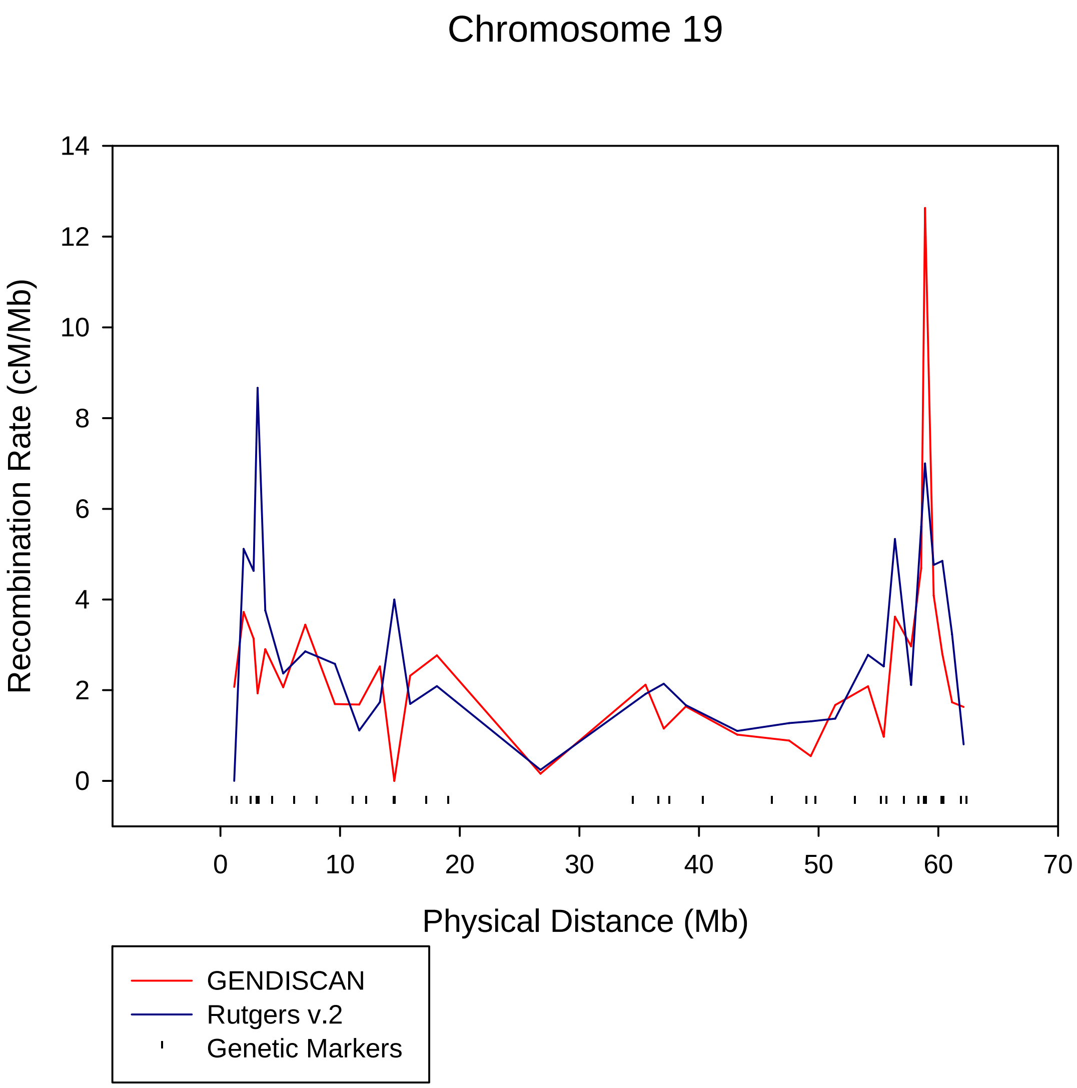

Chromosome 19
Recombination Rate (cM/Mb)
14
12
10
8
6
4
2
0
0
10
20
30
40
50
60
70
Physical Distance (Mb)
GENDISCAN
Rutgers v.2
Genetic Markers

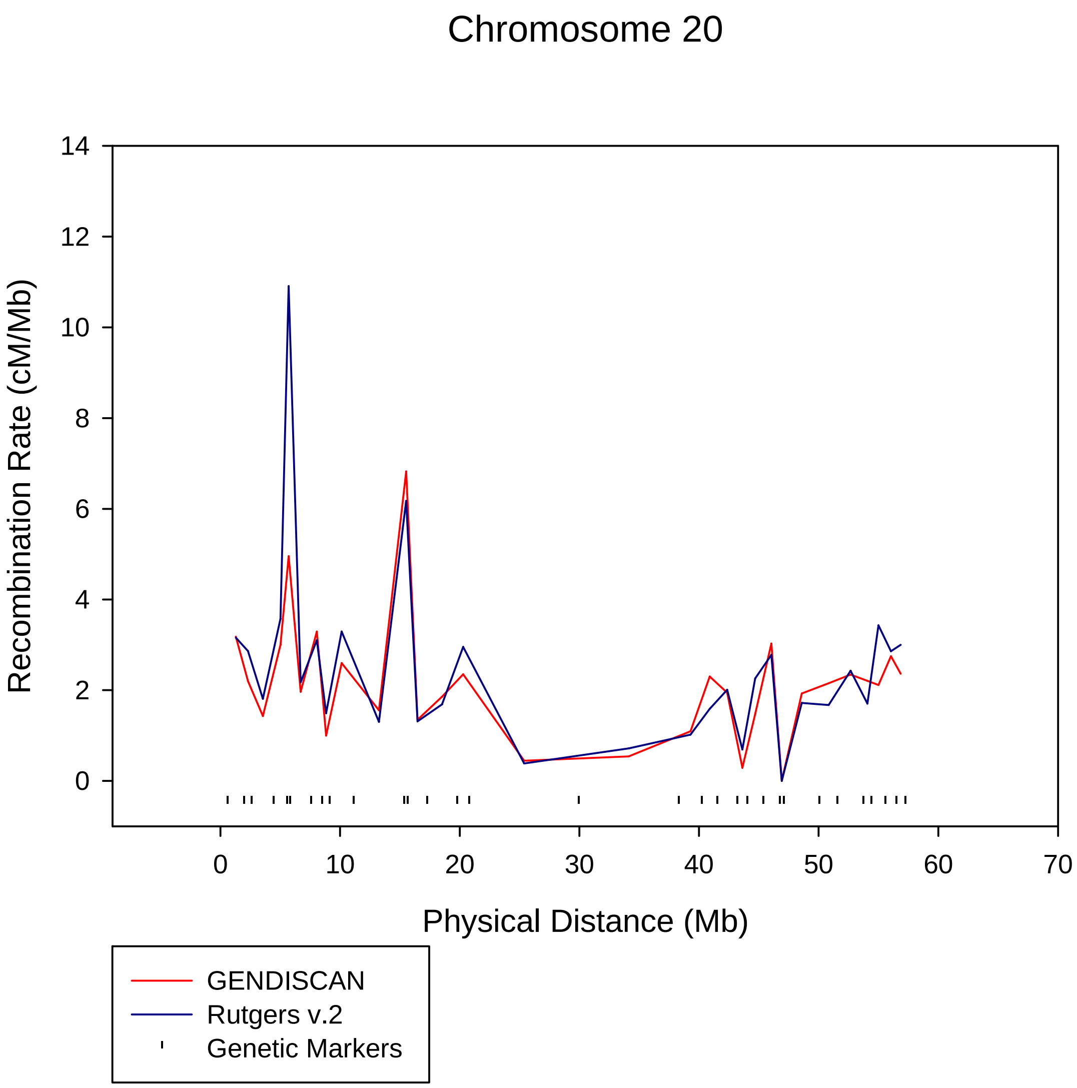

Chromosome 20
Recombination Rate (cM/Mb)
14
12
10
8
6
4
2
0
0
10
20
30
40
50
60
70
Physical Distance (Mb)
GENDISCAN
Rutgers v.2
Genetic Markers

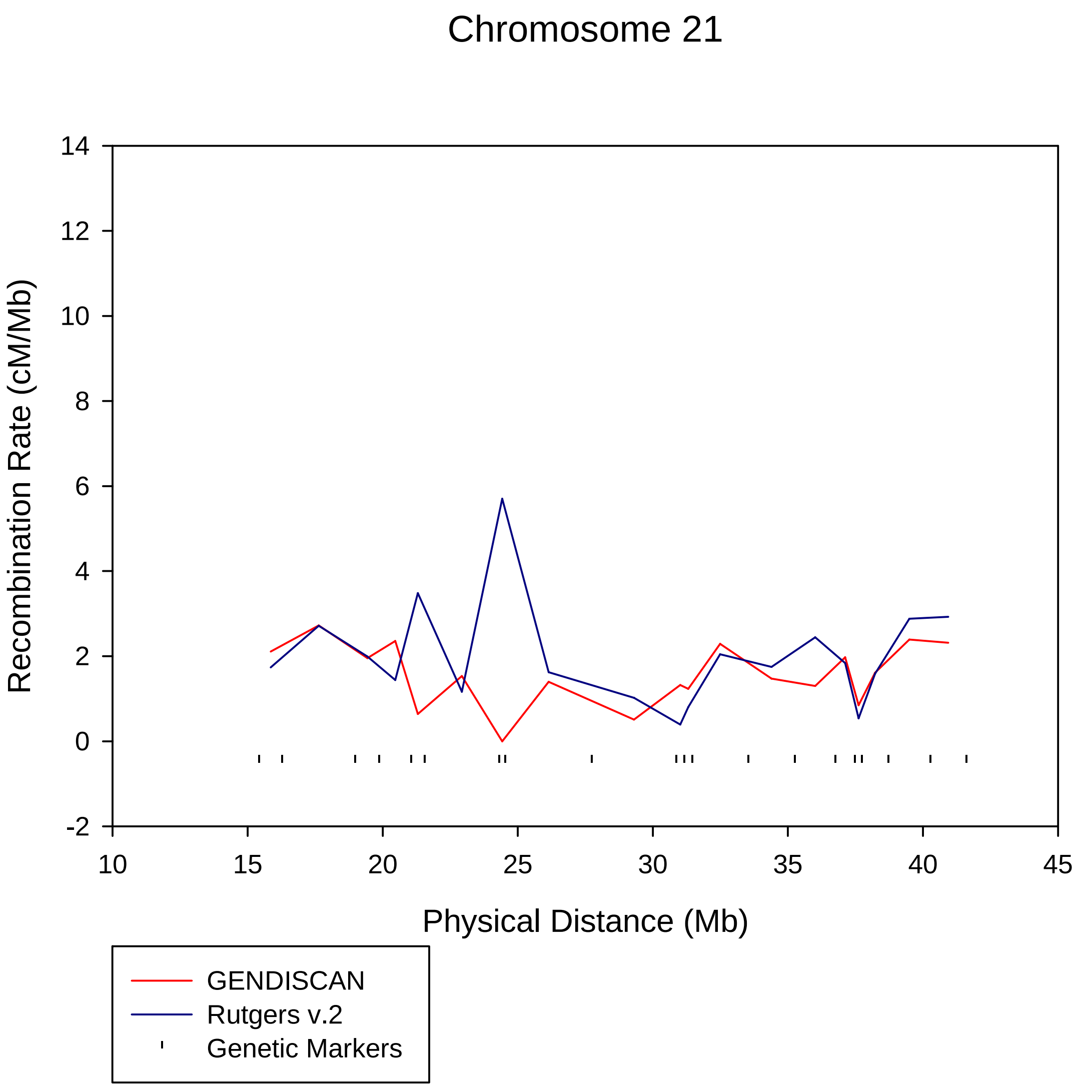

Chromosome 21
Recombination Rate (cM/Mb)
14
12
10
8
6
4
2
0
-2
10
15
20
25
30
35
40
45
Physical Distance (Mb)
GENDISCAN
Rutgers v.2
Genetic Markers

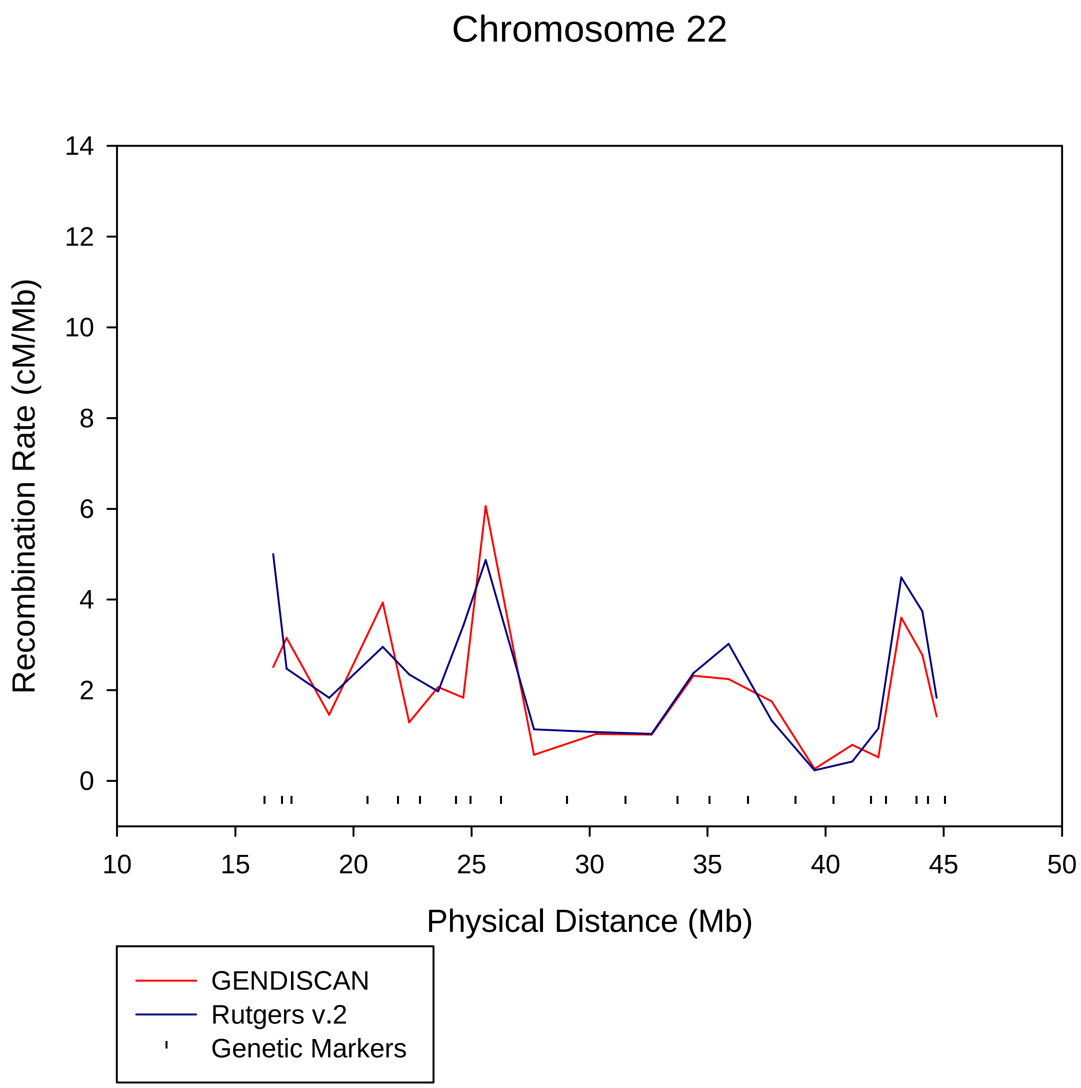

Chromosome 22
Recombination Rate (cM/Mb)
0
2
4
6
8
10
12
14
10
15
20
25
30
35
40
45
50
Physical Distance (Mb)
GENDISCAN
Rutgers v.2
Genetic Markers
