# Supplementary material for: A genome-wide Asian genetic map and ethnic comparison: The GENDISCAN study
Source: BMC Genomics. 2008 Nov 25;9:554. doi: 10.1186/1471-2164-9-554 (PMC2612022; doi:10.1186/1471-2164-9-554)

Chromosome 1

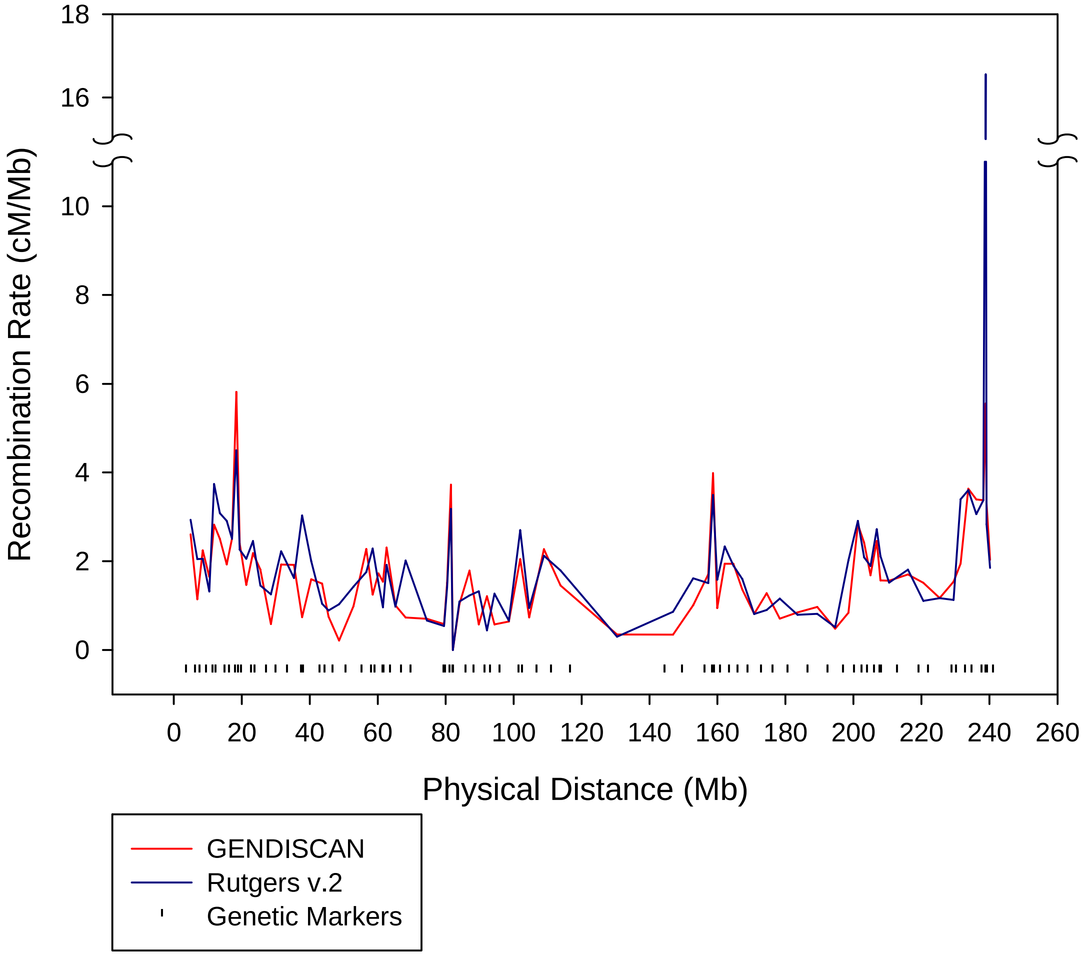

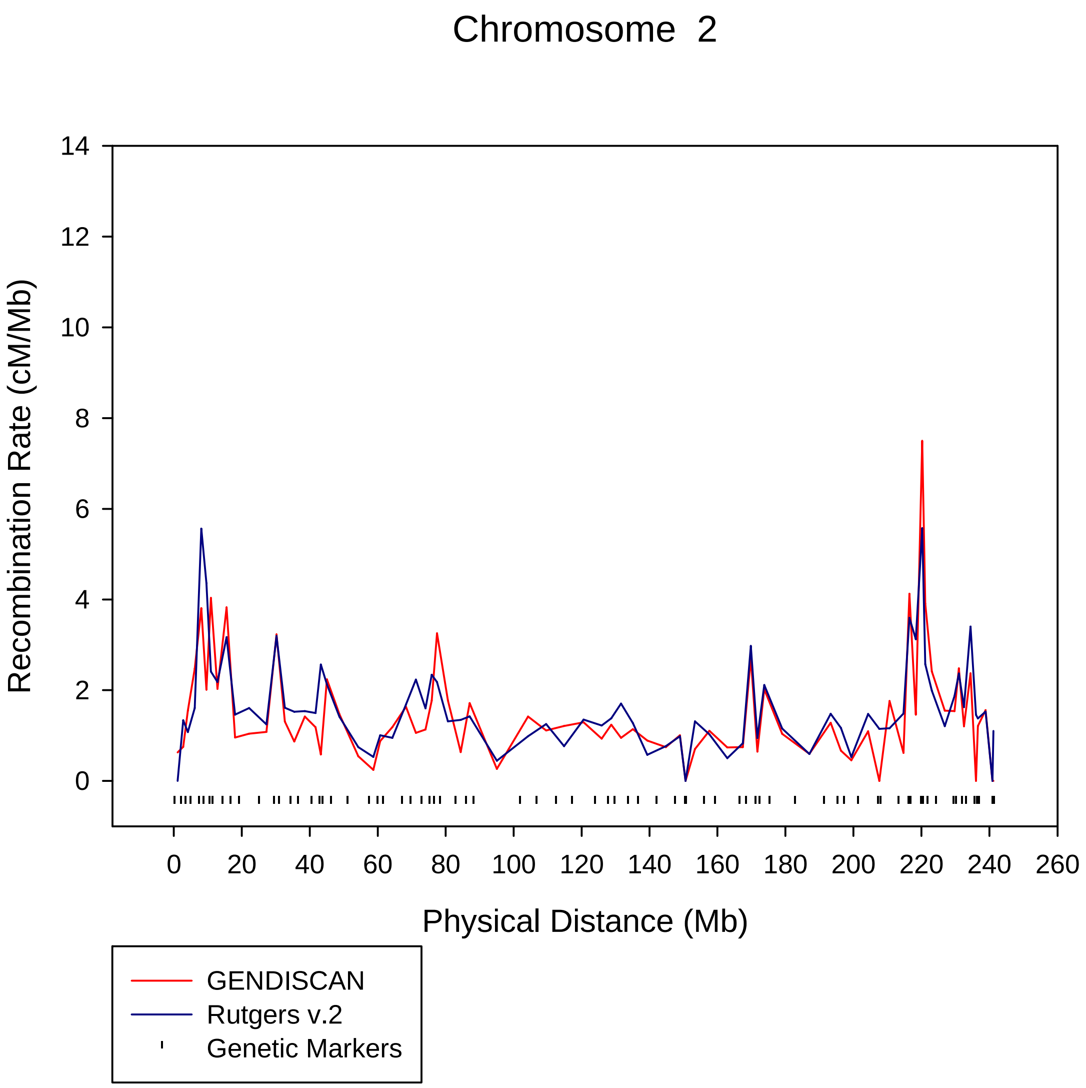

Chromosome 2
Recombination Rate (cM/Mb)
0
2
4
6
8
10
12
14
0
20
40
60
80
100
120
140
160
180
200
220
240
260
Physical Distance (Mb)
GENDISCAN
Rutgers v.2
Genetic Markers

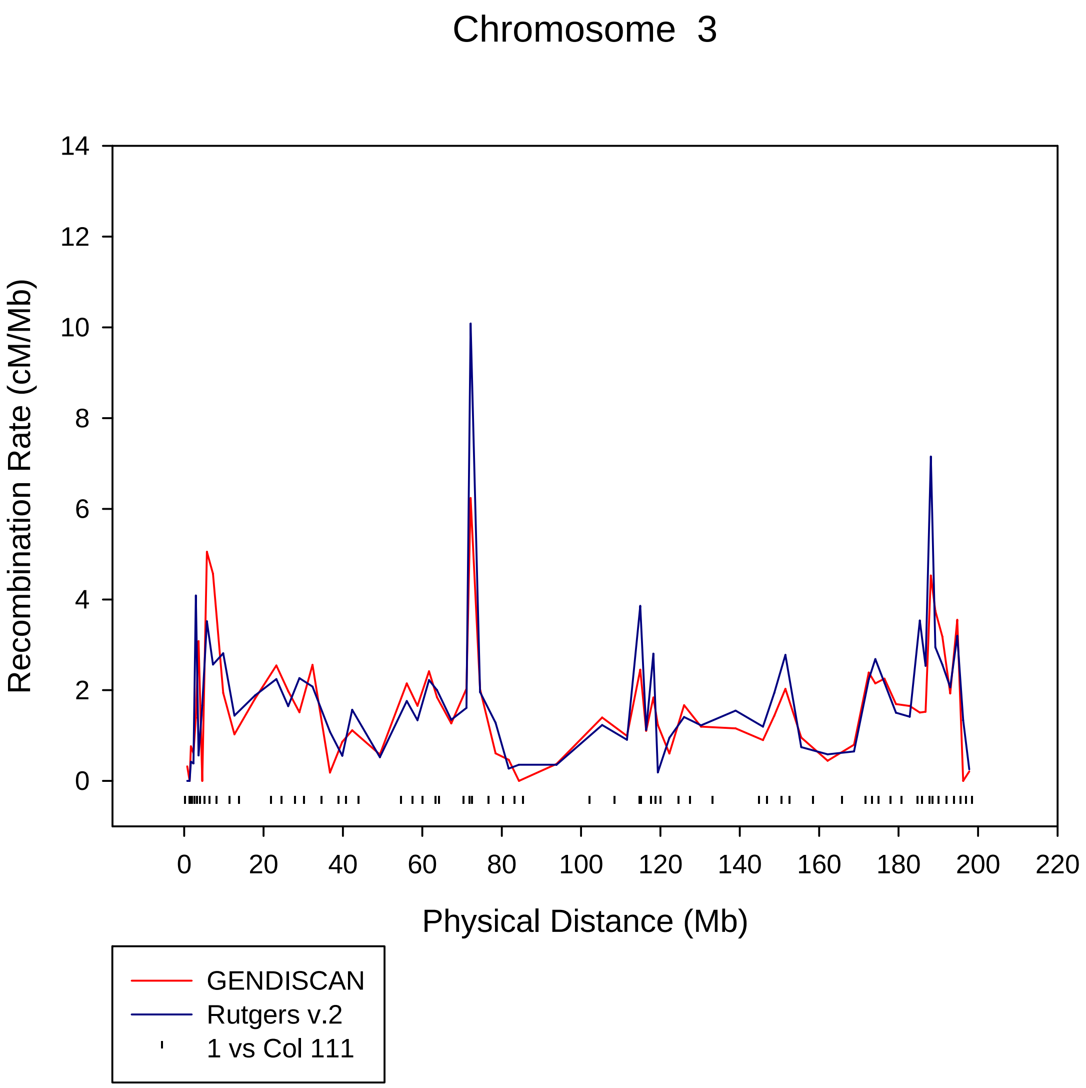

Chromosome 3
Recombination Rate (cM/Mb)
14
12
10
8
6
4
2
0
0
20
40
60
80
100
120
140
160
180
200
220
Physical Distance (Mb)
GENDISCAN
Rutgers v.2
1 vs Col 111

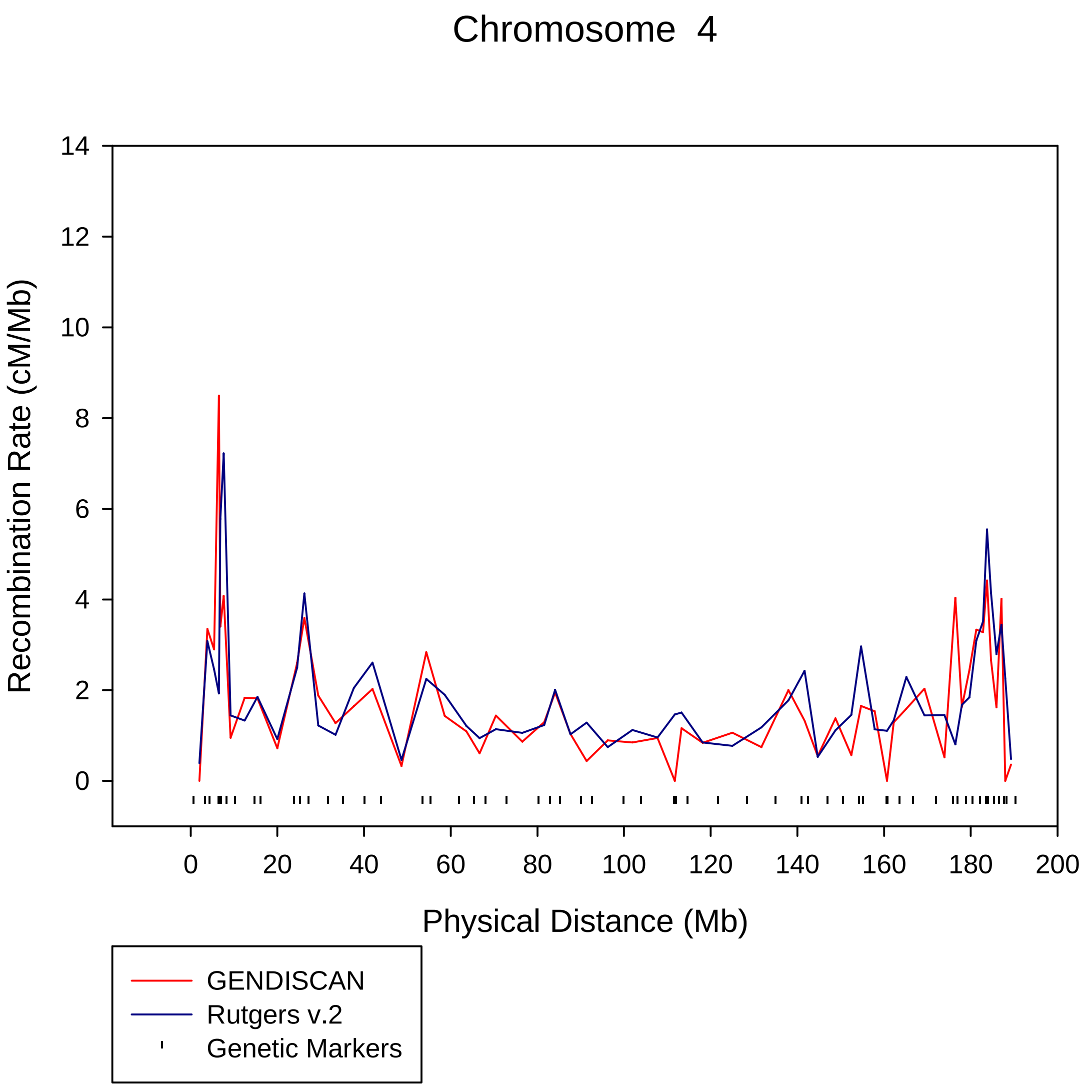
Chromosome 4
Recombination Rate (cM/Mb)
0
2
4
6
8
10
12
14
0
20
40
60
80
100
120
140
160
180
200
Physical Distance (Mb)
GENDISCAN
Rutgers v.2
Genetic Markers

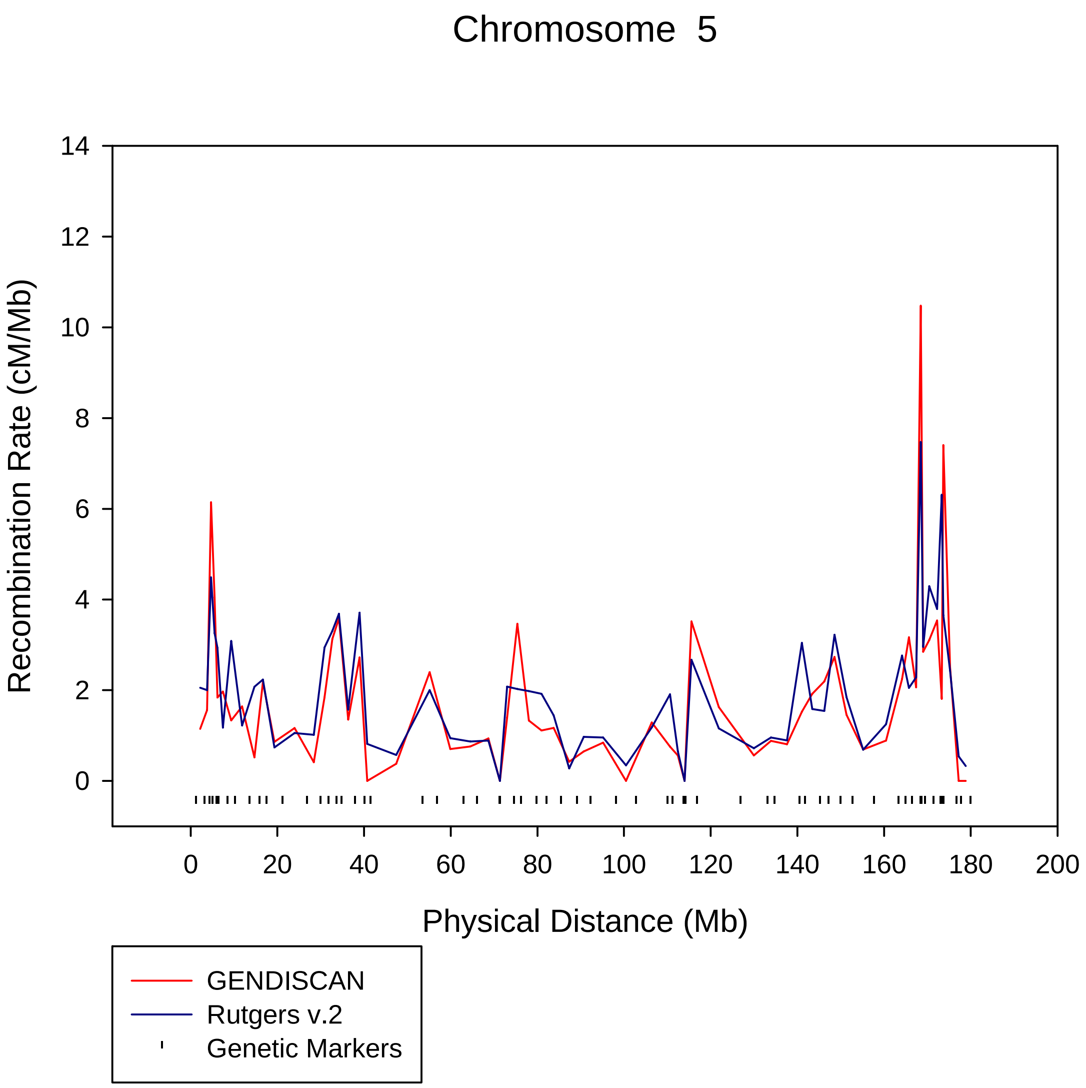
Chromosome 5
Recombination Rate (cM/Mb)
14
12
10
8
6
4
2
0
0
20
40
60
80
100
120
140
160
180
200
Physical Distance (Mb)
GENDISCAN
Rutgers v.2
Genetic Markers

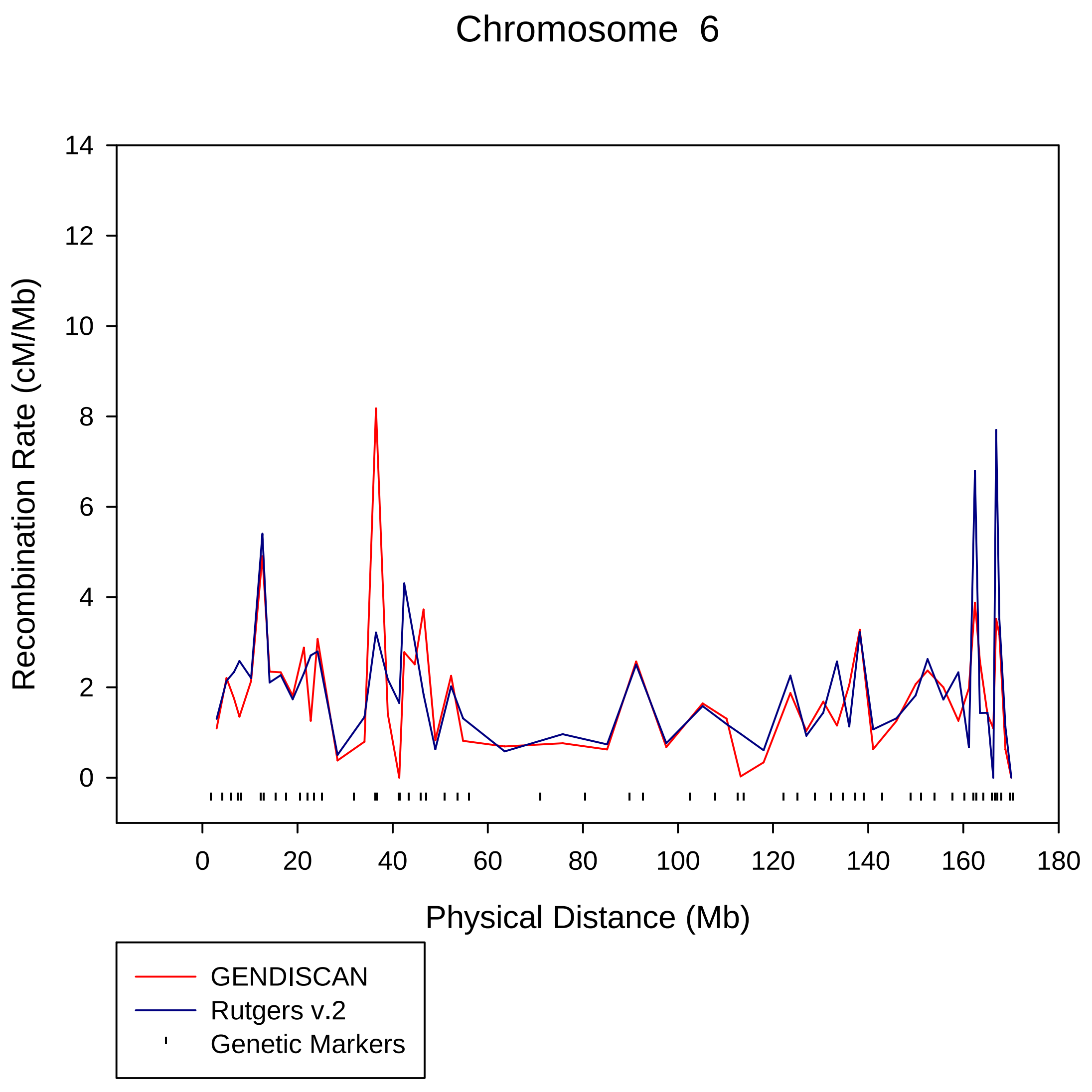
Chromosome 6
Recombination Rate (cM/Mb)
0
2
4
6
8
10
12
14
0
20
40
60
80
100
120
140
160
180
Physical Distance (Mb)
GENDISCAN
Rutgers v.2
Genetic Markers

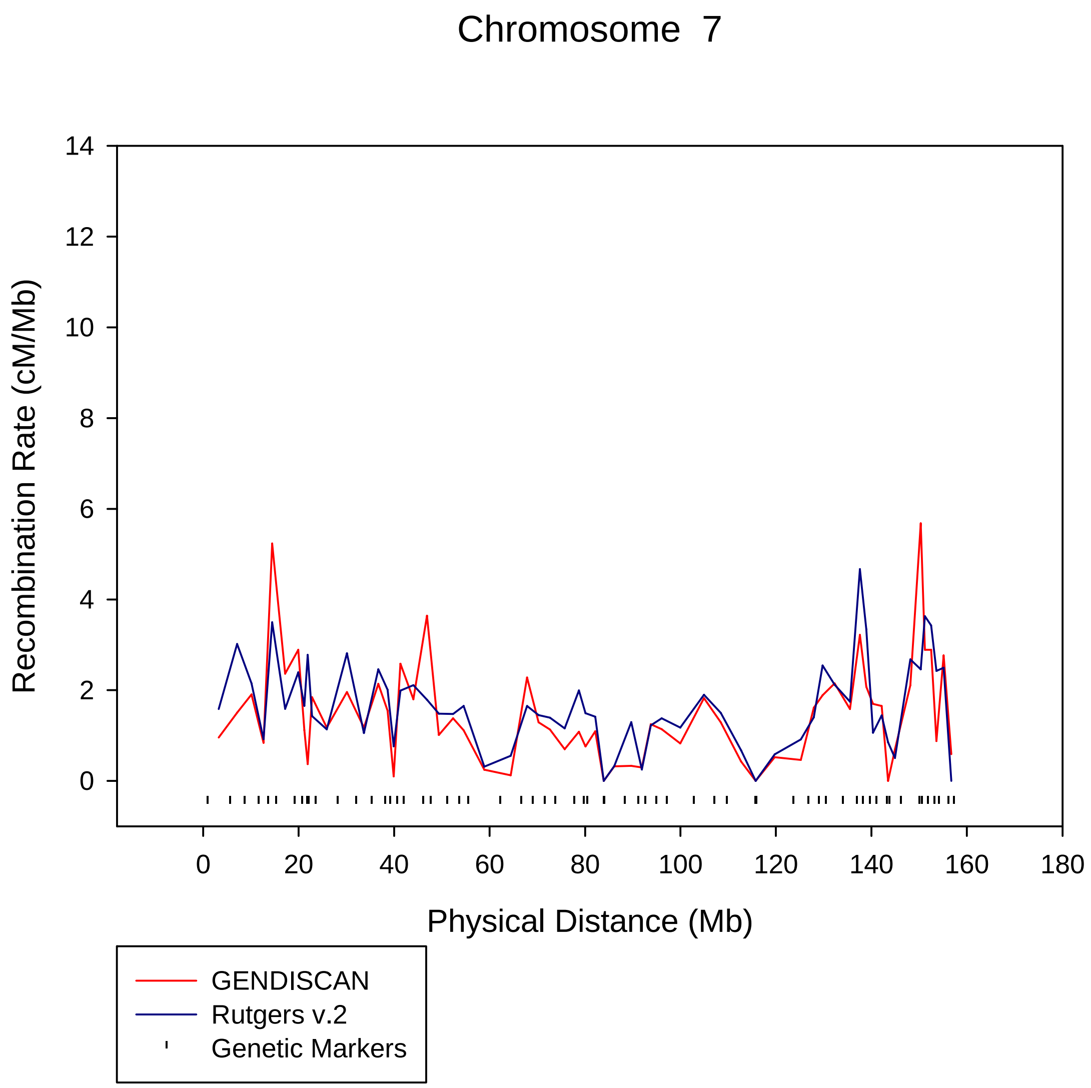
Chromosome 7
Recombination Rate (cM/Mb)
14
12
10
8
6
4
2
0
0
20
40
60
80
100
120
140
160
180
Physical Distance (Mb)
GENDISCAN
Rutgers v.2
Genetic Markers

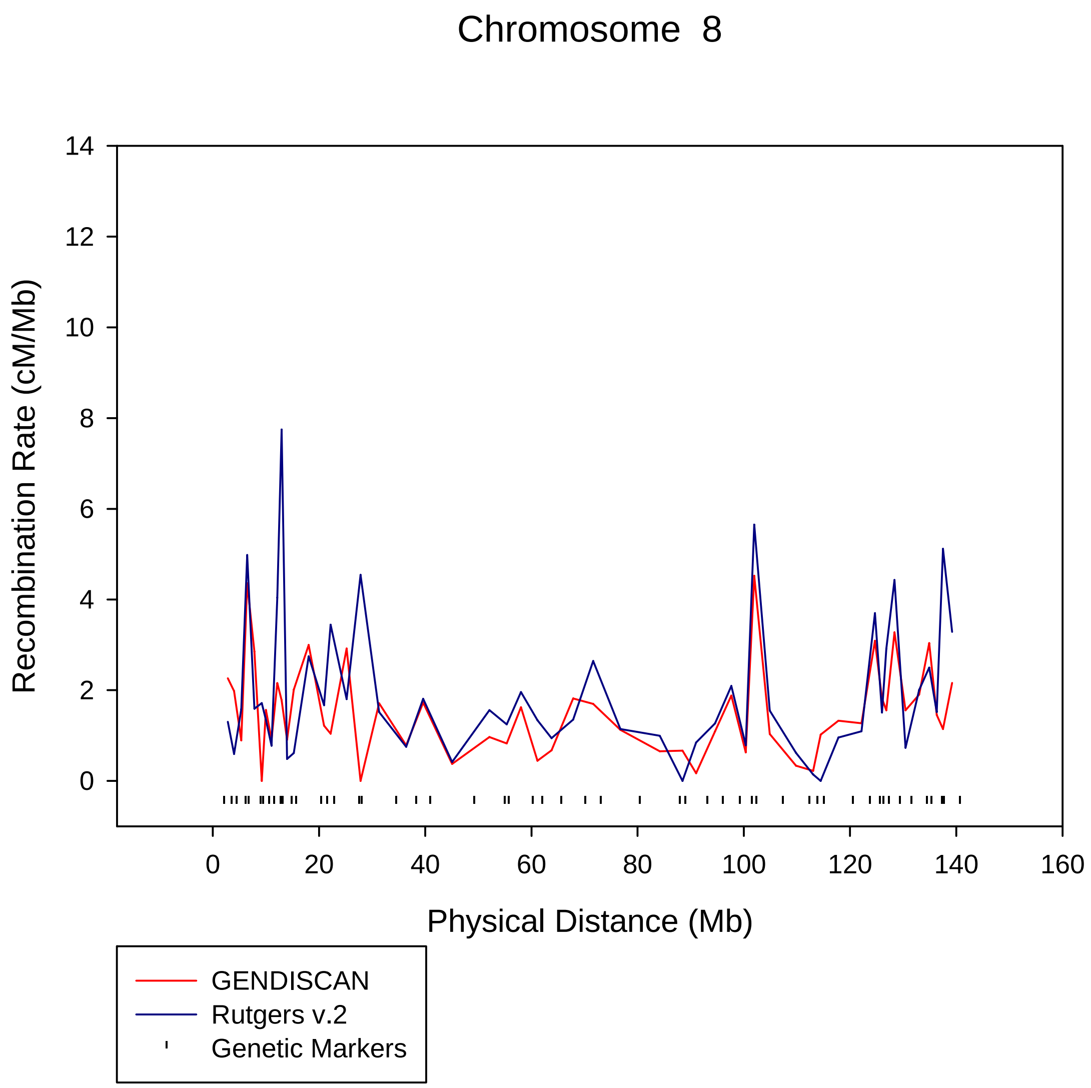
Chromosome 8
Recombination Rate (cM/Mb)
14
12
10
8
6
4
2
0
0
20
40
60
80
100
120
140
160
Physical Distance (Mb)
GENDISCAN
Rutgers v.2
Genetic Markers

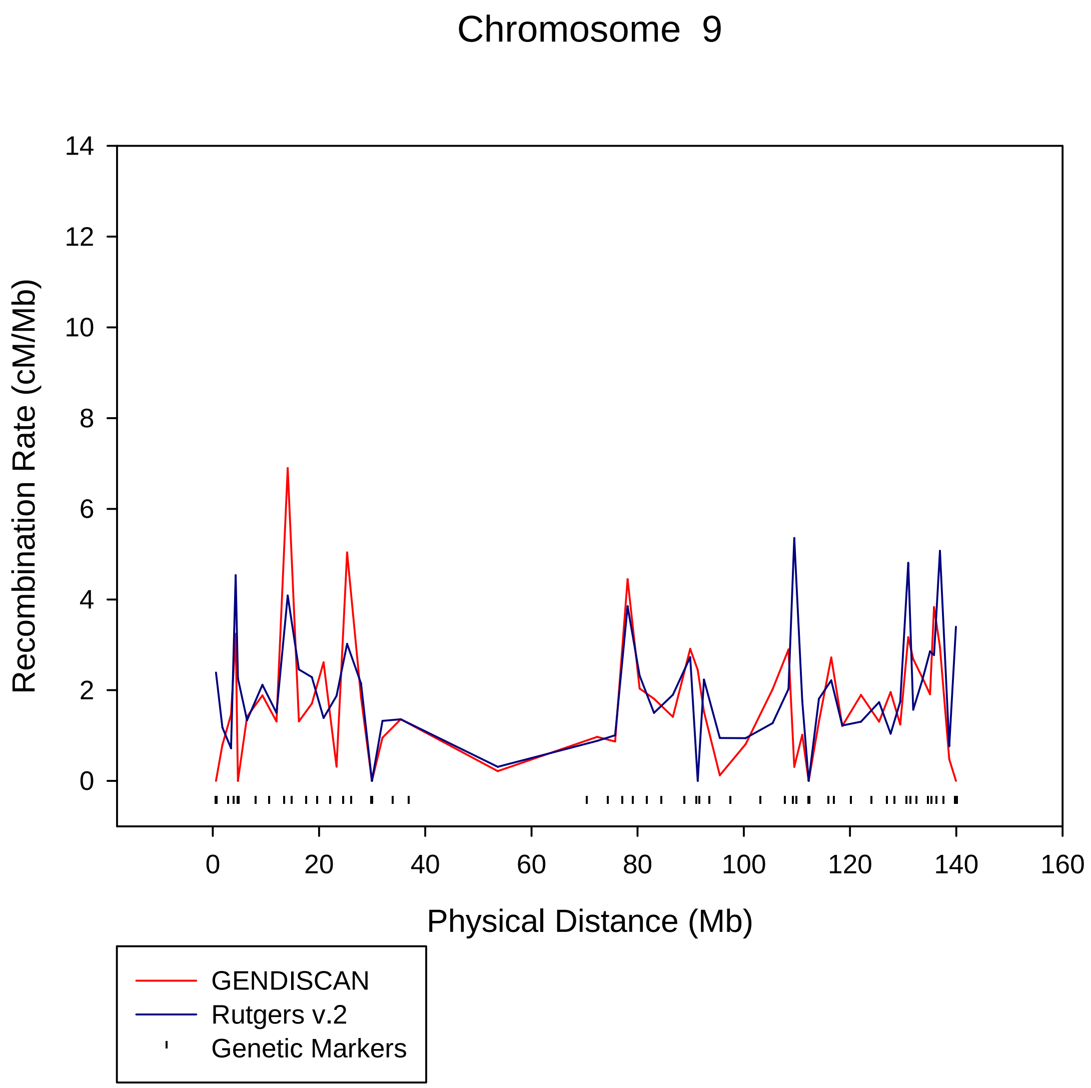

Chromosome 9
Recombination Rate (cM/Mb)
14
12
10
8
6
4
2
0
0
20
40
60
80
100
120
140
160
Physical Distance (Mb)
GENDISCAN
Rutgers v.2
Genetic Markers

Chromosome 10

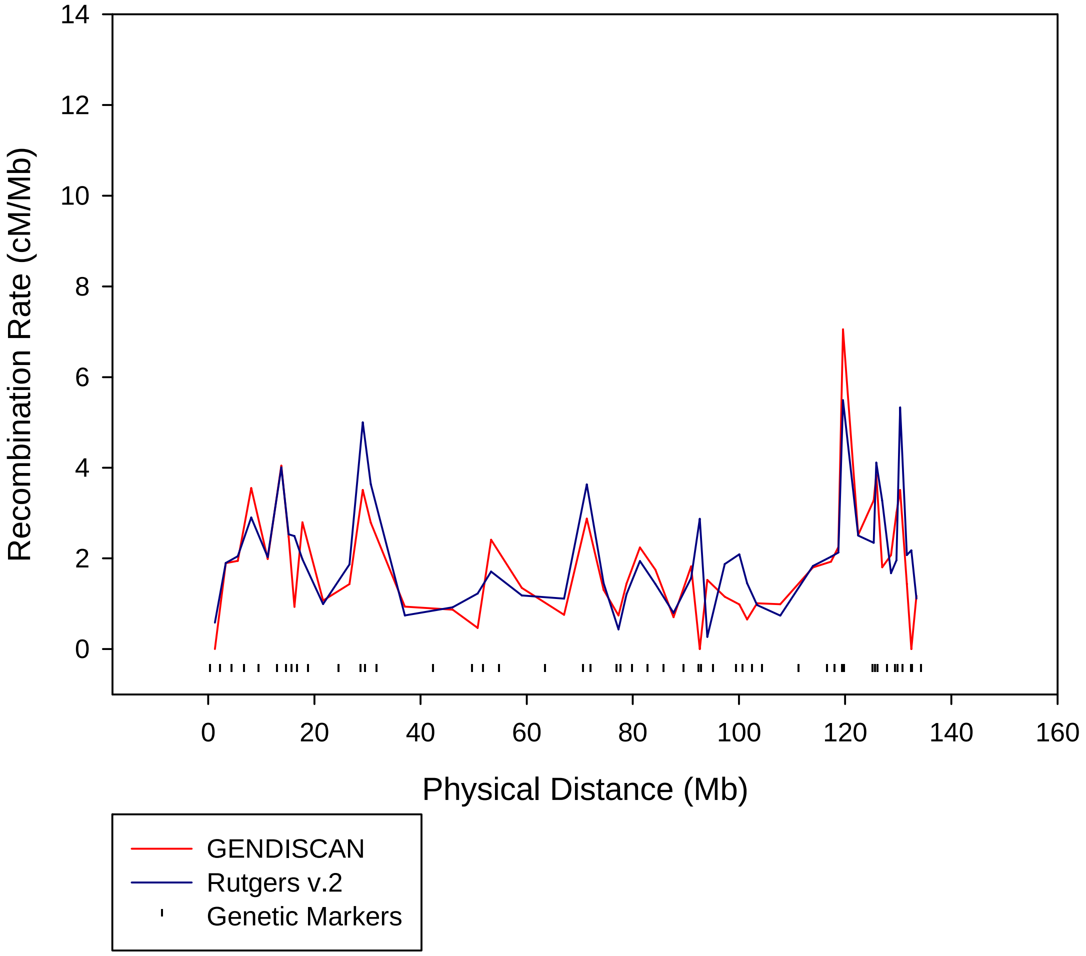

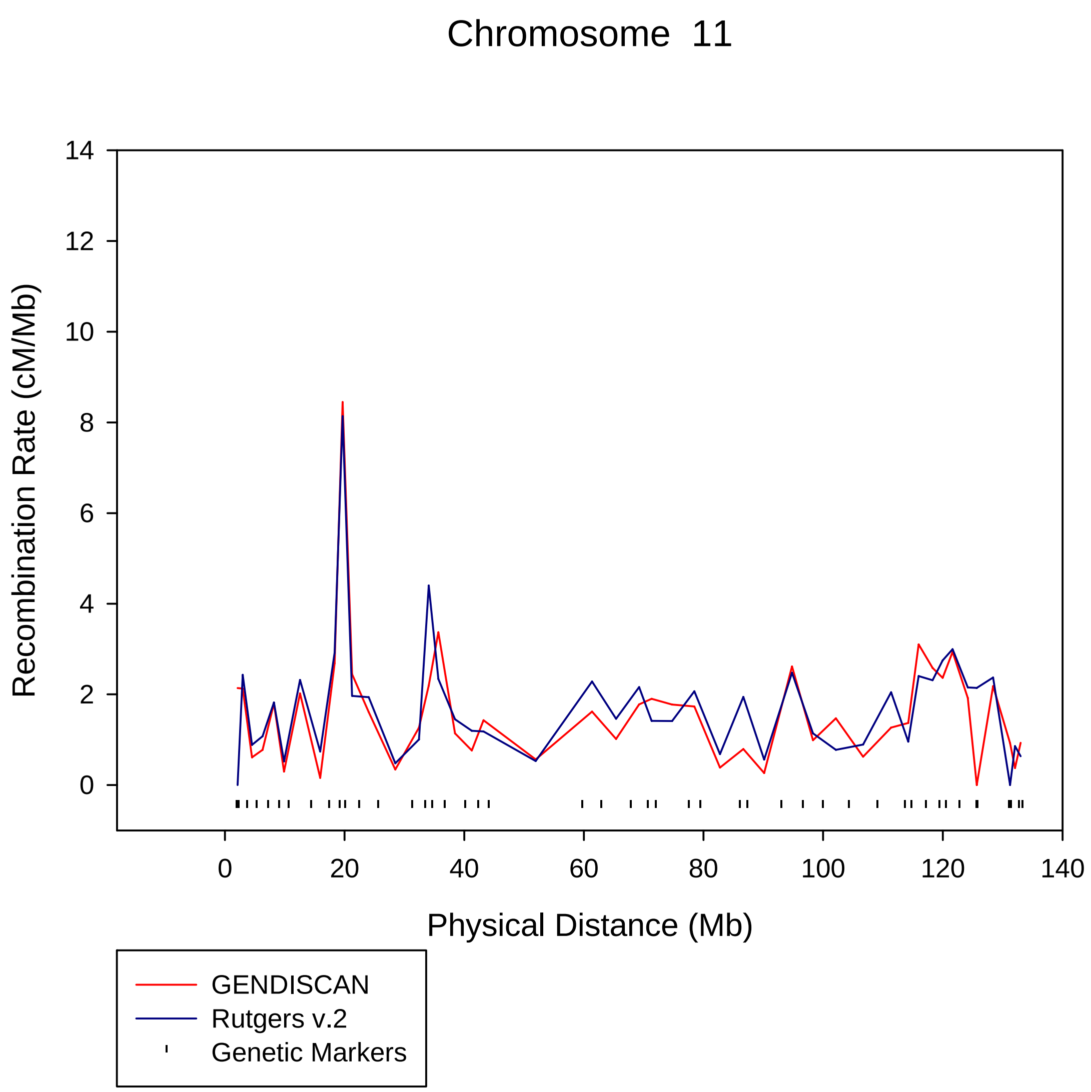
Chromosome 11
Recombination Rate (cM/Mb)
14
12
10
8
6
4
2
0
0
20
40
60
80
100
120
140
Physical Distance (Mb)
GENDISCAN
Rutgers v.2
Genetic Markers

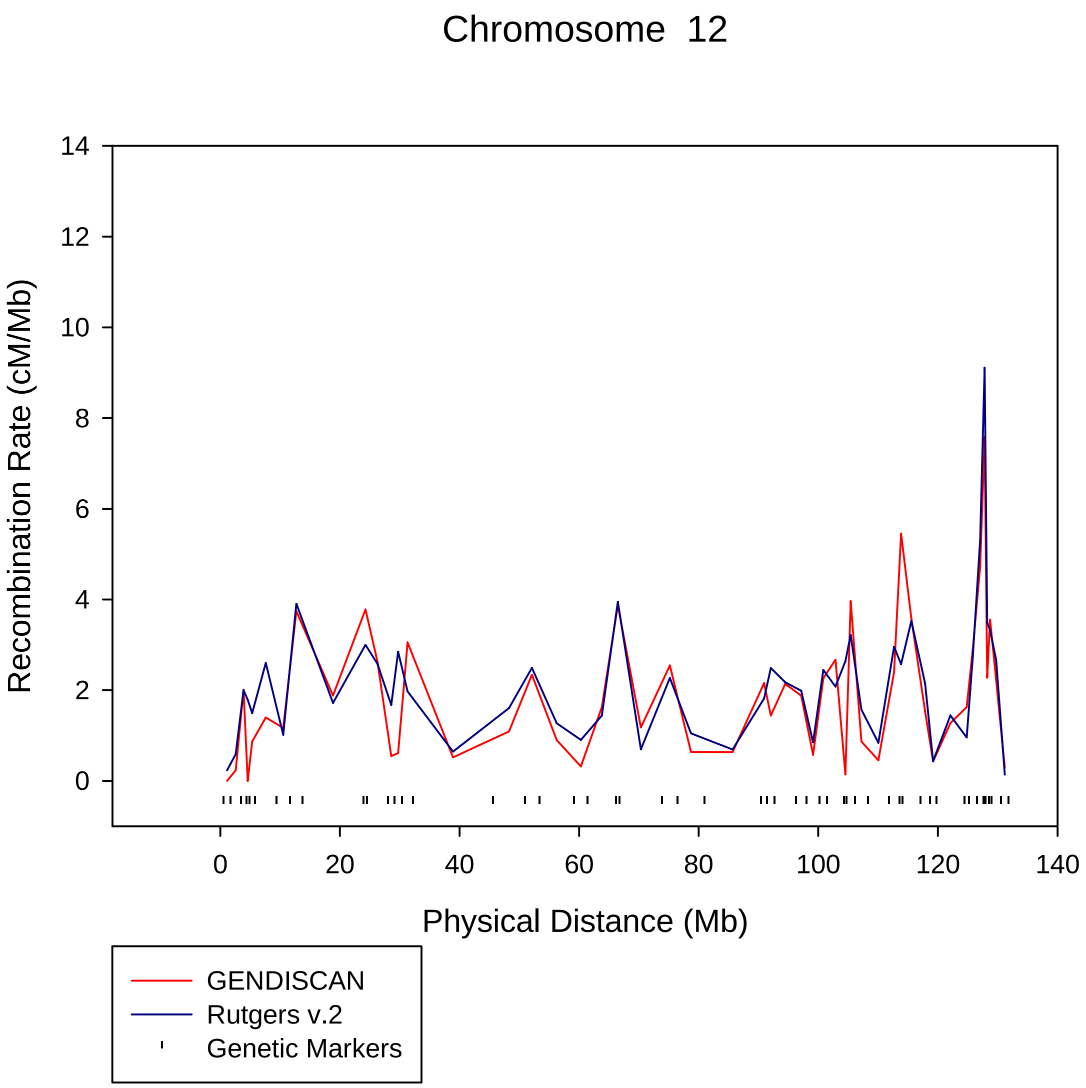

Chromosome 12
Recombination Rate (cM/Mb)
0
2
4
6
8
10
12
14
0
20
40
60
80
100
120
140
Physical Distance (Mb)
GENDISCAN
Rutgers v.2
Genetic Markers

Chromosome 13

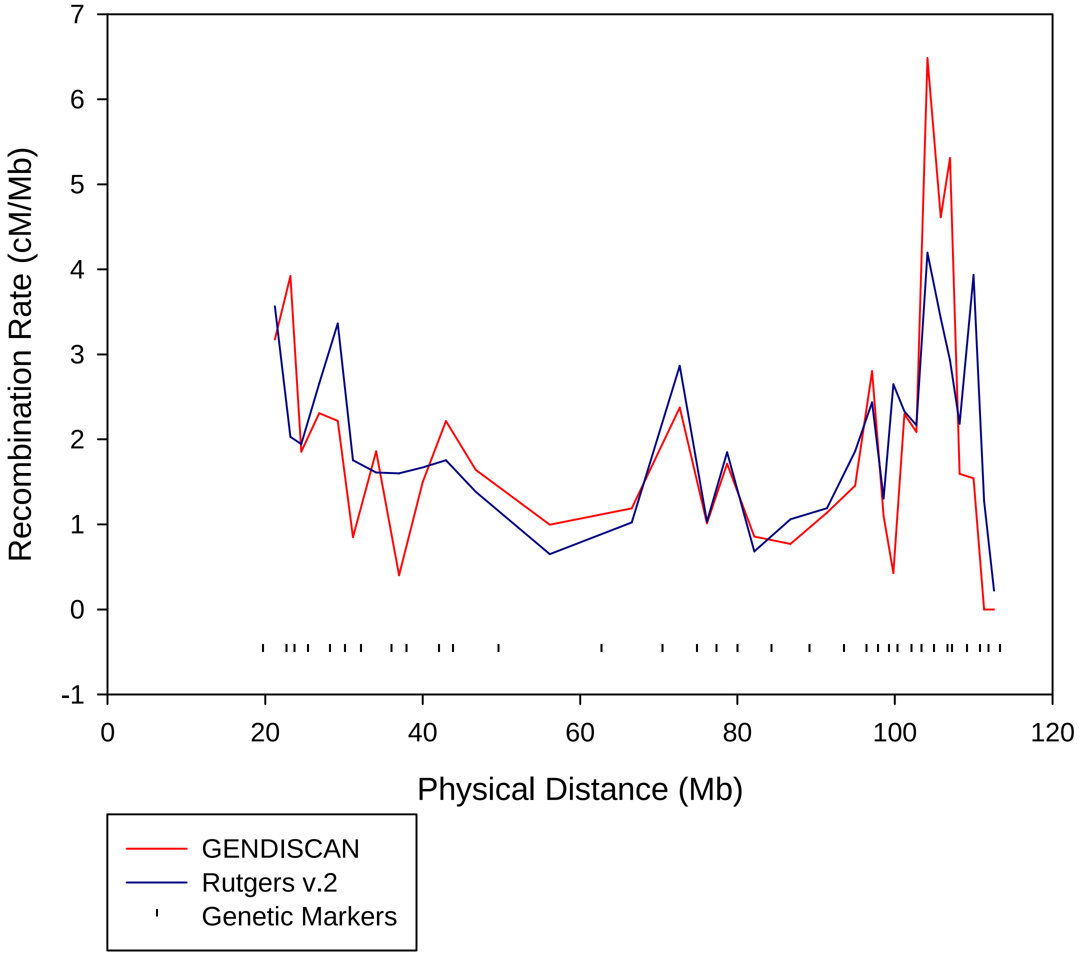

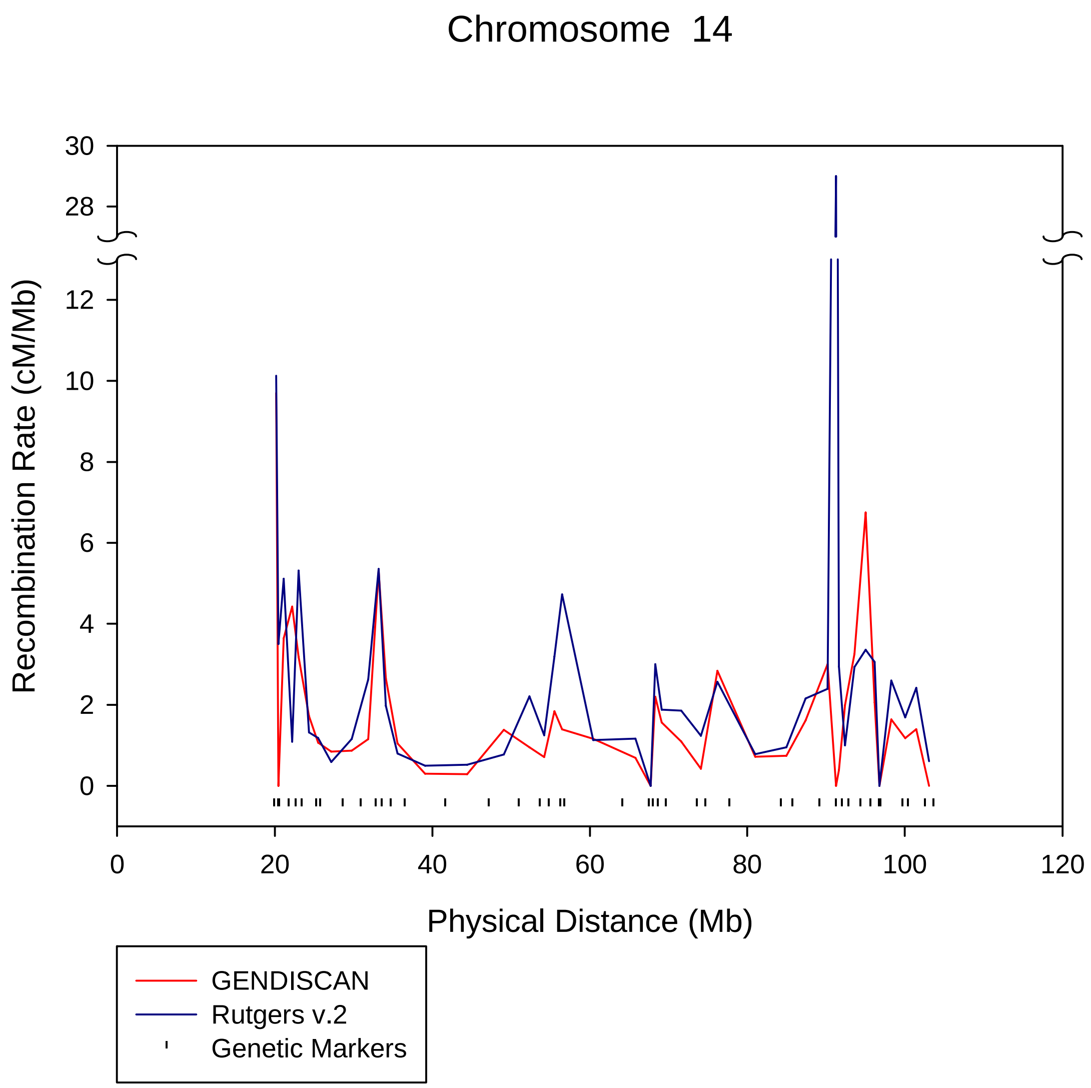

Chromosome 14
Recombination Rate (cM/Mb)
30
28
12
10
8
6
4
2
0
0
20
40
60
80
100
120
Physical Distance (Mb)
GENDISCAN
Rutgers v.2
Genetic Markers

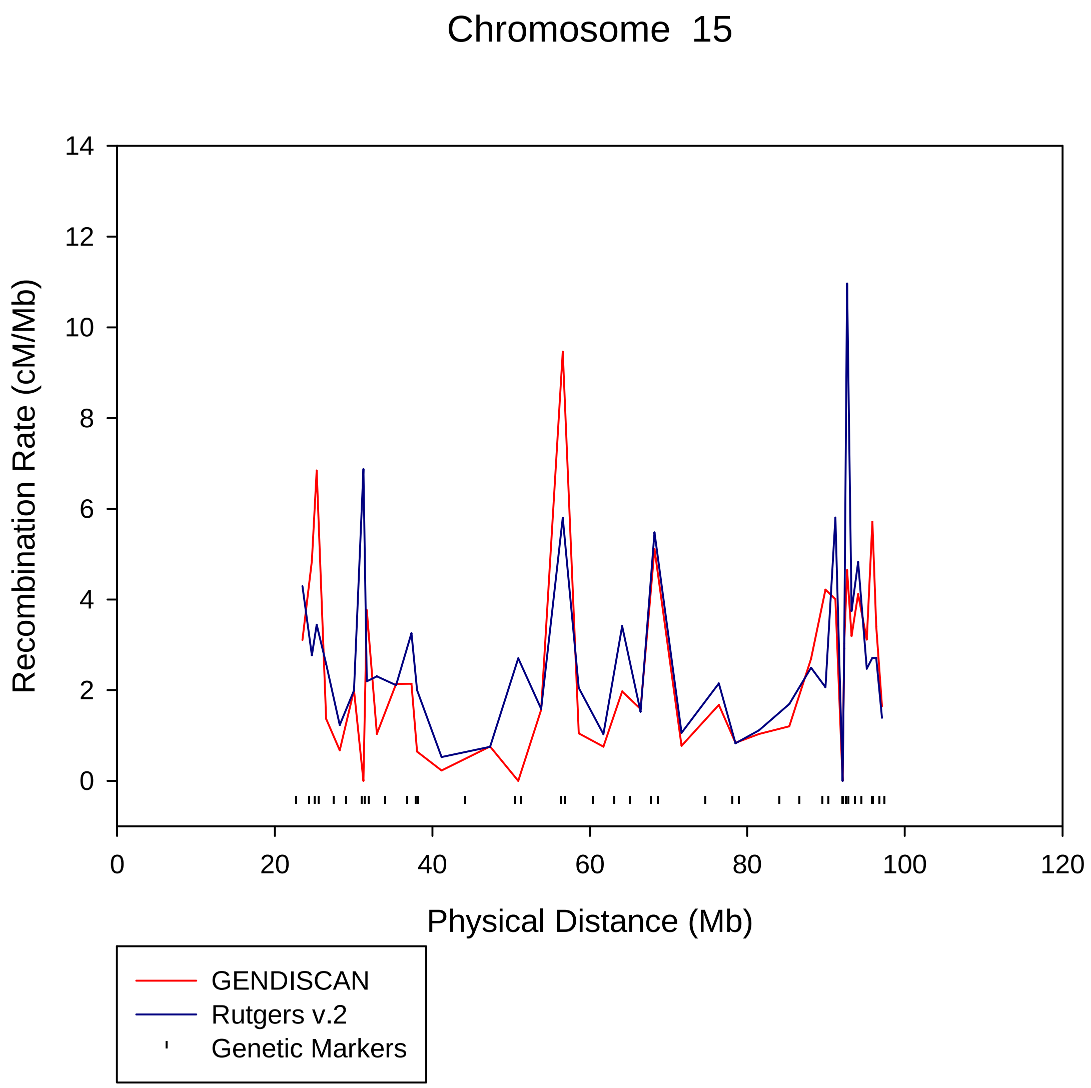
Chromosome 15
Recombination Rate (cM/Mb)
0
2
4
6
8
10
12
14
0
20
40
60
80
100
120
Physical Distance (Mb)
GENDISCAN
Rutgers v.2
Genetic Markers

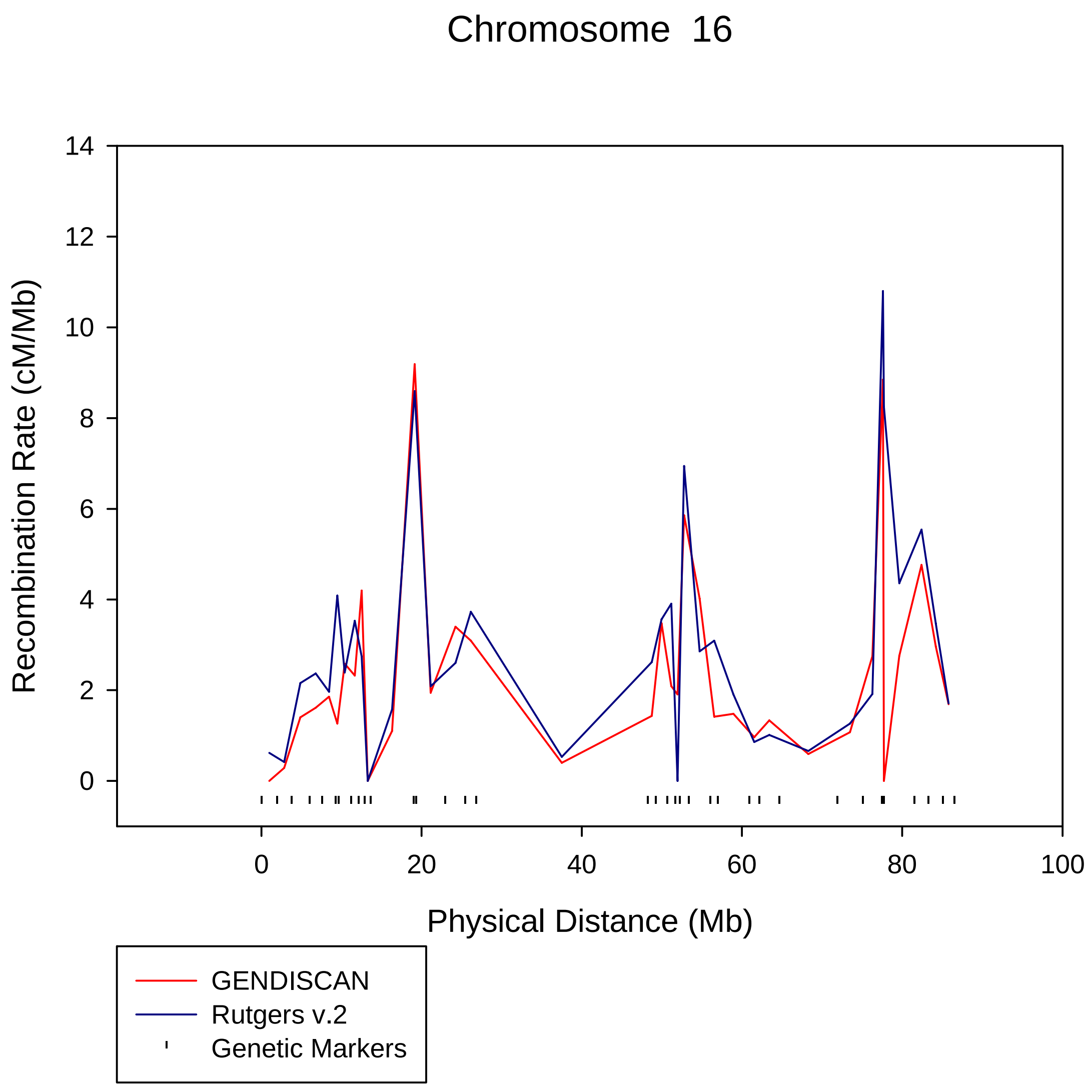
Chromosome 16
Recombination Rate (cM/Mb)
Physical Distance (Mb)
0
2
4
6
8
10
12
14
0
20
40
60
80
100
GENDISCAN
Rutgers v.2
Genetic Markers

Chromosome 17

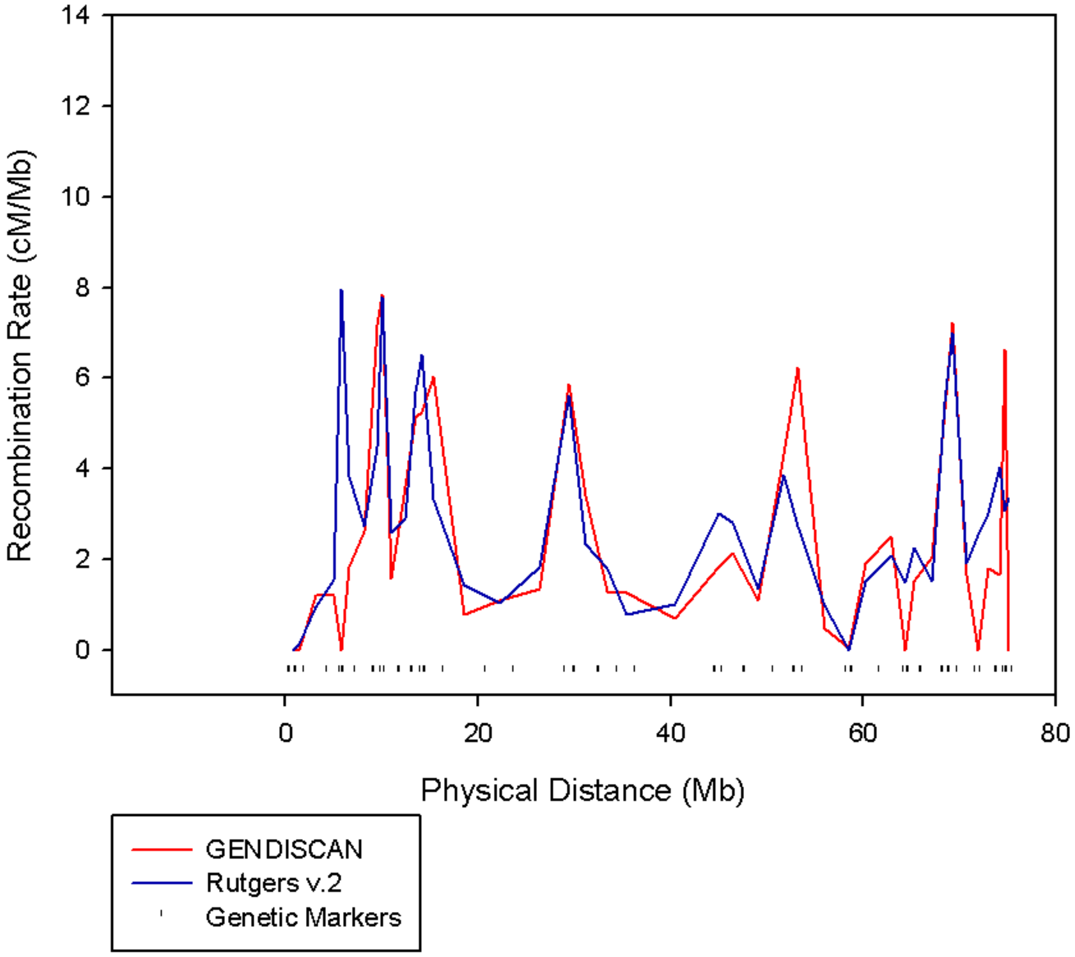

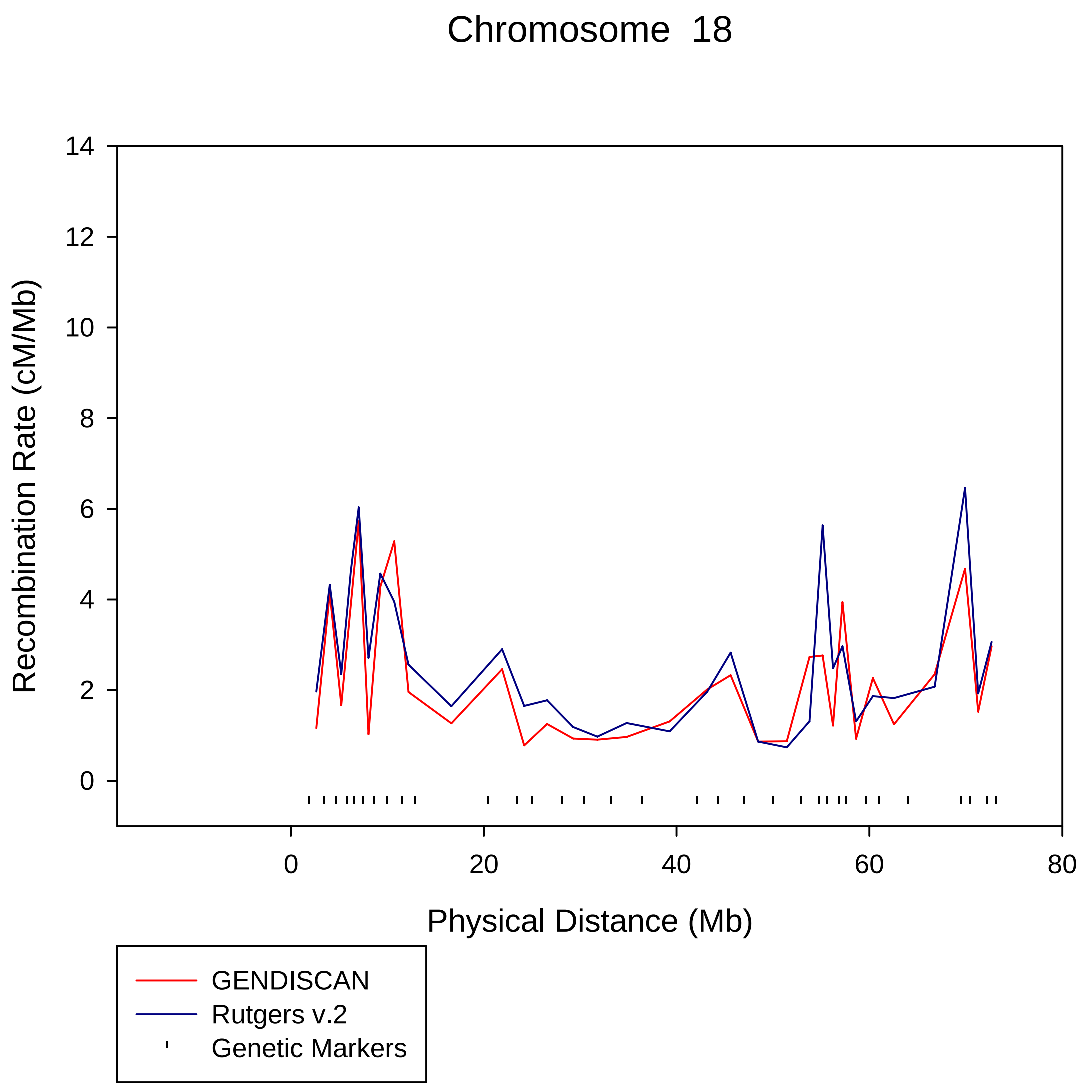

Chromosome 18
Recombination Rate (cM/Mb)
14
12
10
8
6
4
2
0
0
20
40
60
80
Physical Distance (Mb)
GENDISCAN
Rutgers v.2
Genetic Markers

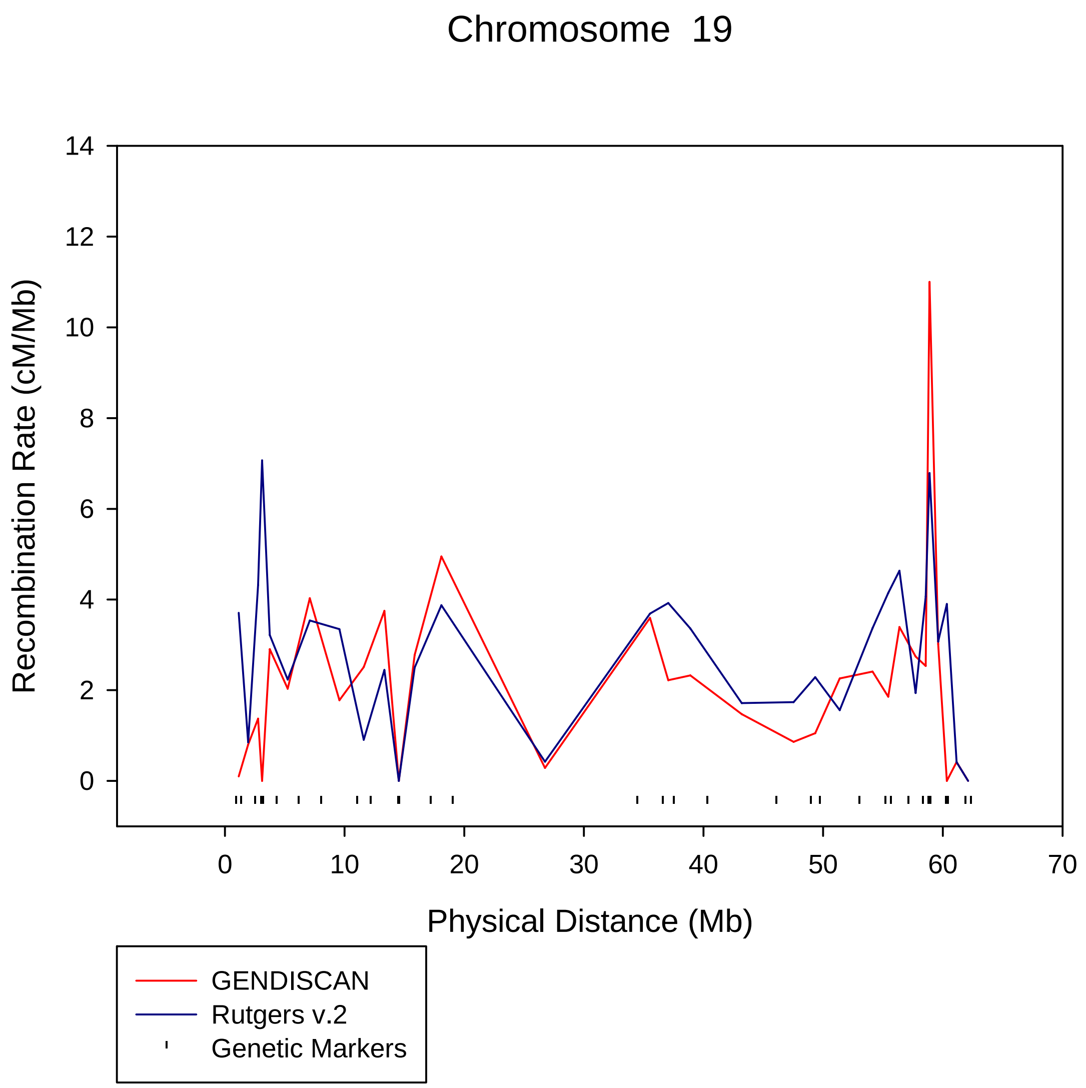
Chromosome 19
Recombination Rate (cM/Mb)
0
2
4
6
8
10
12
14
0
10
20
30
40
50
60
70
Physical Distance (Mb)
GENDISCAN
Rutgers v.2
Genetic Markers

Chromosome 20

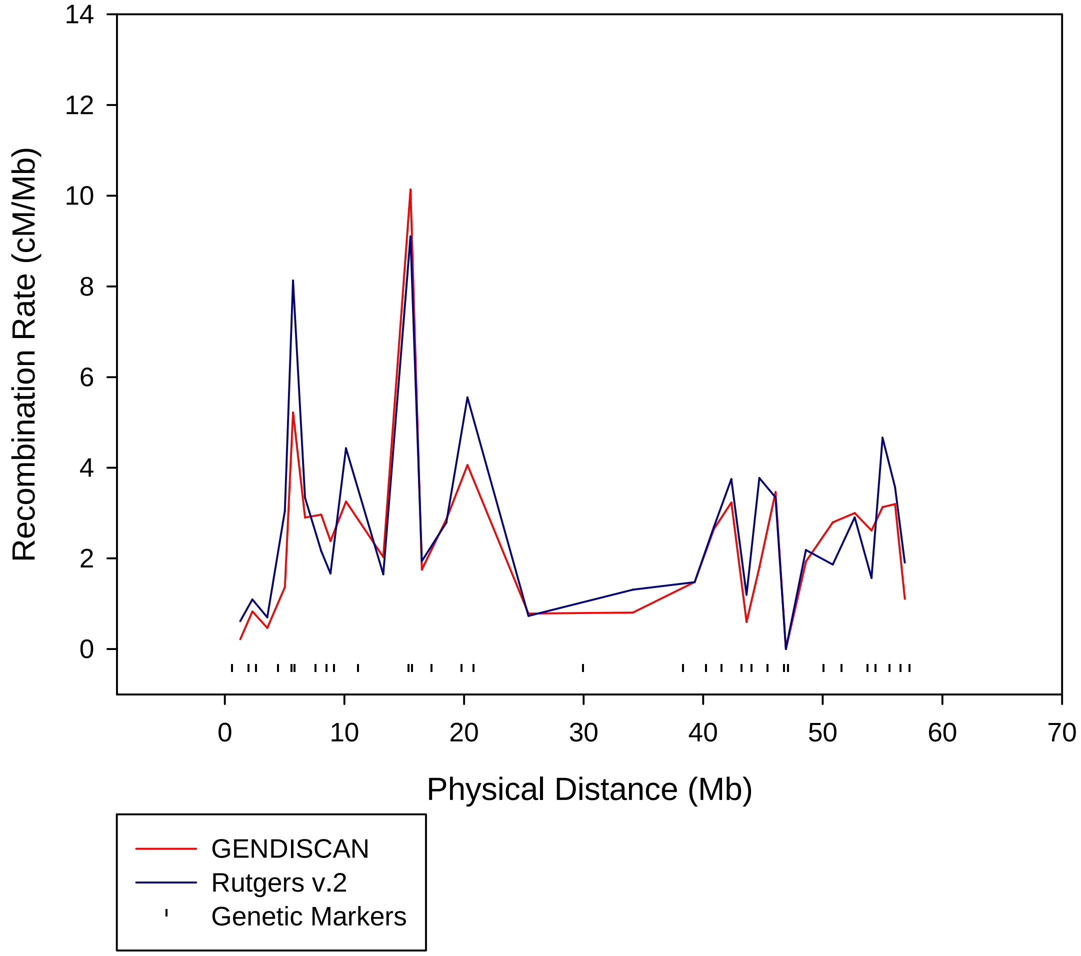

Chromosome 21

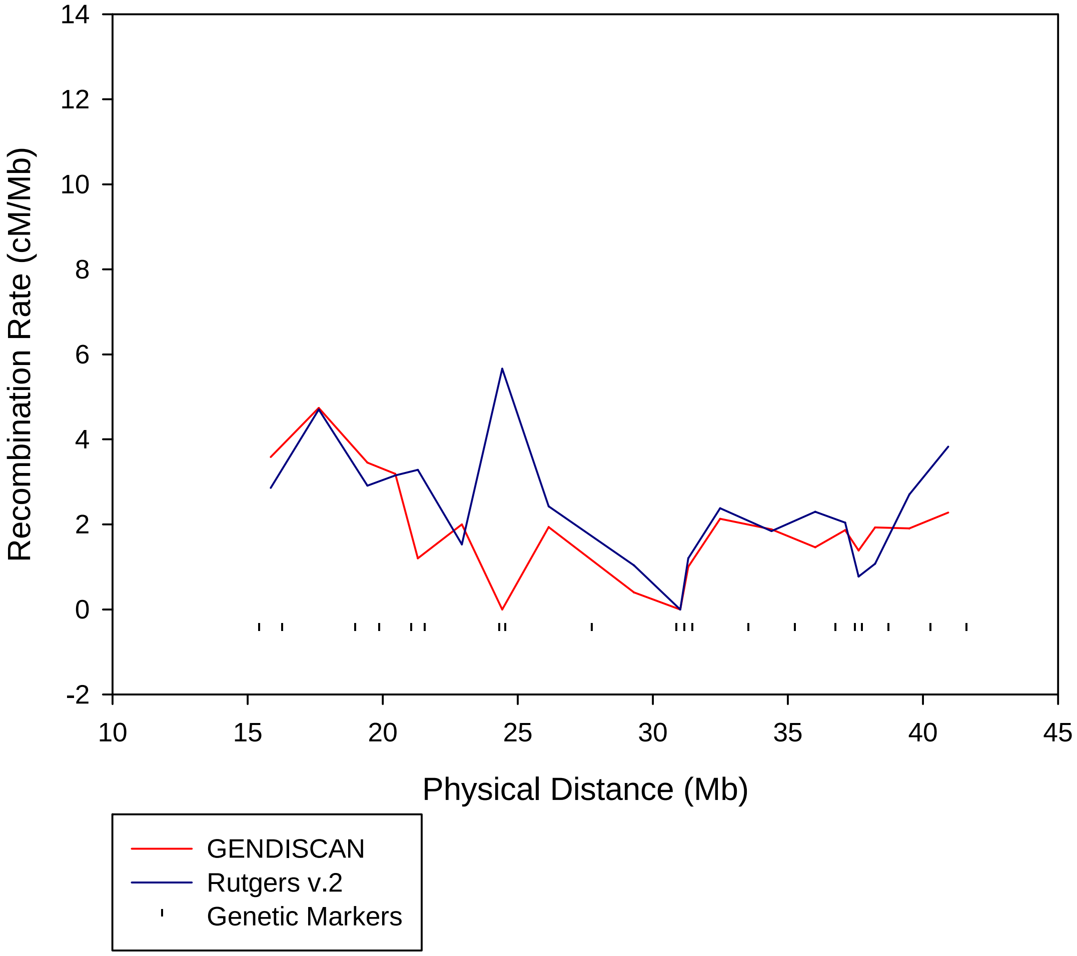

Chromosome 22

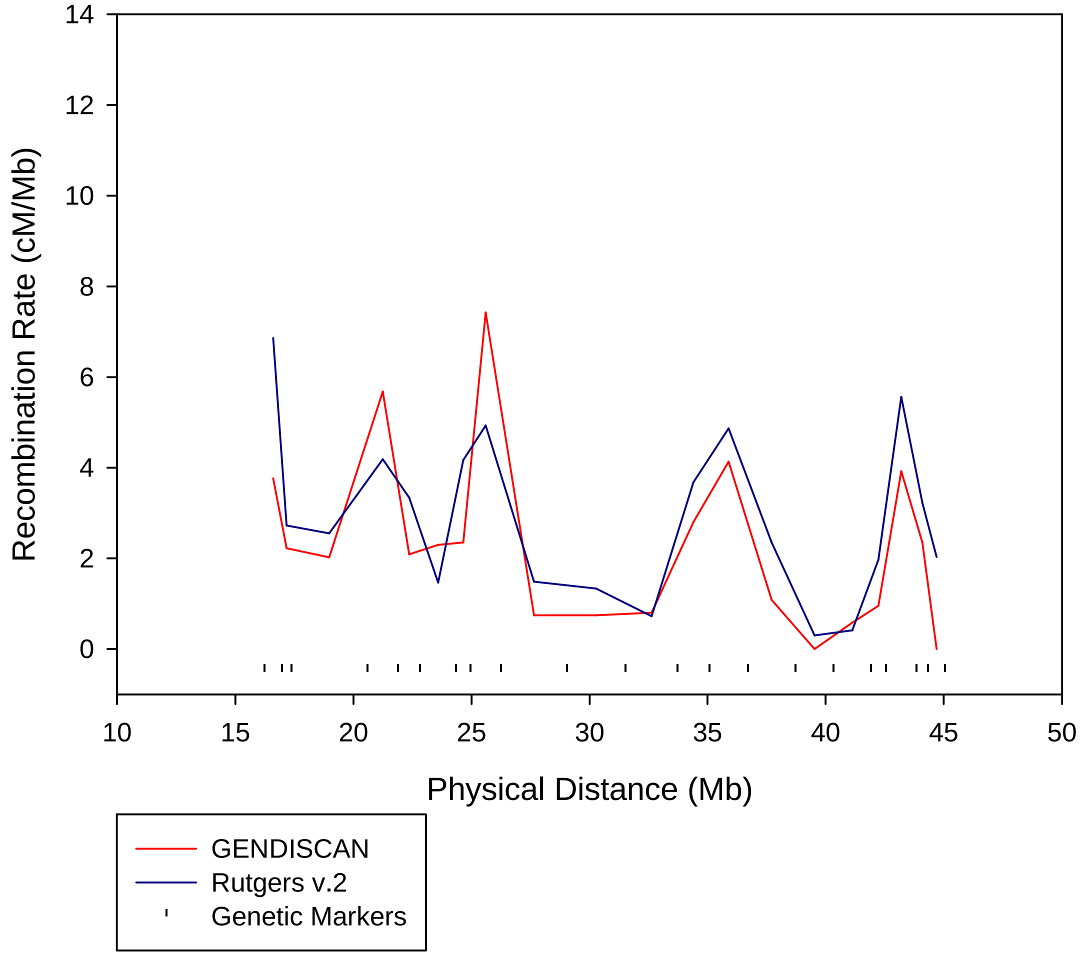

Supplement: Additional file 3 — Recombination rates of female map. Figures of recombination rate patterns of all autosomes of the GENDISCAN and Rutgers v.2 female maps. [file 1471-2164-9-554-S3.pdf]
