# Supplementary material for: A genome-wide Asian genetic map and ethnic comparison: The GENDISCAN study
Source: BMC Genomics. 2008 Nov 25;9:554. doi: 10.1186/1471-2164-9-554 (PMC2612022; doi:10.1186/1471-2164-9-554)
Supplement: Additional file 4 — Recombination rates of male map. Figures of recombination rate patterns of all autosomes of the GENDISCAN and Rutgers v.2 male maps. [file 1471-2164-9-554-S4.pdf]

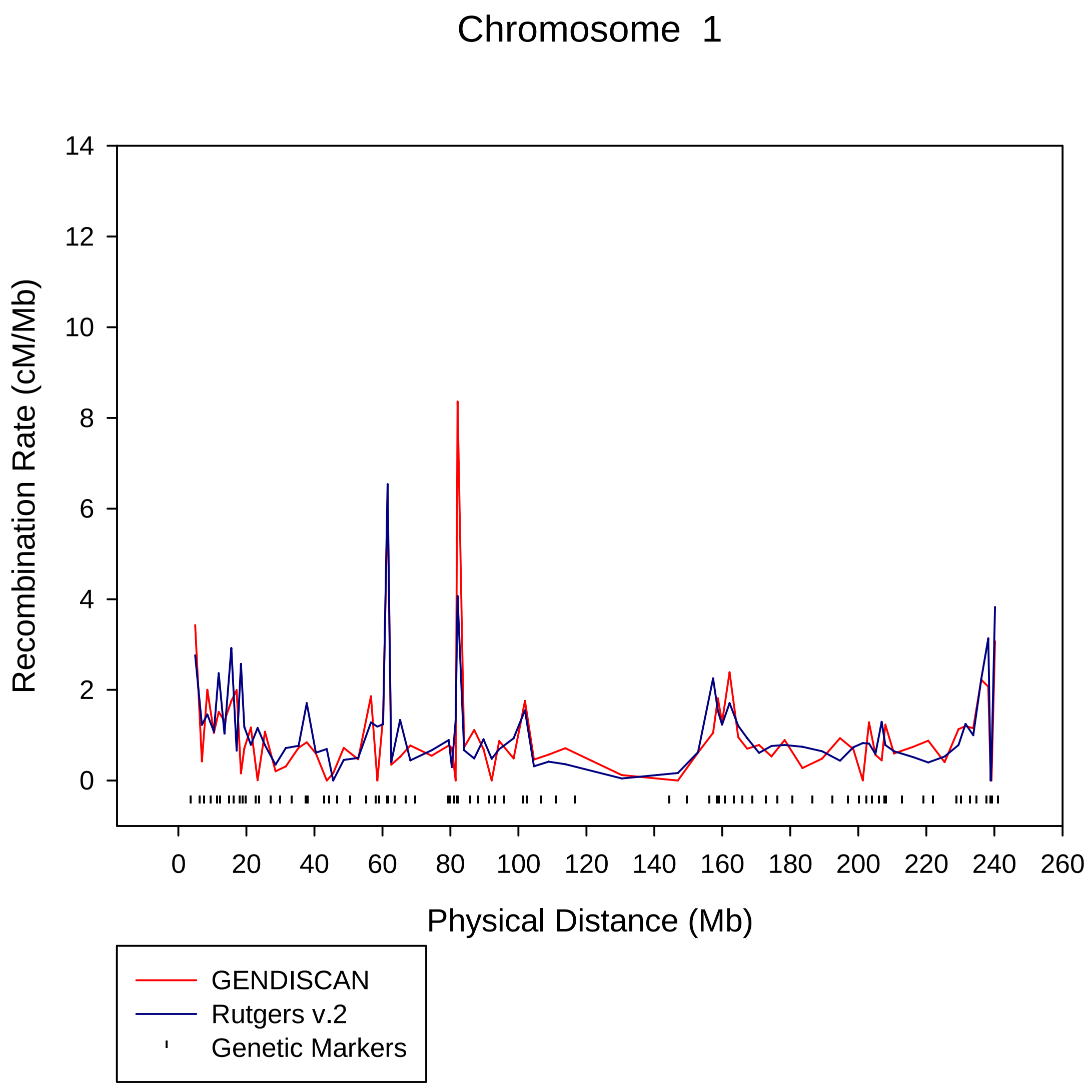
Chromosome 1
Recombination Rate (cM/Mb)
14
12
10
8
6
4
2
0
0
20
40
60
80
100
120
140
160
180
200
220
240
260
Physical Distance (Mb)
GENDISCAN
Rutgers v.2
Genetic Markers

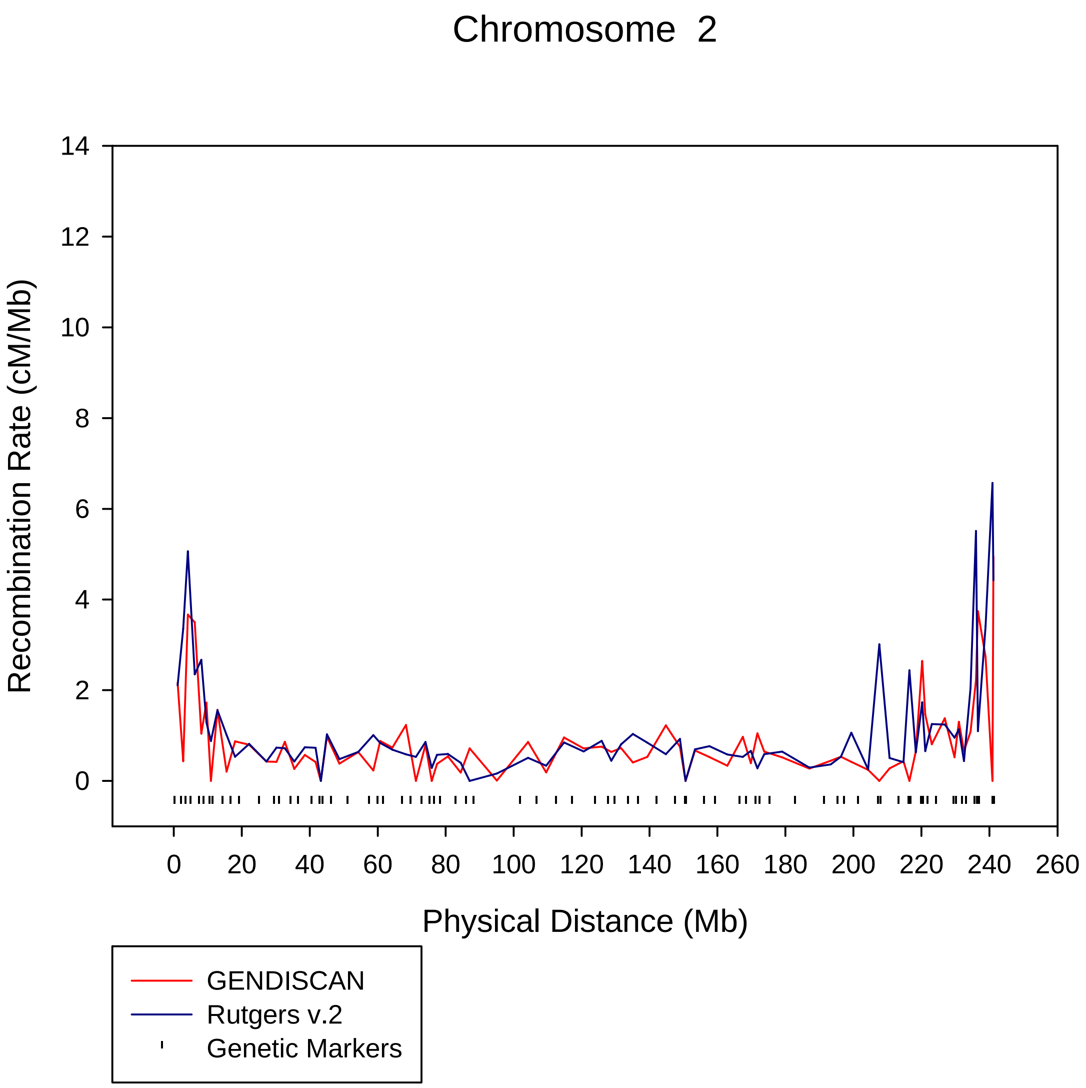

Chromosome 2
Recombination Rate (cM/Mb)
0
2
4
6
8
10
12
14
0
20
40
60
80
100
120
140
160
180
200
220
240
260
Physical Distance (Mb)
GENDISCAN
Rutgers v.2
Genetic Markers

Chromosome 3

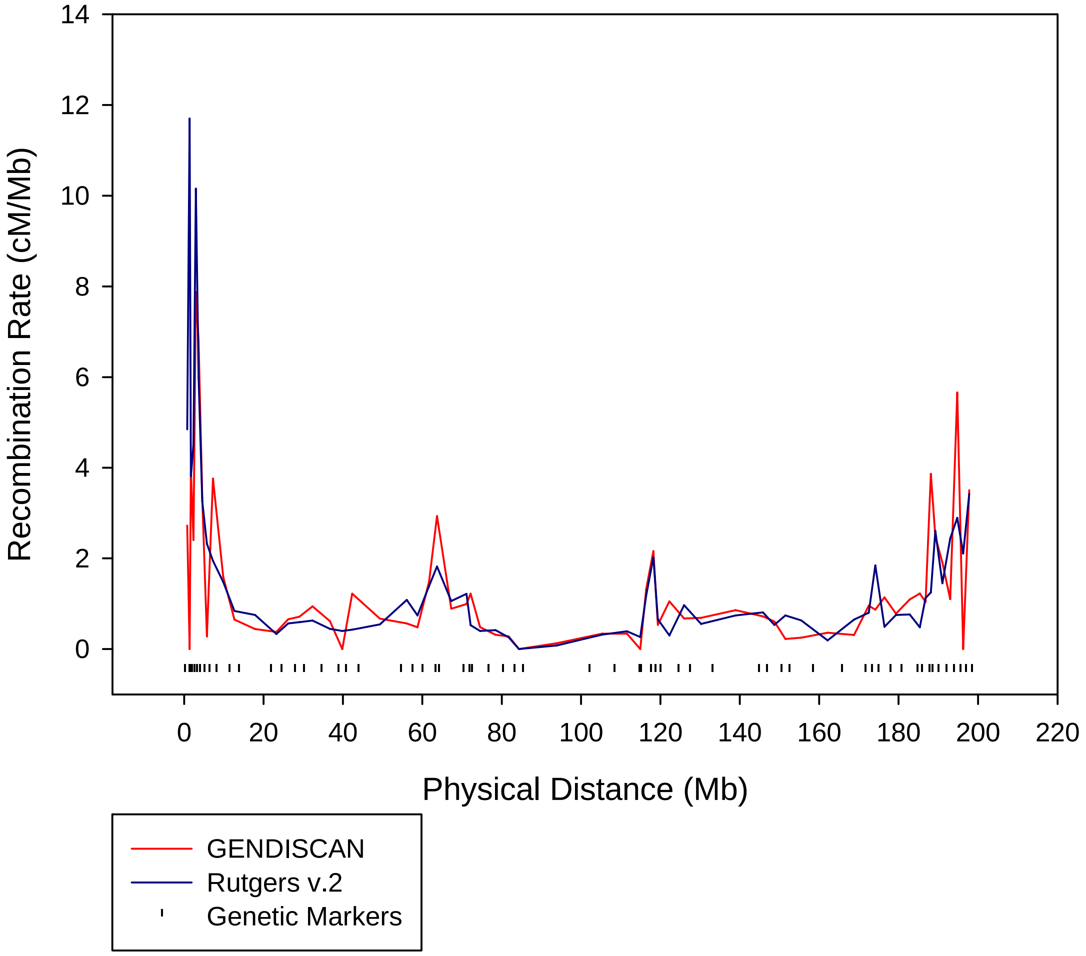

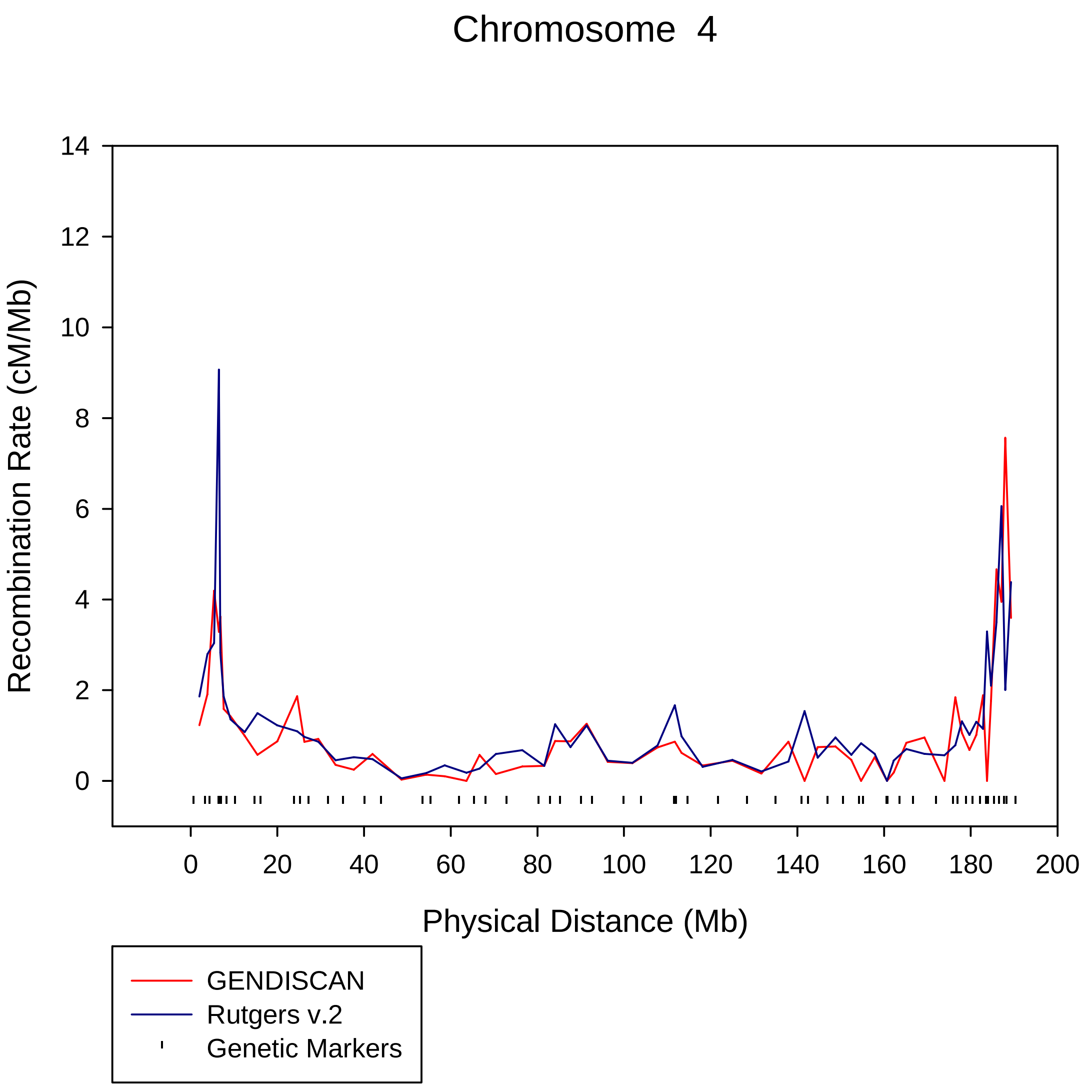
Chromosome 4
Recombination Rate (cM/Mb)
0
2
4
6
8
10
12
14
0
20
40
60
80
100
120
140
160
180
200
Physical Distance (Mb)
GENDISCAN
Rutgers v.2
Genetic Markers

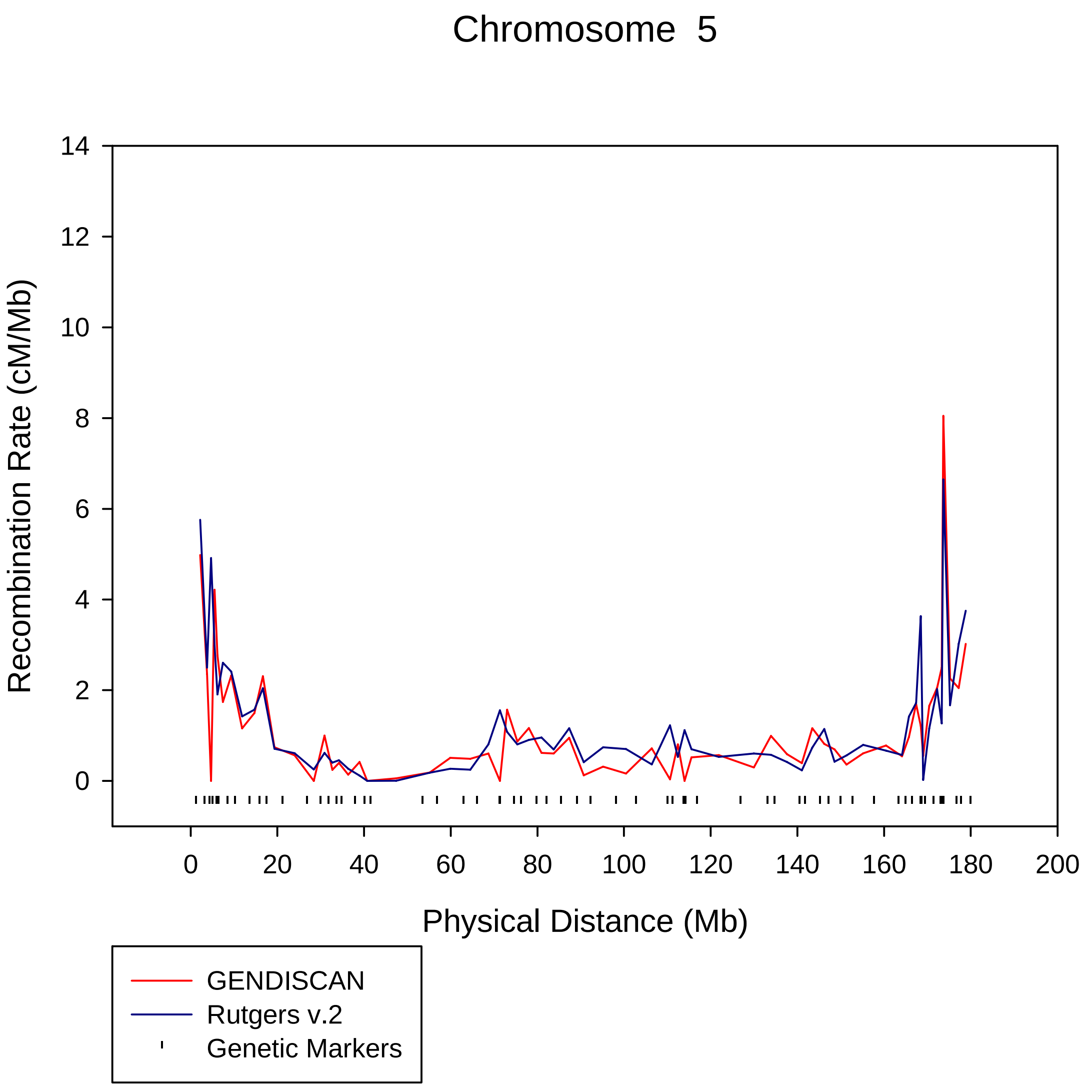

Chromosome 5
Recombination Rate (cM/Mb)
14
12
10
8
6
4
2
0
0
20
40
60
80
100
120
140
160
180
200
Physical Distance (Mb)
GENDISCAN
Rutgers v.2
Genetic Markers

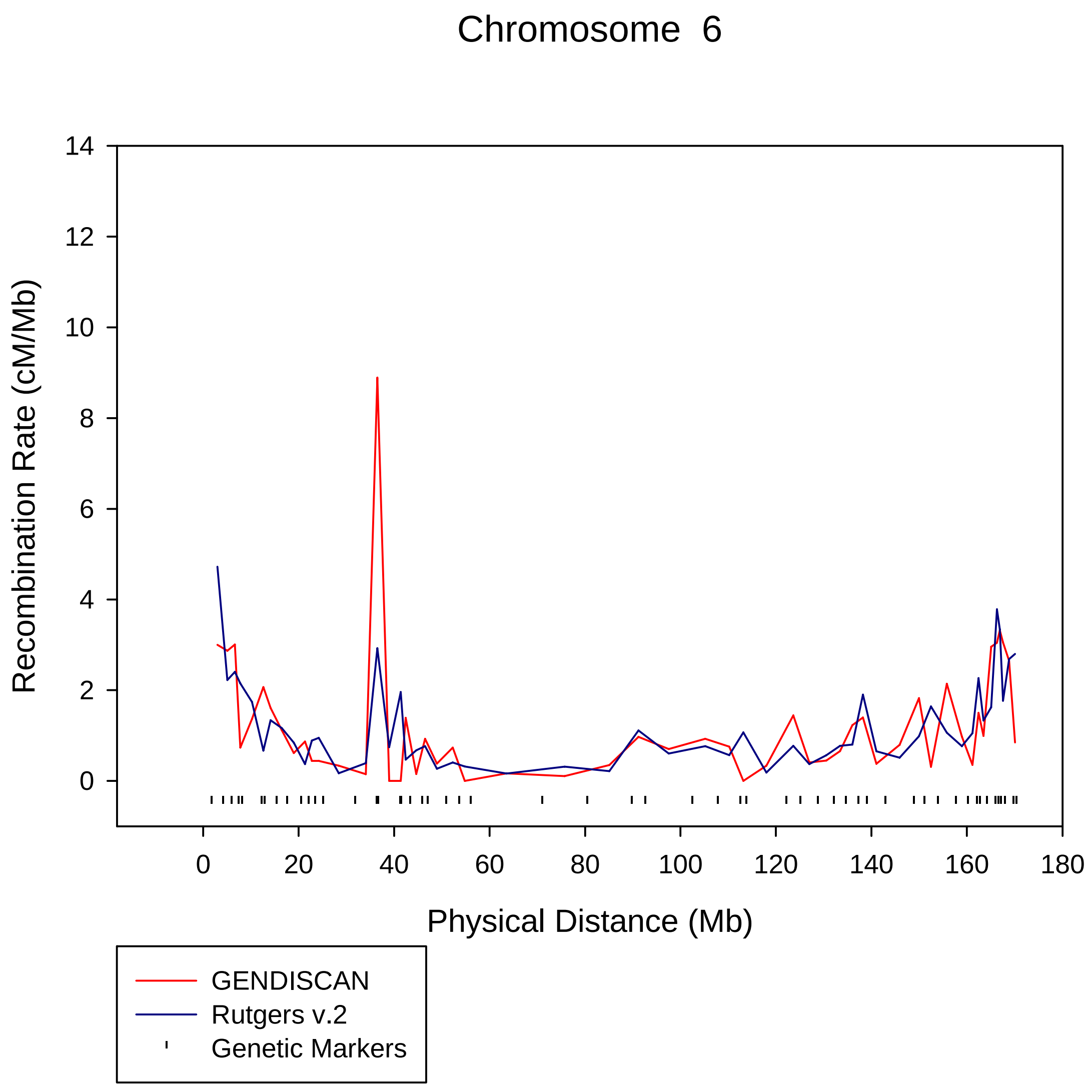

Chromosome 6
Recombination Rate (cM/Mb)
0
2
4
6
8
10
12
14
0
20
40
60
80
100
120
140
160
180
Physical Distance (Mb)
GENDISCAN
Rutgers v.2
Genetic Markers

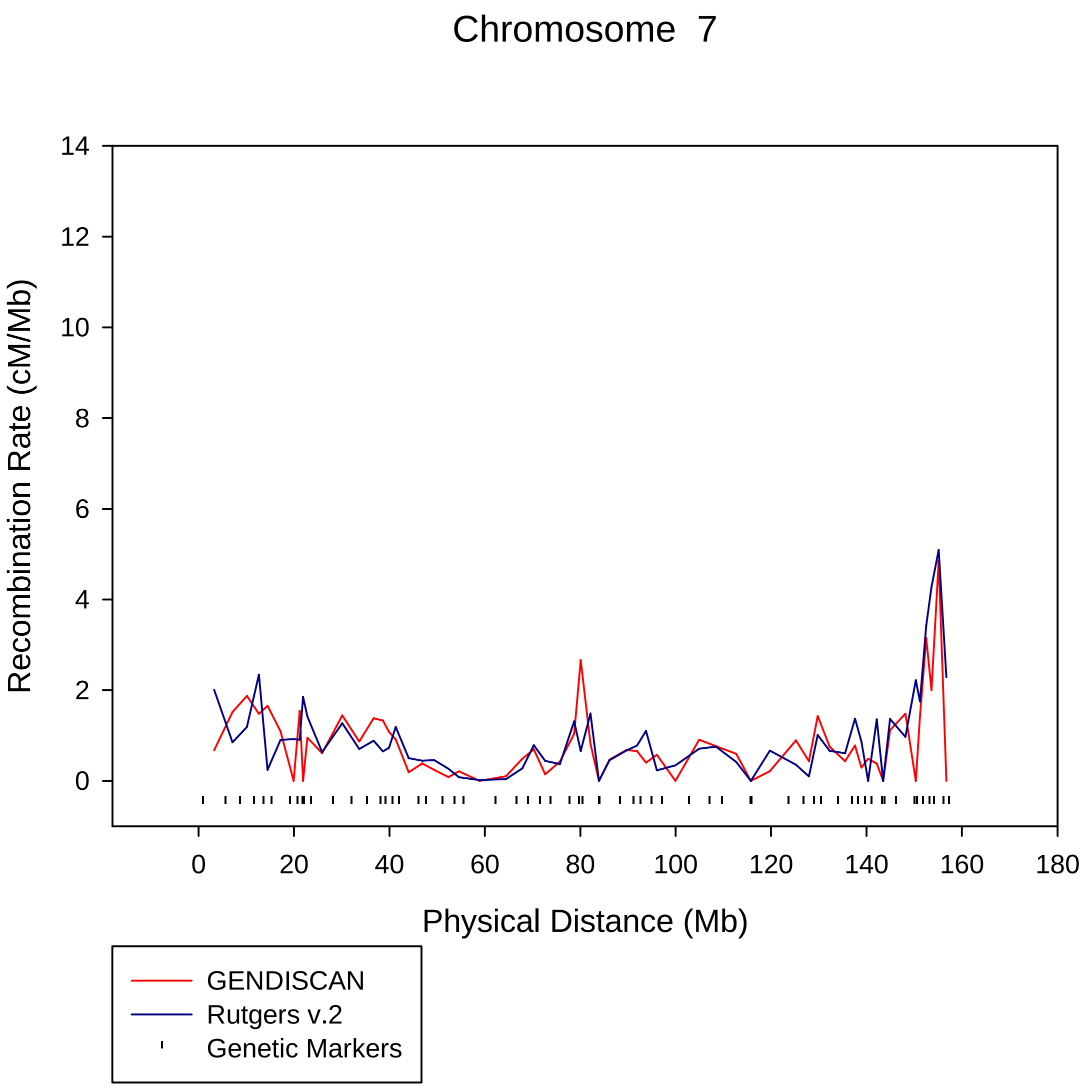
Chromosome 7
Recombination Rate (cM/Mb)
14
12
10
8
6
4
2
0
0
20
40
60
80
100
120
140
160
180
Physical Distance (Mb)
GENDISCAN
Rutgers v.2
Genetic Markers

Chromosome 8

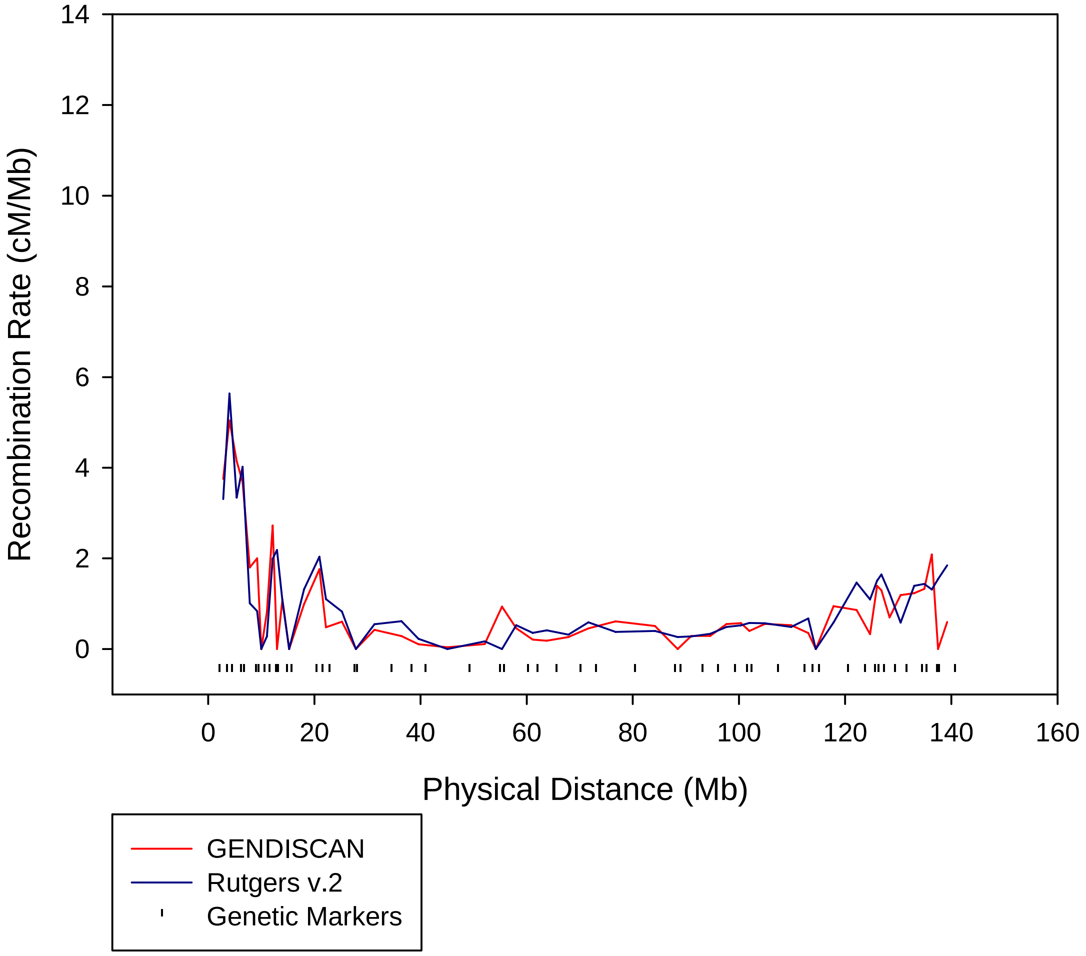

Chromosome 9

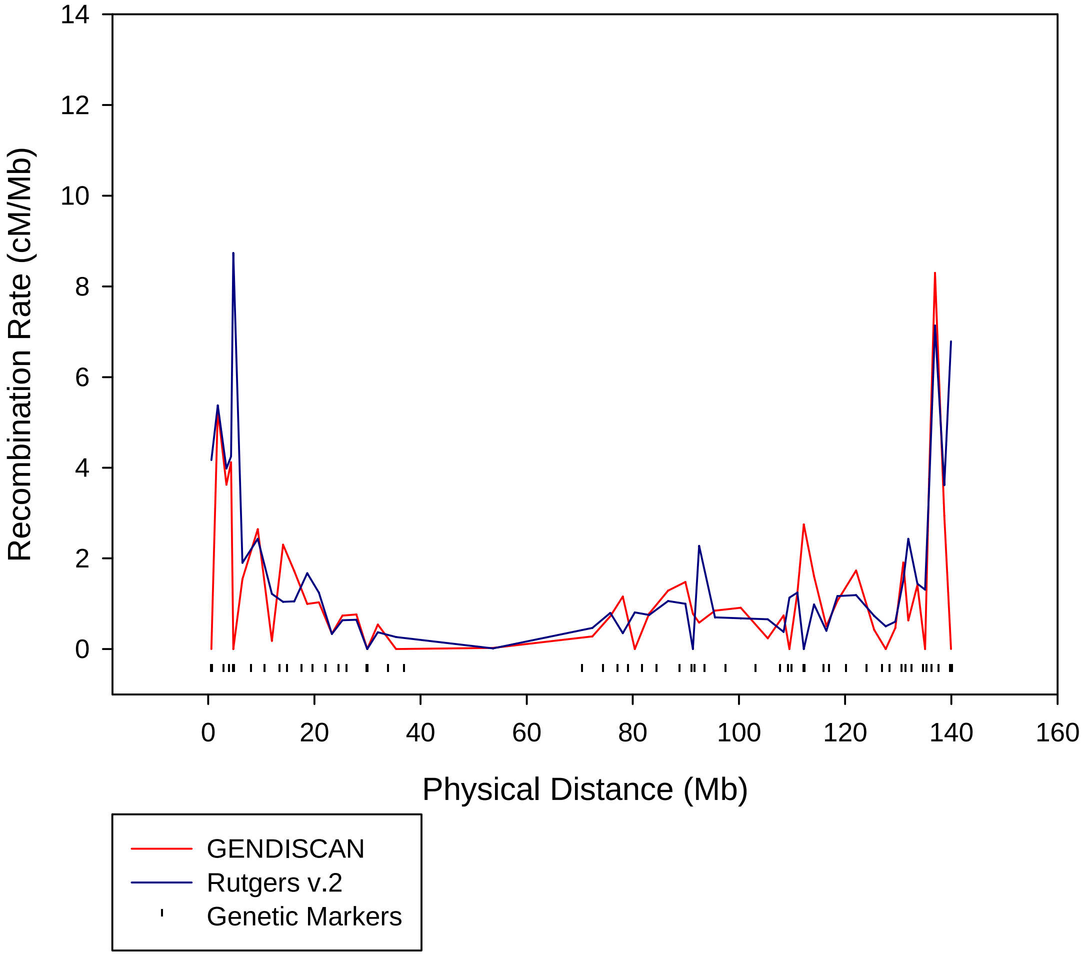

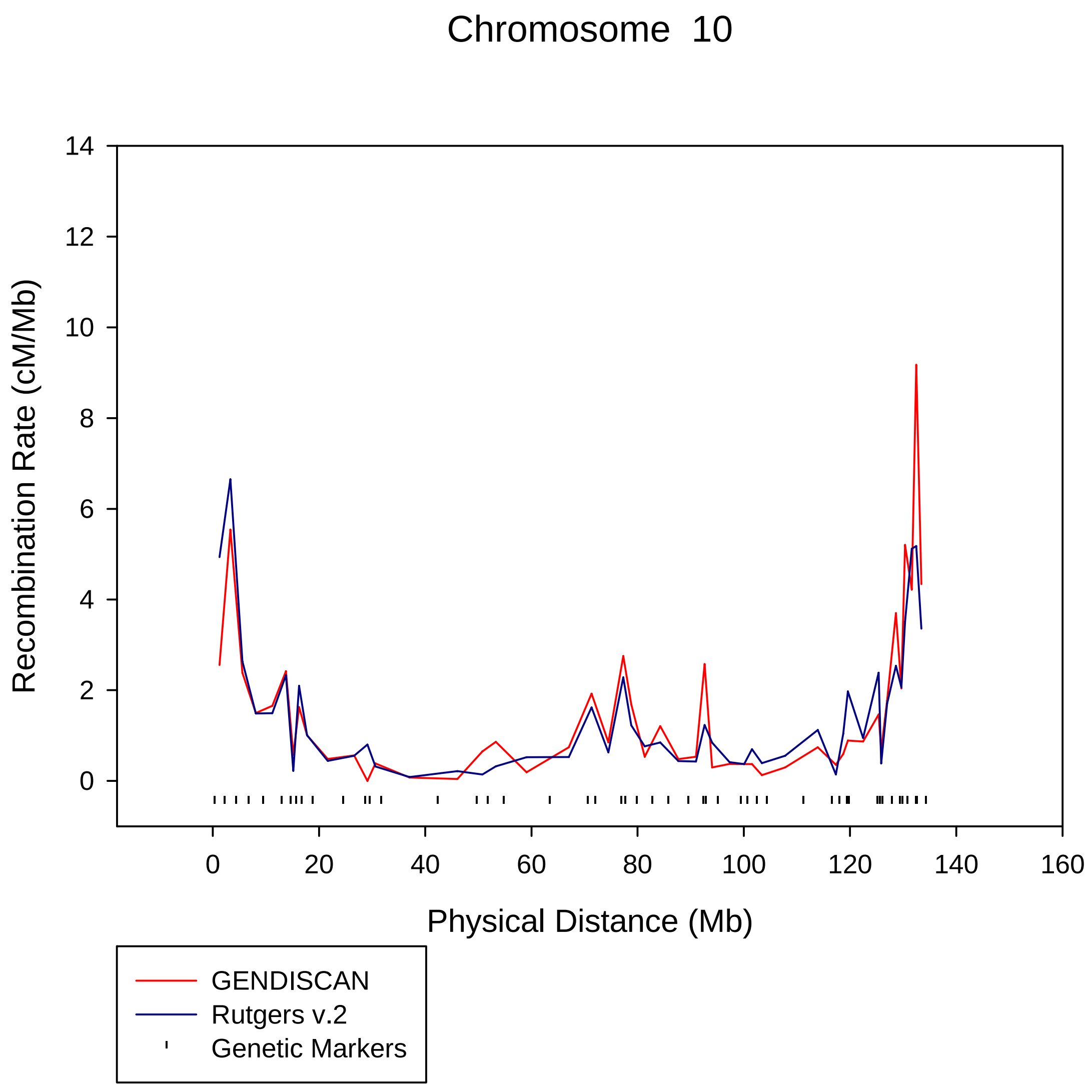
Chromosome 10
Recombination Rate (cM/Mb)
0
2
4
6
8
10
12
14
0
20
40
60
80
100
120
140
160
Physical Distance (Mb)
GENDISCAN
Rutgers v.2
Genetic Markers

Chromosome 11

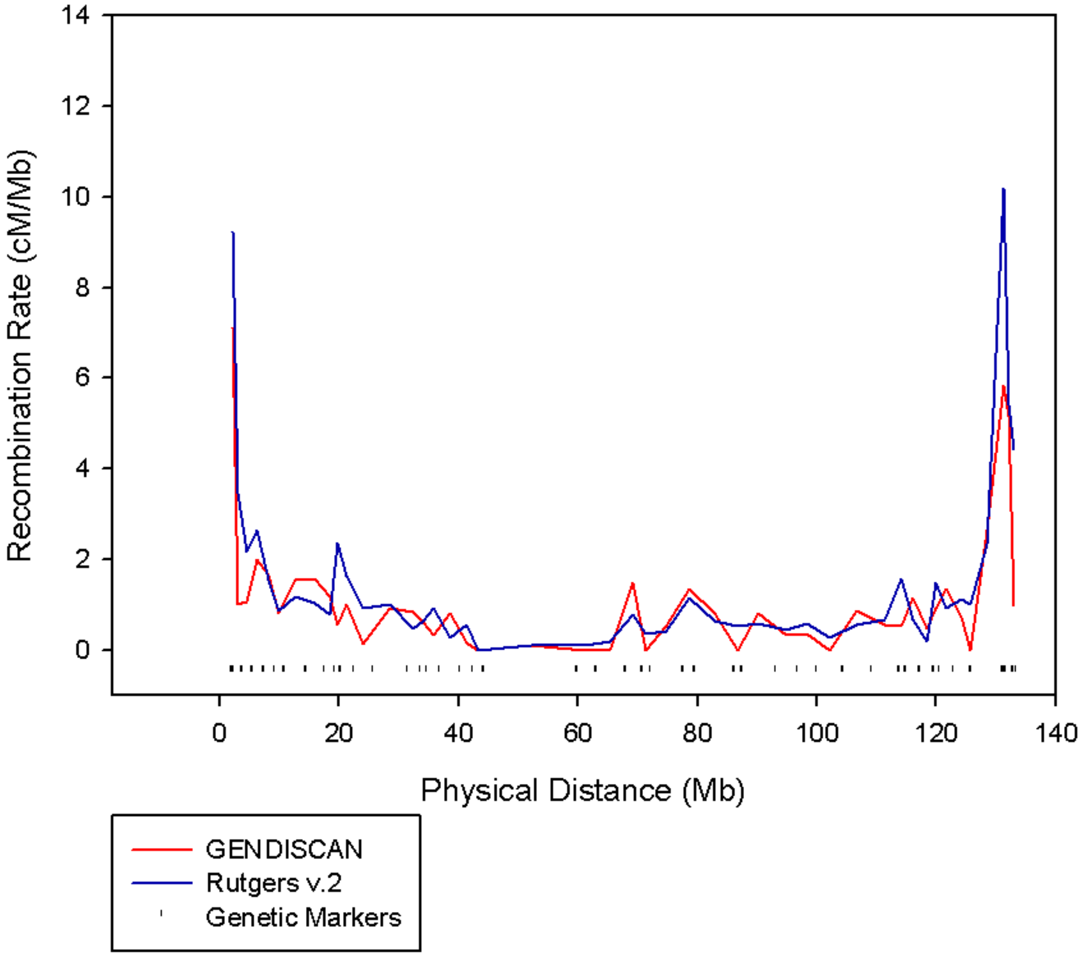

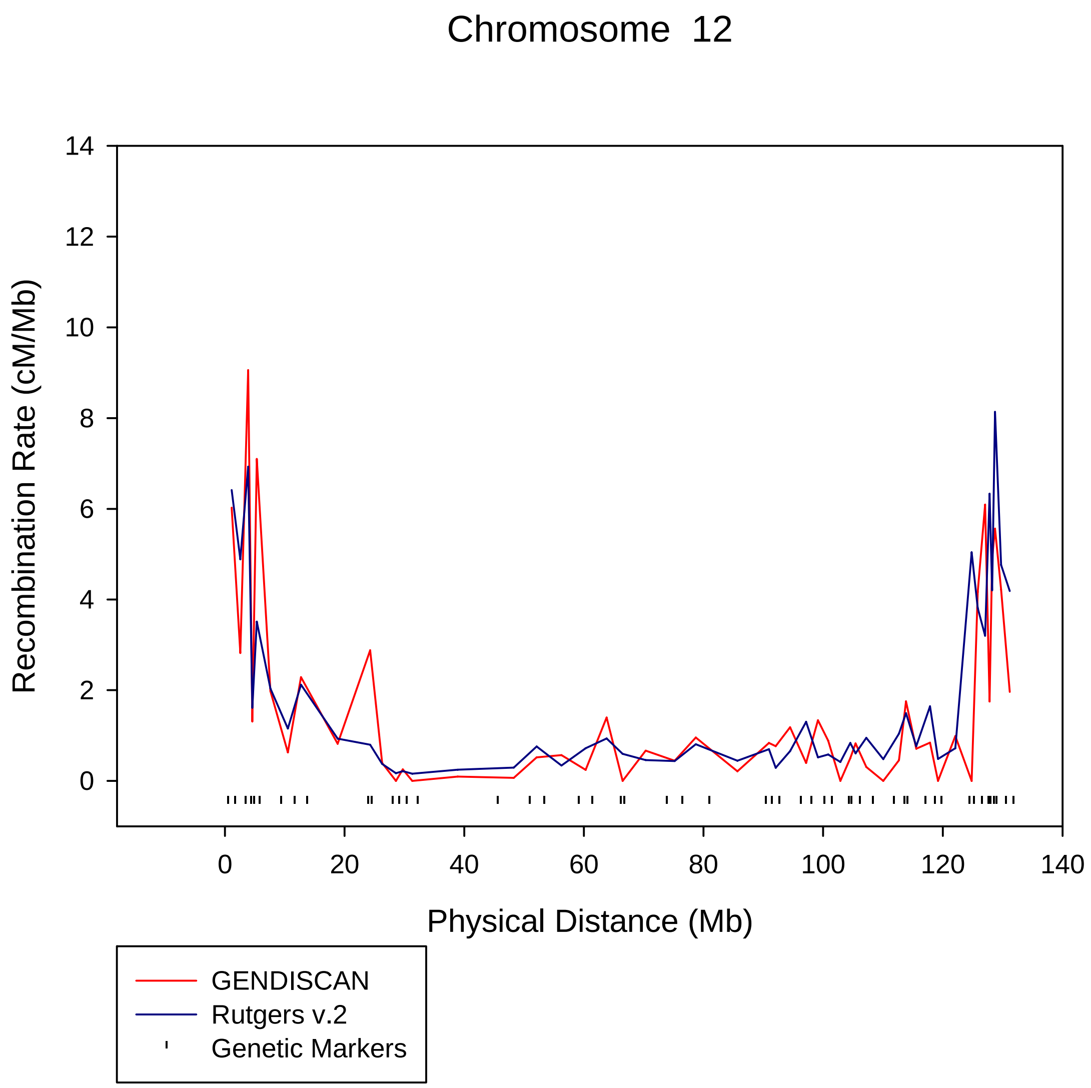
Chromosome 12
Recombination Rate (cM/Mb)
0
2
4
6
8
10
12
14
0
20
40
60
80
100
120
140
Physical Distance (Mb)
GENDISCAN
Rutgers v.2
Genetic Markers

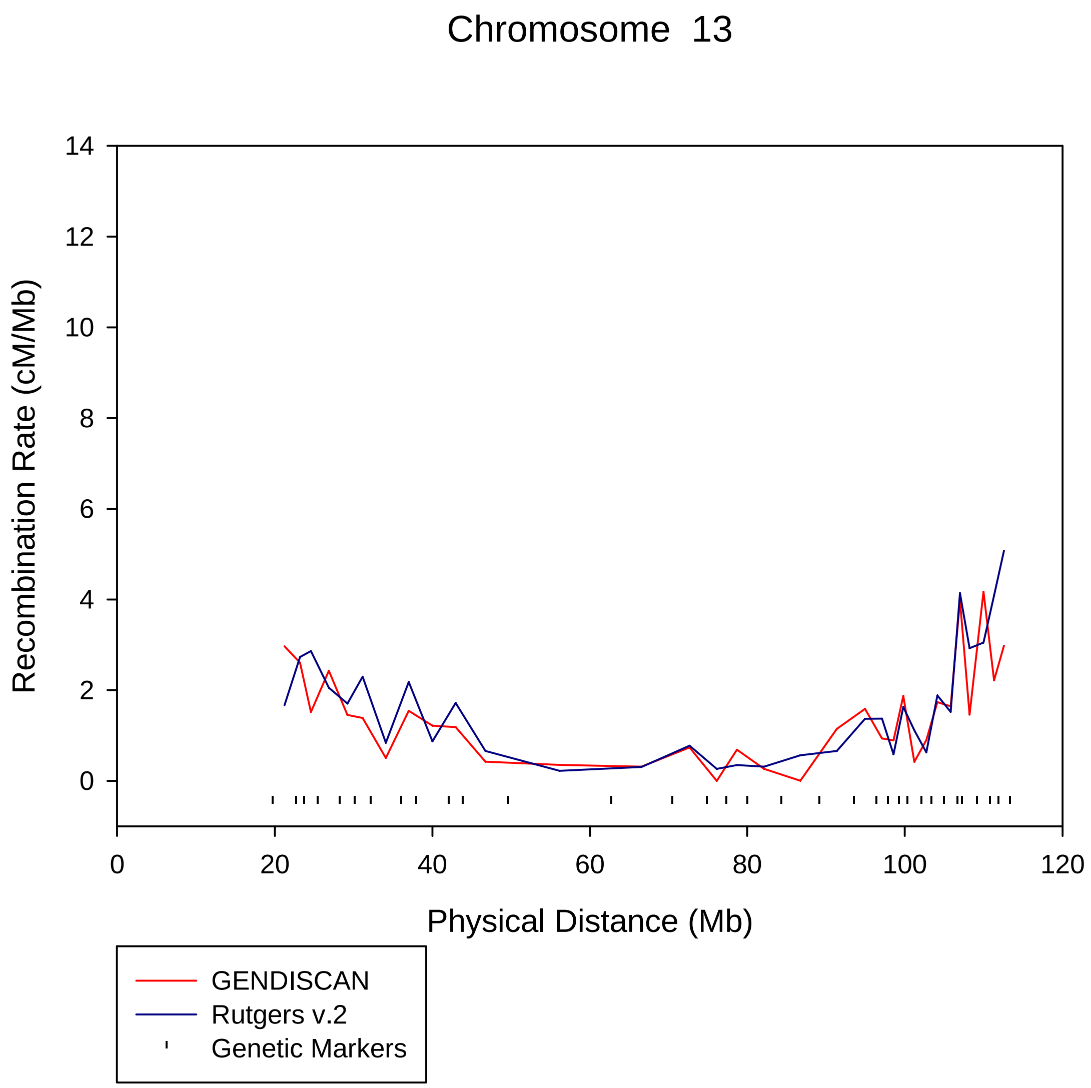

Chromosome 13
Recombination Rate (cM/Mb)
14
12
10
8
6
4
2
0
0
20
40
60
80
100
120
Physical Distance (Mb)
GENDISCAN
Rutgers v.2
Genetic Markers

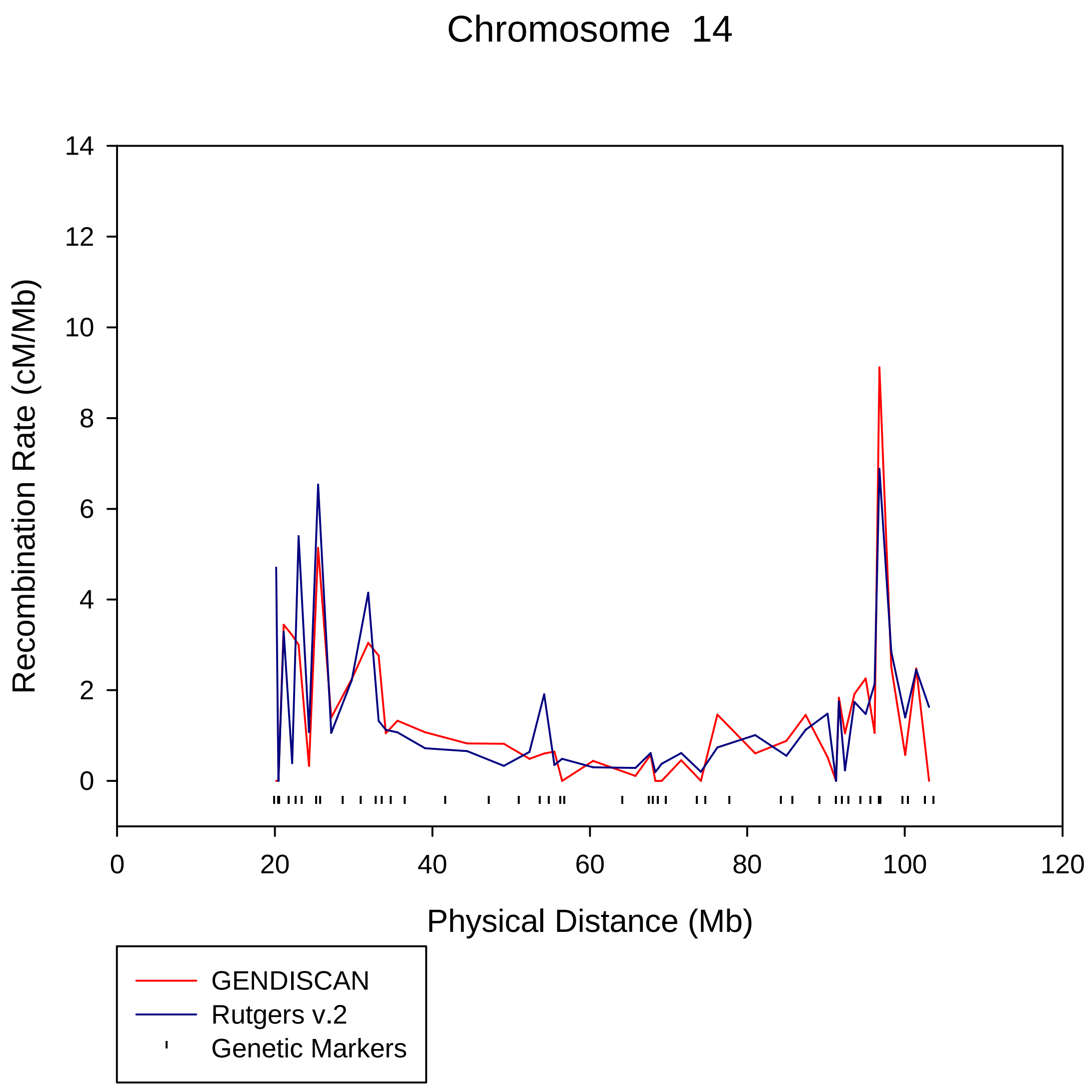
Chromosome 14
Recombination Rate (cM/Mb)
14
12
10
8
6
4
2
0
0
20
40
60
80
100
120
Physical Distance (Mb)
GENDISCAN
Rutgers v.2
Genetic Markers

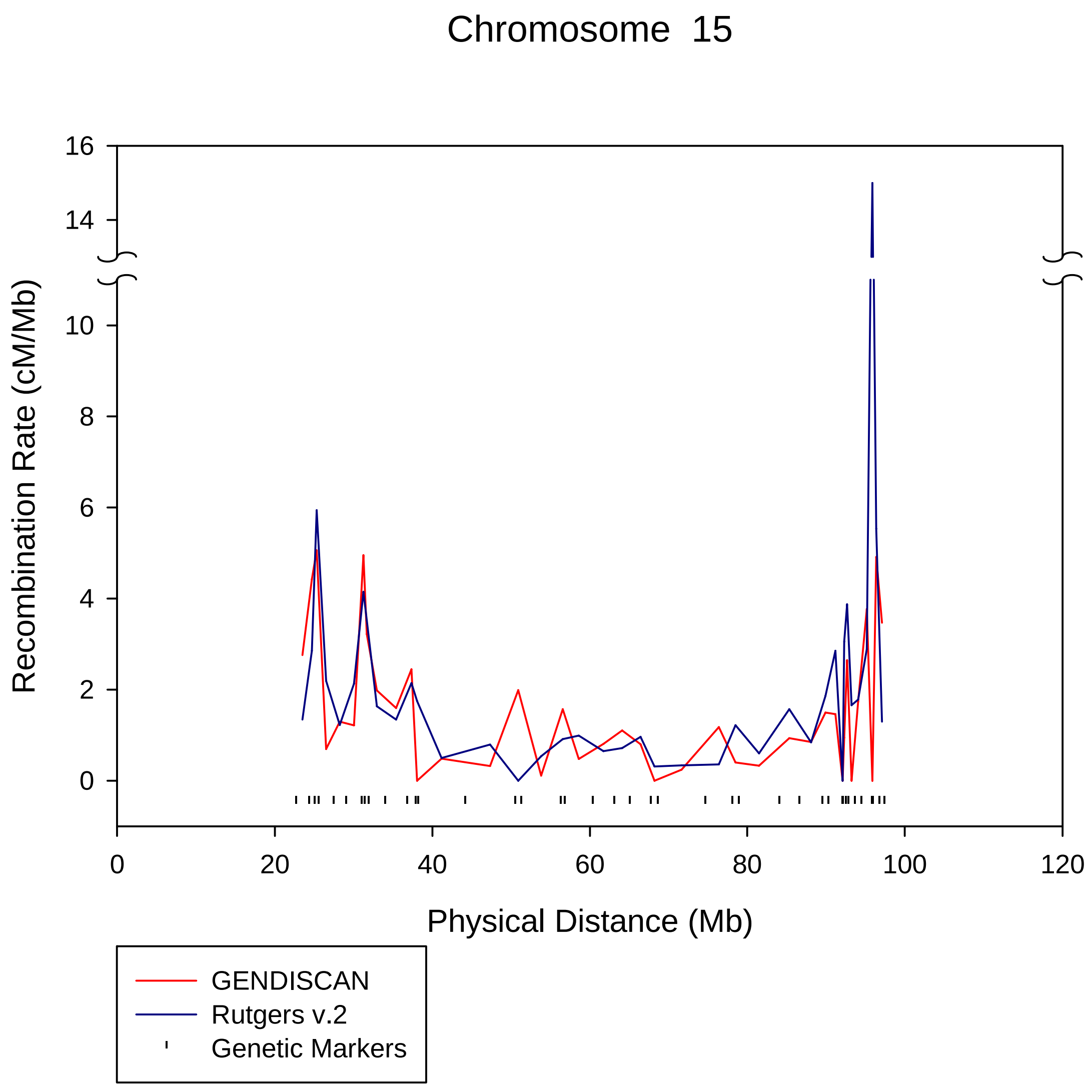
Chromosome 15
Recombination Rate (cM/Mb)
0
2
4
6
8
10
14
16
0
20
40
60
80
100
120
Physical Distance (Mb)
GENDISCAN
Rutgers v.2
Genetic Markers

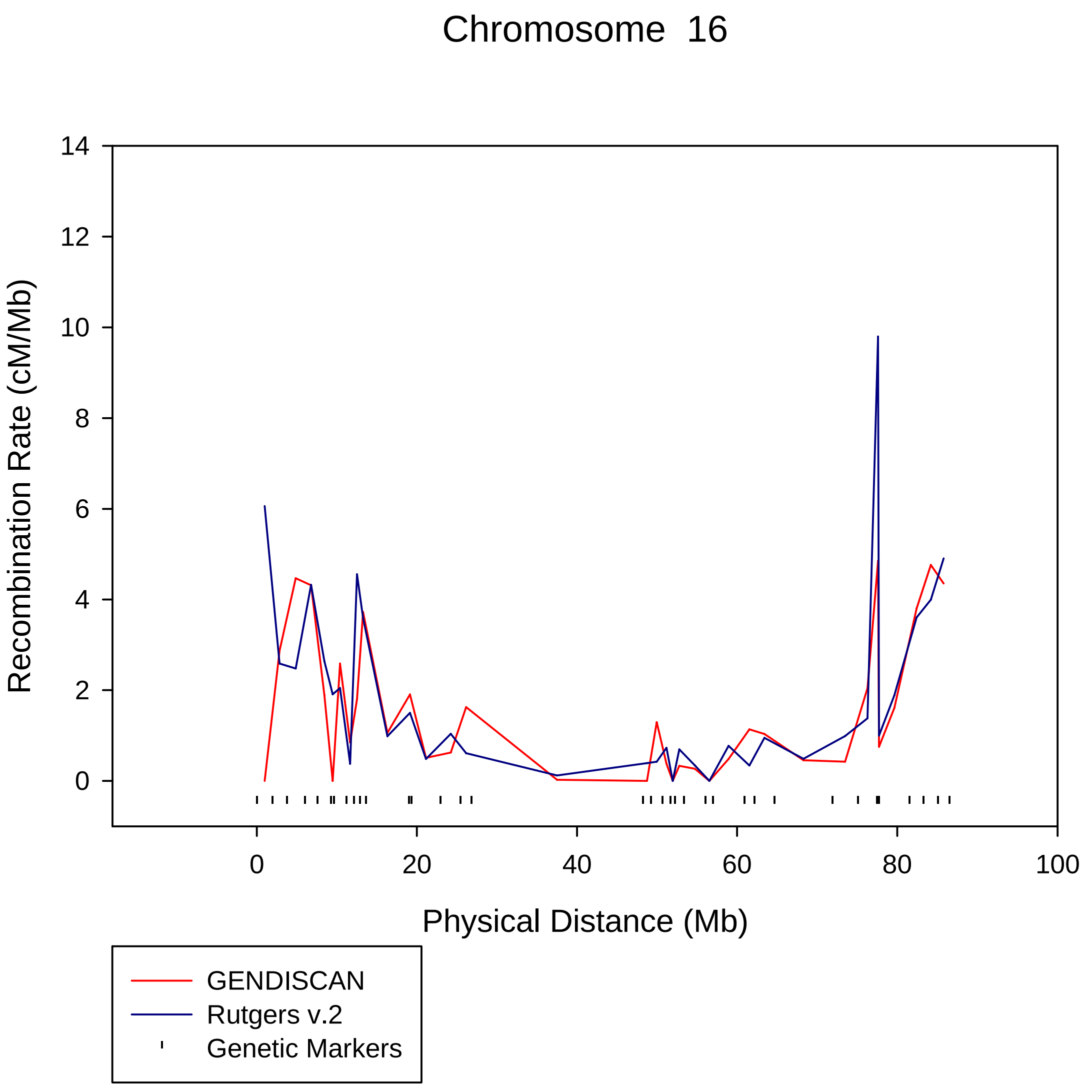
Chromosome 16
Recombination Rate (cM/Mb)
0
2
4
6
8
10
12
14
0
20
40
60
80
100
Physical Distance (Mb)
GENDISCAN
Rutgers v.2
Genetic Markers

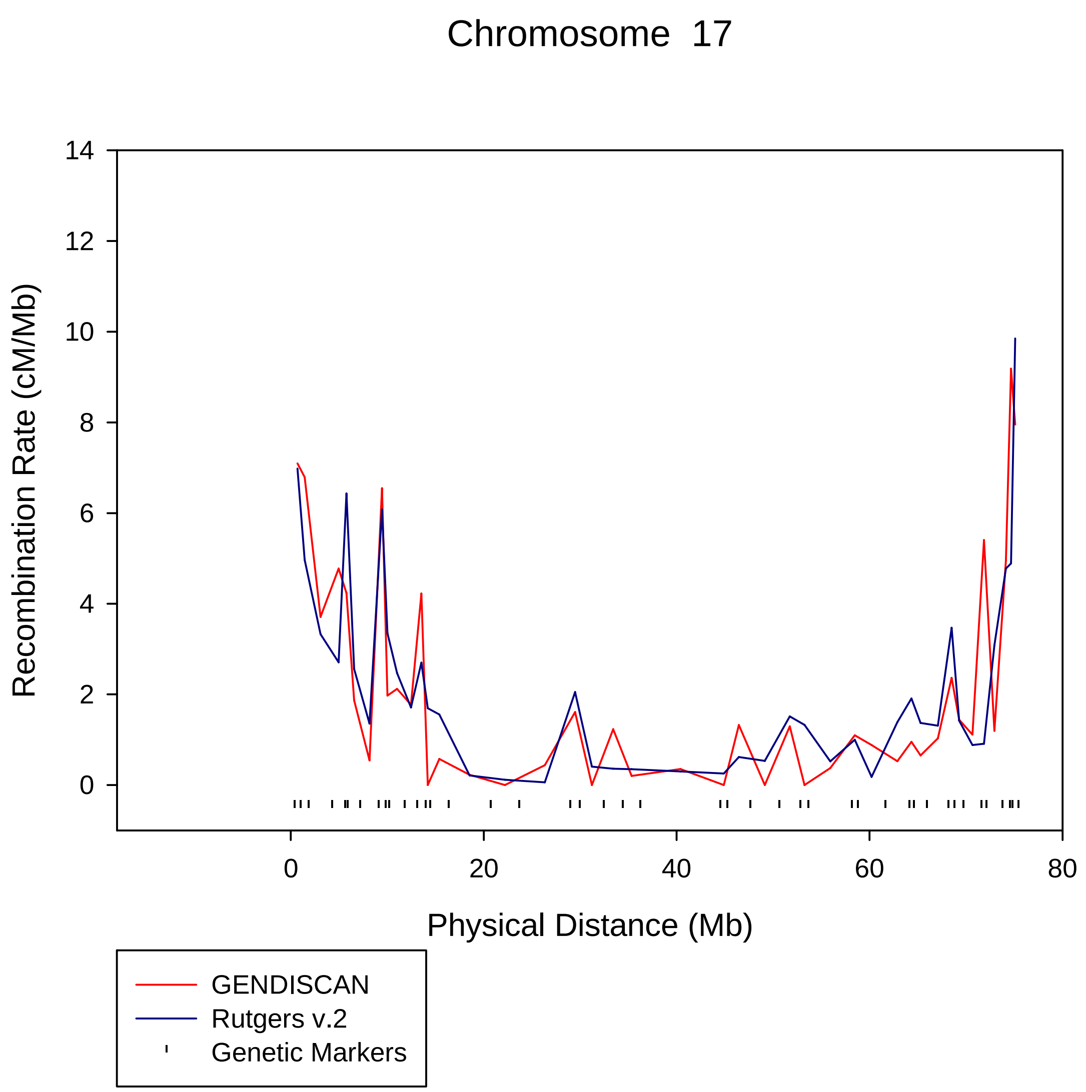

Chromosome 17
Recombination Rate (cM/Mb)
14
12
10
8
6
4
2
0
0
20
40
60
80
Physical Distance (Mb)
GENDISCAN
Rutgers v.2
Genetic Markers

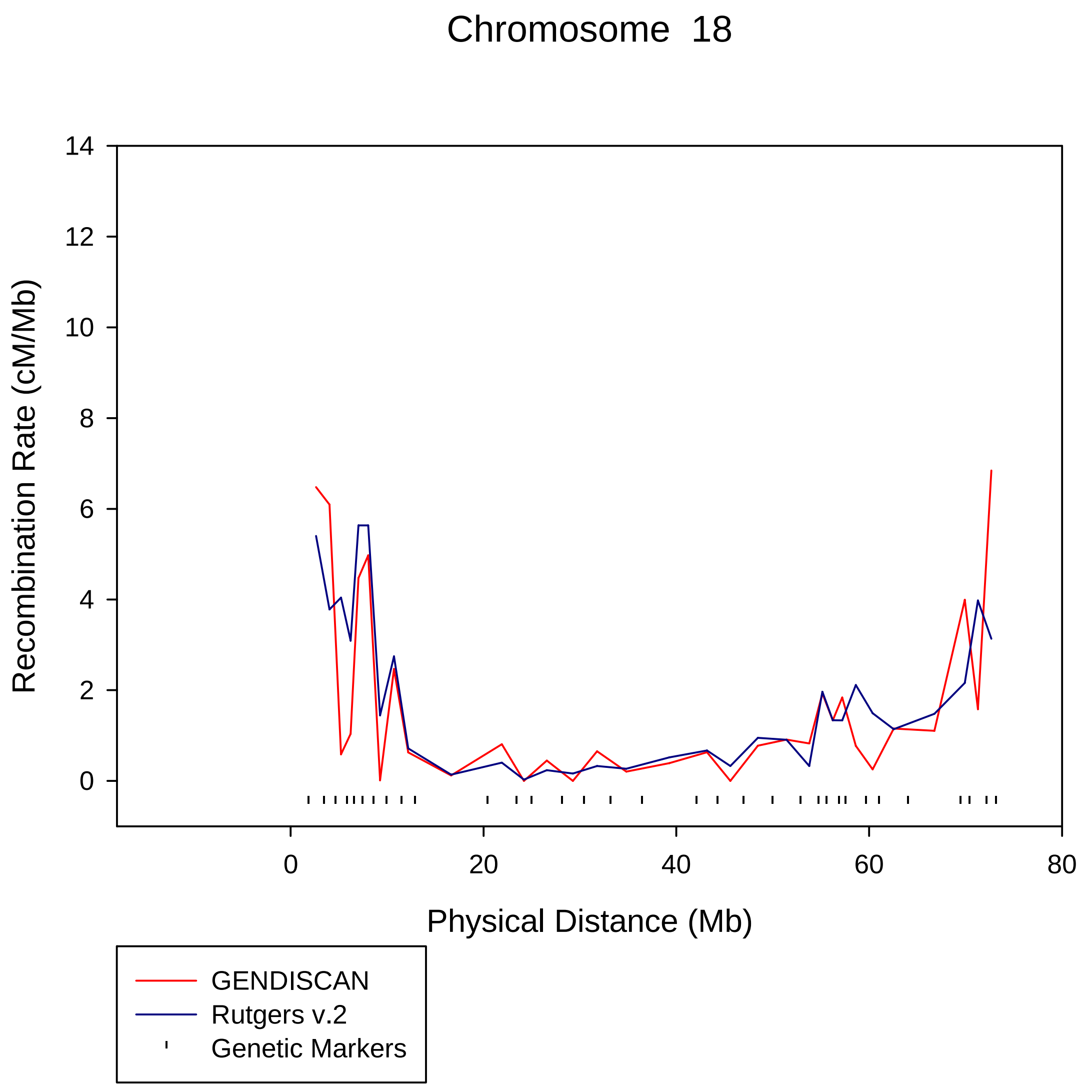
Chromosome 18
Recombination Rate (cM/Mb)
14
12
10
8
6
4
2
0
0
20
40
60
80
Physical Distance (Mb)
GENDISCAN
Rutgers v.2
Genetic Markers

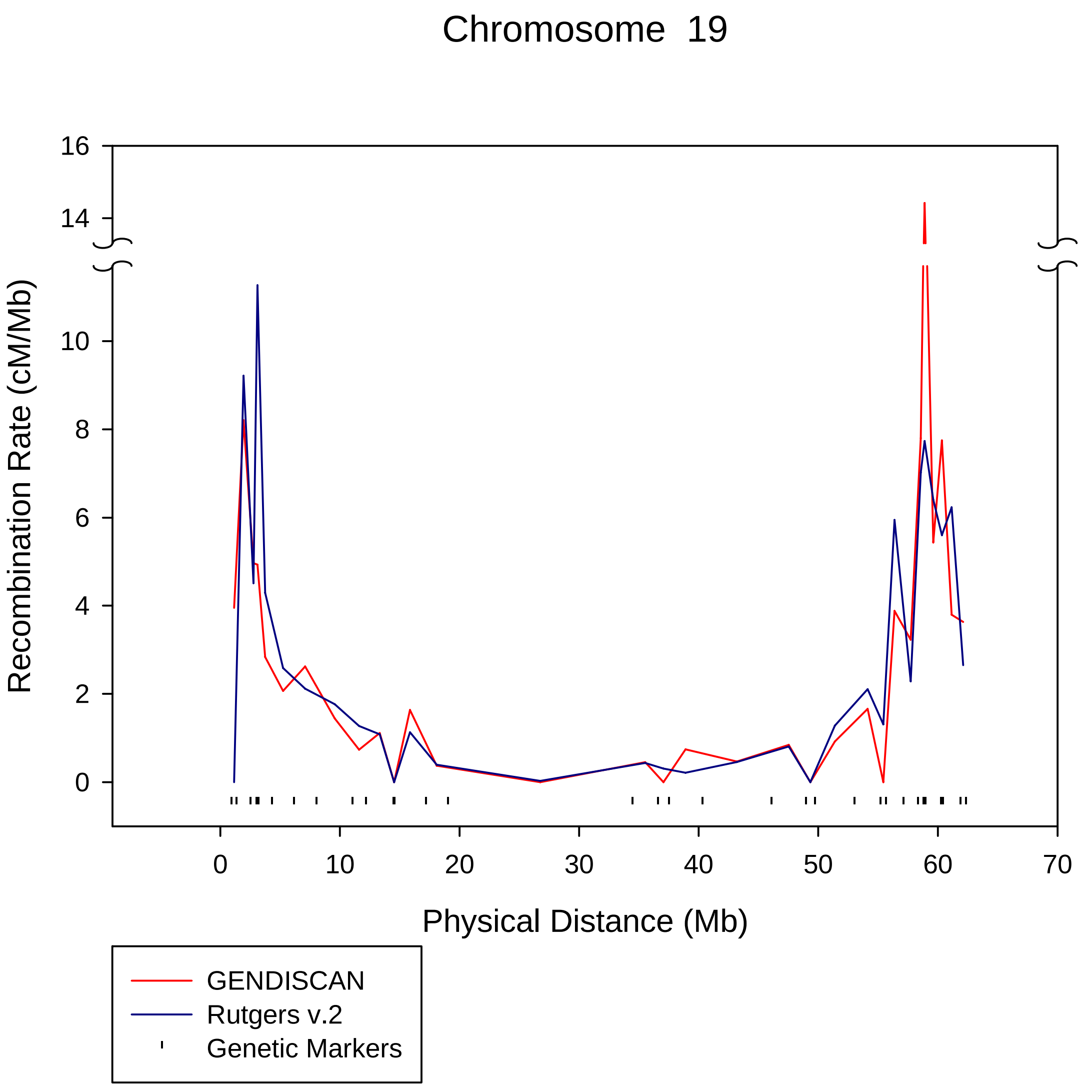
Chromosome 19
16
14
10
8
6
4
2
0
Recombination Rate (cM/Mb)
0
10
20
30
40
50
60
70
Physical Distance (Mb)
GENDISCAN
Rutgers v.2
Genetic Markers

Chromosome 20

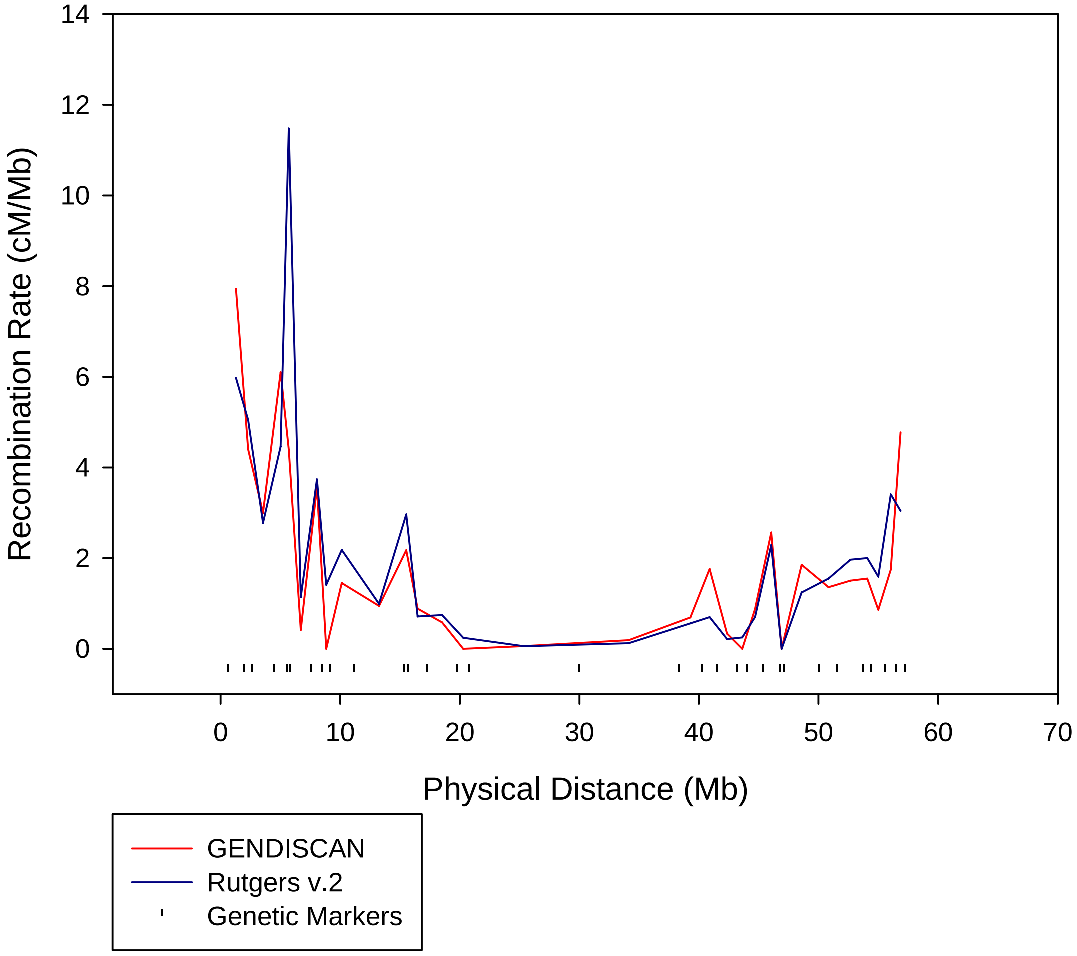

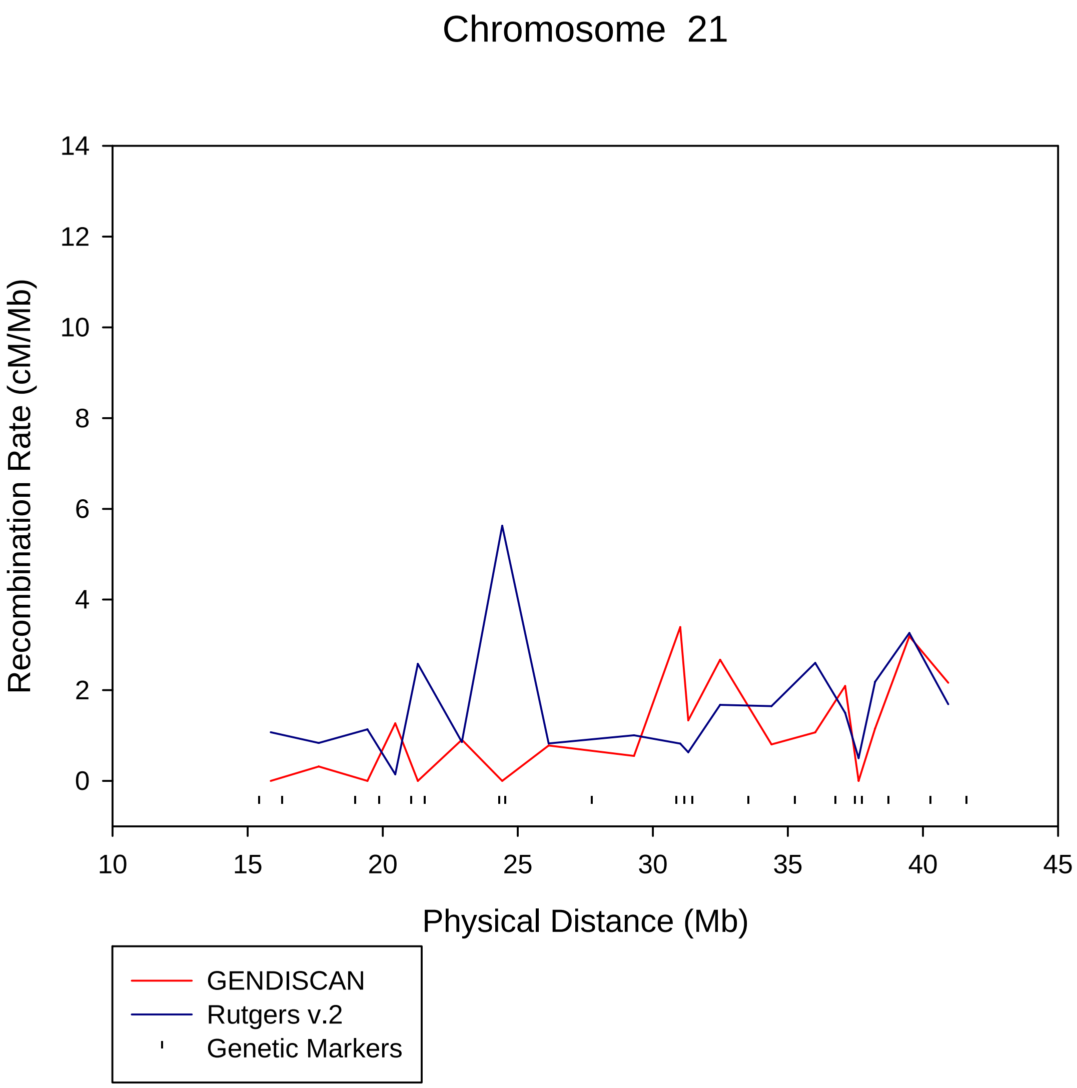

Chromosome 21
Recombination Rate (cM/Mb)
14
12
10
8
6
4
2
0
10
15
20
25
30
35
40
45
Physical Distance (Mb)
GENDISCAN
Rutgers v.2
Genetic Markers

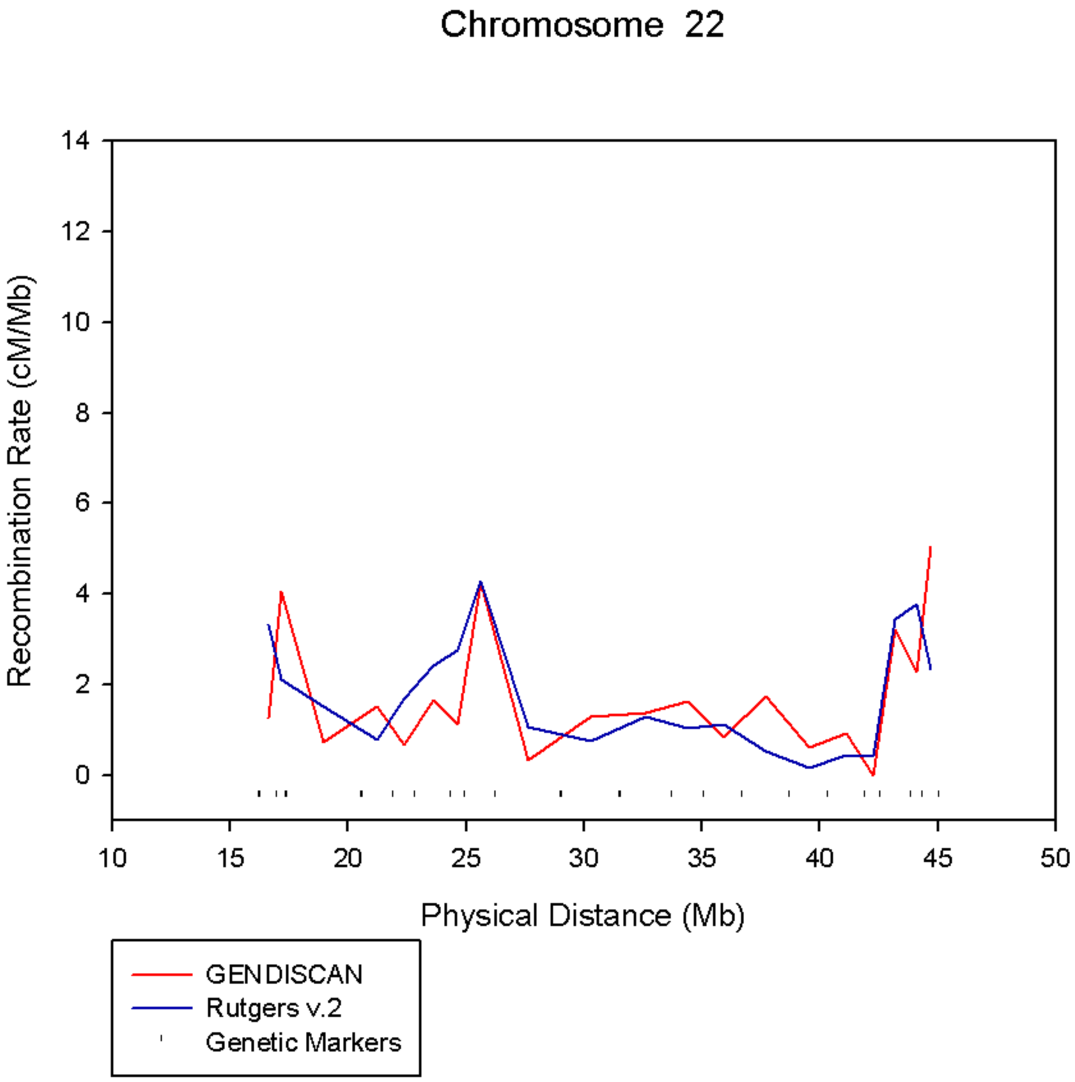

Chromosome 22
Recombination Rate (cM/Mb)
14
12
10
8
6
4
2
0
10
15
20
25
30
35
40
45
50
Physical Distance (Mb)
GENDISCAN
Rutgers v.2
Genetic Markers
